# The Evolutionarily Conserved TPM1 Super‐Enhancer Drives Skeletal Muscle Regeneration via Mechanotransduction Signaling

**DOI:** 10.1002/advs.202514271

**Published:** 2025-11-16

**Authors:** Ruimen Zhang, Wanyou Feng, Yanyan Yang, Yu Pan, Chaoxia Zou, Leyi Wang, Sanbao Zhang, Yimin Zhao, Yongmei Wu, Jinling Wang, Jianwei Zou, Kehe Cen, Yongwang Zhang, Han Huang, Yurong Xu, Li Zhong, Hailong Gong, Juanru Cheng, Jingyuan Liang, Zihua Zheng, Qinyang Jiang, Jingwei Wei, Hui Li, Minhui Liang, Deshun Shi, Sufang Yang, Yanfei Deng, Yingming Wei

**Affiliations:** ^1^ State Key Laboratory for Conservation and Utilization of Subtropical Agro‐Bioresources Guangxi Key Laboratory of Animal Breeding Disease Control and Prevention College of Animal Science and Technology Guangxi University Nanning 530004 China; ^2^ Nanning Normal University Nanning 530023 China; ^3^ School of Mechanical Engineering Guangxi University Nanning 530004 China

**Keywords:** epigenetic regulation, mechanotransduction signaling, skeletal muscle regeneration, TPM1 super‐enhancer

## Abstract

Super‐enhancers (SEs) are critical epigenetic regulators of tissue regeneration, yet their interplay with cellular biomechanics during myogenic differentiation remains unexplored. Here, the *TPM1* locus, encoding a core actin‐stabilizing protein essential for skeletal muscle regeneration, harbors an evolutionarily conserved SE (TPM1_SE) that may bridge epigenetic control and mechanotransduction. In vitro, TPM1_SE deletion impaired myogenic differentiation and diminished expression of both TPM1 and its circular RNA (circRNA) isoform, CircTPM1. Conditional deletion of TPM1_SE significantly reduce muscle mass and delayed regenerative progression. Mechanistically, TPM1_SE drives expression of linear *TPM1* mRNA (mice) and CircTPM1 (bovine) via TEAD4‐mediated chromatin looping, coordinating cytoskeletal reorganization during myotube formation. These effects are mediated via activation of the canonical PI3K/AKT signaling pathway through interaction with *NKX2.2*—a pathway mechanosensitive to cellular tension. Loss of TPM1_SE disrupted NKX2.2‐PI3K/AKT signaling. Crucially, CircTPM1 directly bound MYH10, enhancing MYL3‐dependent actomyosin assembly, which potentiates cytoskeletal reorganization during myotube formation. Collectively, this findings establish TPM1_SE as an evolutionarily conserved hub integrating epigenetic regulation and biomechanical output. While the murine model underscores its therapeutic potential in muscle regenerative medicine, the bovine CircTPM1‐mediated mechanism highlights TPM1_SE as a promising target for genetic improvement of meat quality in livestock.

## Graphical abstract

By integrating biomechanical and epigenetic cues, the evolutionarily conserved TPM1 super‐enhancer drives myogenesis via TEAD4‐mediated chromatin looping. This mechanism produces species‐specific outputs (linear TPM1 mRNA in mice and CircTPM1 in bovine) that activate PI3K/AKT mechanotransduction and the MYH10/MYL3 axis to execute cytoskeletal remodeling, myotube formation, and ultimately, enhanced muscle regeneration.

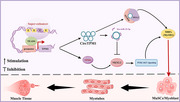

## 1 Introduction

Skeletal muscle regeneration is a highly orchestrated process reliant on muscle stem cells (MuSCs), whose fate is governed by complex epigenetic and transcriptional networks.^[^ ^1^, ^2^, ^3^ ^]^ Among these regulators, super‐enhancers (SEs)—large clusters of transcriptional enhancers with exceptional capacity to drive expression of lineage‐defining genes—have emerged as critical epigenetic arbiters of cell identity and tissue regeneration, including in myogenesis.^[^ ^4^, ^5^ ^]^ Notably, mechanical forces, such as those derived from extracellular matrix stiffness and cellular tension, are increasingly recognized to reshape the epigenetic landscape by modulating SE activity.^[^ ^6^ ^]^ For instance, substrate rigidity modulates activity of transcription factors (TFs) and enhancers via YAP/TAZ‐Hippo signaling,^[^ ^6^, ^7^, ^8^ ^]^ while actomyosin contractility primes SE assembly through force‐dependent chromatin remodeling.^[^ ^9^ ^]^ This interplay suggests that SEs may serve as key nodes integrating biomechanical cues with epigenetic control of regeneration.

SEs have been implicated in processes such as oncogenesis, angiogenesis, and myogenesis.^[^ ^4^, ^5^ ^]^ Recent studies have annotated SE landscapes across humans, mice, and pigs, greatly expanding our understanding of genomic regulatory architecture.^[^ ^10^, ^11^, ^12^ ^]^ Intriguingly, mechanical stress reshapes SE topology: cyclic stretching induces H3K27ac redistribution at muscle‐specific SEs,^[^ ^13^ ^]^ while abnormal ECM stiffness in muscular dystrophies disrupts SE‐driven transcription.^[^ ^14^ ^]^ SEs associated with lineage‐defining genes are known to recruit (TFs) that govern muscle fiber identity and plasticity.^[^ ^4^ ^]^ Notably, SEs influence not only linear mRNA transcription but also circular RNA (circRNA) biogenesis.^[^ ^15^, ^16^ ^]^ The regulation of circRNA production by SEs often involves TF‐mediated promoter hijacking, as exemplified by *circNfix*.^[^ ^17^ ^]^ However, the critical roles and underlying mechanisms of 3D genomic regulatory factors, including chromatin structure, histone modifications, and circRNAs in muscle regeneration remain largely unexplored.

Tropomyosin 1 (*TPM1*), a core actin‐binding protein essential for sarcomere assembly and stability, represents a prime candidate at the nexus of epigenetic regulation and mechanotransduction.^[^ ^18^, ^19^, ^20^ ^]^ It not only stabilizes F‐actin under tension but also modulates cellular stiffness and force generation.^[^ ^21^ ^]^ Intriguingly, the TPM1 locus is marked by a conserved, H3K27ac‐defined SE in differentiating myoblasts,^[^ ^22^ ^]^ suggesting potent epigenetic regulation. However, the function of this SE (hereafter TPM1_SE) in skeletal myogenesis and mechanotransduction remains entirely unexplored.

CircRNAs, characterized by covalently closed‐loop structures, have emerged as critical regulators of gene expression.^[^ ^23^ ^]^ A TPM1‐derived circRNA, circRNA493, has been identified in bovine muscle,^[^ ^24^ ^]^ yet its functional significance and potential regulation by SEs are unknown. Although several circRNAs (circNEB,^[^ ^25^ ^]^ circKANSL1L,^[^ ^26^ ^]^ circACTA1^[^ ^27^ ^]^) regulate myogenesis through diverse mechanisms, a comprehensive understanding of how SE‐driven circRNAs contribute to muscle regeneration is lacking.

Based on this background, we hypothesized that the *TPM1* super‐enhancer (TPM1_SE), its transcriptional regulation, and its interplay with mechanotransduction are integral components of a regulatory axis controlling myogenesis. Here, we bridge this gap by demonstrating that TPM1_SE is an evolutionarily conserved mechano‐epigenetic module essential for muscle regeneration. We show that TPM1_SE drives expression of linear *TPM1* mRNA (mice) and CircTPM1 (bovine) via TEAD4‐mediated chromatin looping, which in turn activates PI3K/AKT signaling and the MYH10/MYL3 axis to promote cytoskeletal reorganization and myotube formation. Our work unveils a novel paradigm wherein a single SE coordinates transcriptional and circRNA outputs to synchronize epigenetic regulation with cellular biomechanics during muscle regeneration.

## 2 Results

### 2.1 Conserved SEs and Their Chromatin Interactions with the TPM1 Locus During Myogenic Differentiation

**Figure 1:**
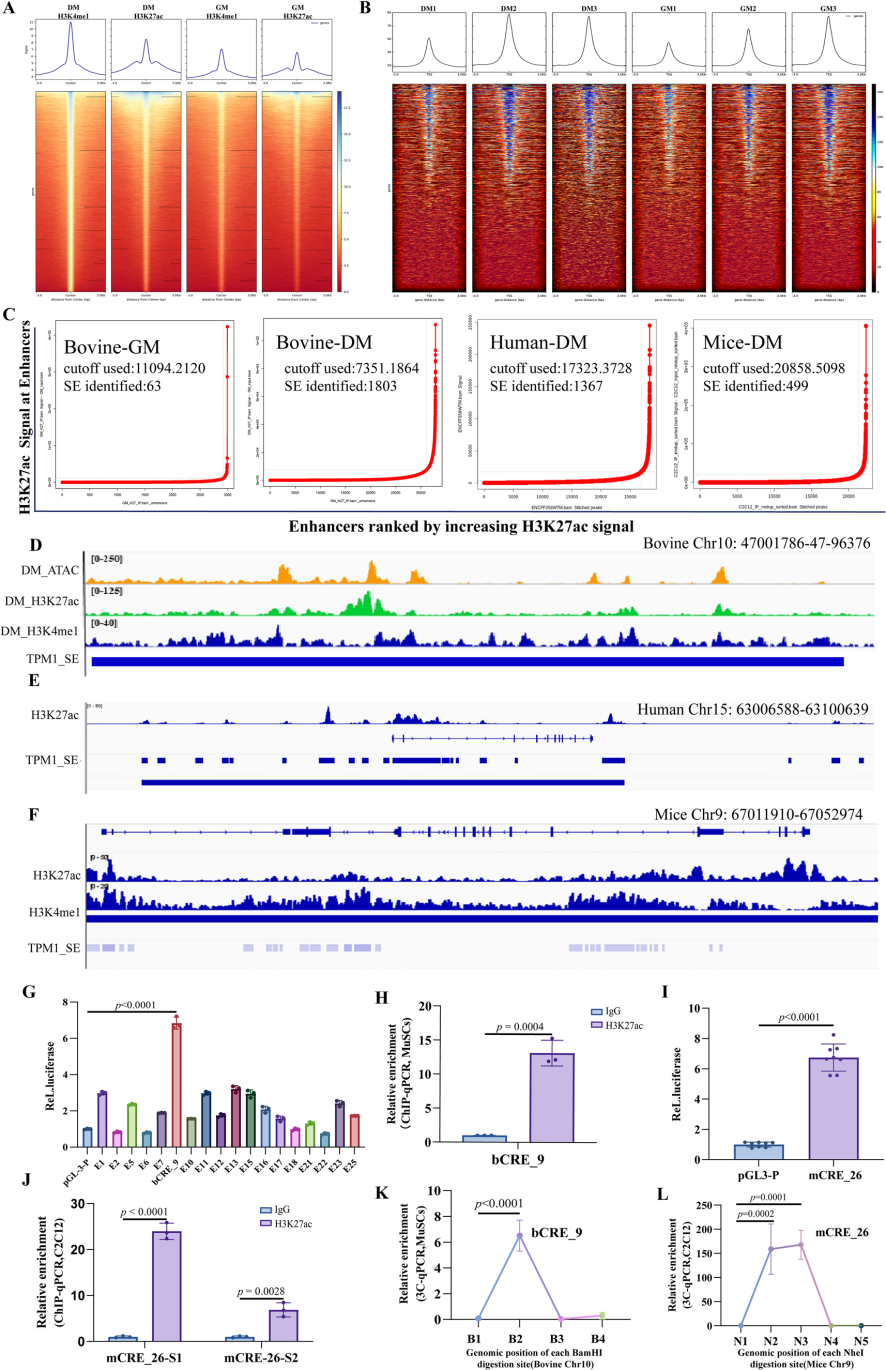
Conserved SEs and chromatin interactions with the *TPM1* locus during myogenic differentiation. A) Differential analysis of H3K27ac and H3K4me1 signals between GM and DM samples was performed using the limma package to rank all SEs and map their distribution around gene transcription start sites TSSs, based on ChIP‐seq data in bovine MuSCs. B) Based on chromatin accessibility profiles from ATAC‐seq, Deep Tools was used to generate line plots and heatmaps showing the distribution of reads relative to TSS positions in GM and DM samples. The results revealed that all six samples exhibited enriched read distributions near the TSS, with the enrichment intensity increasing significantly closer to the TSS. C) Quantification of SEs specifically associated with proliferation or myogenic differentiation in bovine MuSCs‐GM/DM, human skeletal muscle myoblasts‐DM and mice C2C12 myoblasts‐DM. D) Visualization of active histone marks and ATAC signal peaks at the bovine TPM1_SE using IGV. E and F) Comparative chromatin landscape of TPM1_SE in human (E) and mice (F) genomes, annotated with active histone modifications in IGV. G) Luciferase reporter assays demonstrate transcriptional activity of individual elements within bovine TPM1_SE. H) H3K27ac enrichment at bCRE_9 assessed by ChIP–qPCR. I and J) Transcriptional activity (I) and H3K27ac occupancy (J) at mCRE_26 were evaluated via luciferase assay and ChIP–qPCR, respectively. K and L) 3C‐qPCR assays revealed interaction signals between specific anchor and test primers, confirming physical interactions between the TPM1_SE and the *TPM1* promoter. Among the BamH I fragments, the B2 fragment exhibited significantly higher interaction signals (K), which validated the physical interaction between bCRE_9 and the *TPM1* promoter. Similarly, Nhe I fragments covering primers N2 and N3 showed elevated interaction signals (L), confirming the physical interaction between mCRE_26 and the TPM1 promoter. The x‐axis represents the genomic positions of BamH I and Nhe I restriction sites. Notably, 3C‐qPCR signals at B1, B4, N1, and N4 fragments were below the detection limit (Ct > 35), indicating negligible or non‐physiological interactions. Data are presented as mean ± SEM (*n* = 3).

To investigate the remodeling and evolutionary conservation of SEs during muscle regeneration, we analyzed chromatin immunoprecipitation sequencing (ChIP‐seq) and transposase‐accessible‐chromatin with sequencing ATAC‐seq datasets from human skeletal muscle myoblasts (GEO: GSE85169, GSE85171, GSE29611) and mice C2C12 myoblasts (GEO: GSE126147, GSE76010),^[^ ^22^ ^]^ as well as from bovine MuSCs generated in our lab (**Figure** **1**A,B; Figure S1A, B, Supporting Information). These datasets included both proliferative cells (growth medium, GM) and differentiated myotubes (differentiation medium, DM), marked by H3K4me1 and H3K27ac histone modifications. We identified 1,866 in bovine, 1,367 in human, and 499 SEs in mice (Figure 1C; Tables S1–S3, Supporting Information). Across species, 2,135 protein‐coding genes were associated with SEs, with 12 genes—including TPM1, CDK6, and TACC2—identified as conserved candidates (Table S4, Supporting Information). KEGG pathway analysis indicated that these SE‐associated genes contribute to muscle development primarily through the TGF‐β, Hippo, and insulin signaling pathways (*p* < 0.001) (Figure S1C, Table S5, Supporting Information).

*TPM1*, a master regulator of cytoskeletal organization and muscle contractility, was found to harbor a previously unannotated SE in bovine, human, and mice myoblasts (Figure 1D–F), which we termed TPM1_SE. This SE exhibited strong H3K27ac enrichment and was composed of 26 *cis*‐regulatory elements (CREs) in both bovine and murine genomes. Among these, bovine CRE_9 (bCRE_9) and murine CRE_26 (mCRE_26) were identified as orthologous elements. We cloned 18 bovine CREs with prominent ATAC‐seq peaks into a dual‐luciferase reporter vector. Reporter assays revealed that bCRE_9 induced the highest luciferase activity (Figure 1G), and H3K27ac ChIP‐qPCR further validated its high transcriptional activity (*p* < 0.0001) (Figure 1H; Figure S1D, Supporting Information). Similarly, dual‐luciferase assays and H3K27ac ChIP‐qPCR confirmed that mCRE_26 also exhibited strong enhancer activity (*p* < 0.0001) (Figure 1I,J; Figure S1E, Supporting Information). Chromosome conformation capture (3C) assay demonstrated physical interactions between both bCRE_9 and mCRE_26 with the TPM1 promoter (*p* < 0.0001) (Figure 1K,L; Figure S1F–I, Supporting Information). This spatially coordinated architecture suggests that TPM1_SE may orchestrate cytoskeletal remodeling by regulating TPM1‐encoded tropomyosin—a key determinant of actin filament stability and cellular mechanical properties.

### 2.2 Loss of TPM1_SE Impairs Myoblast Differentiation and Transcriptional Activity at the TPM1 Locus

**Figure 2:**
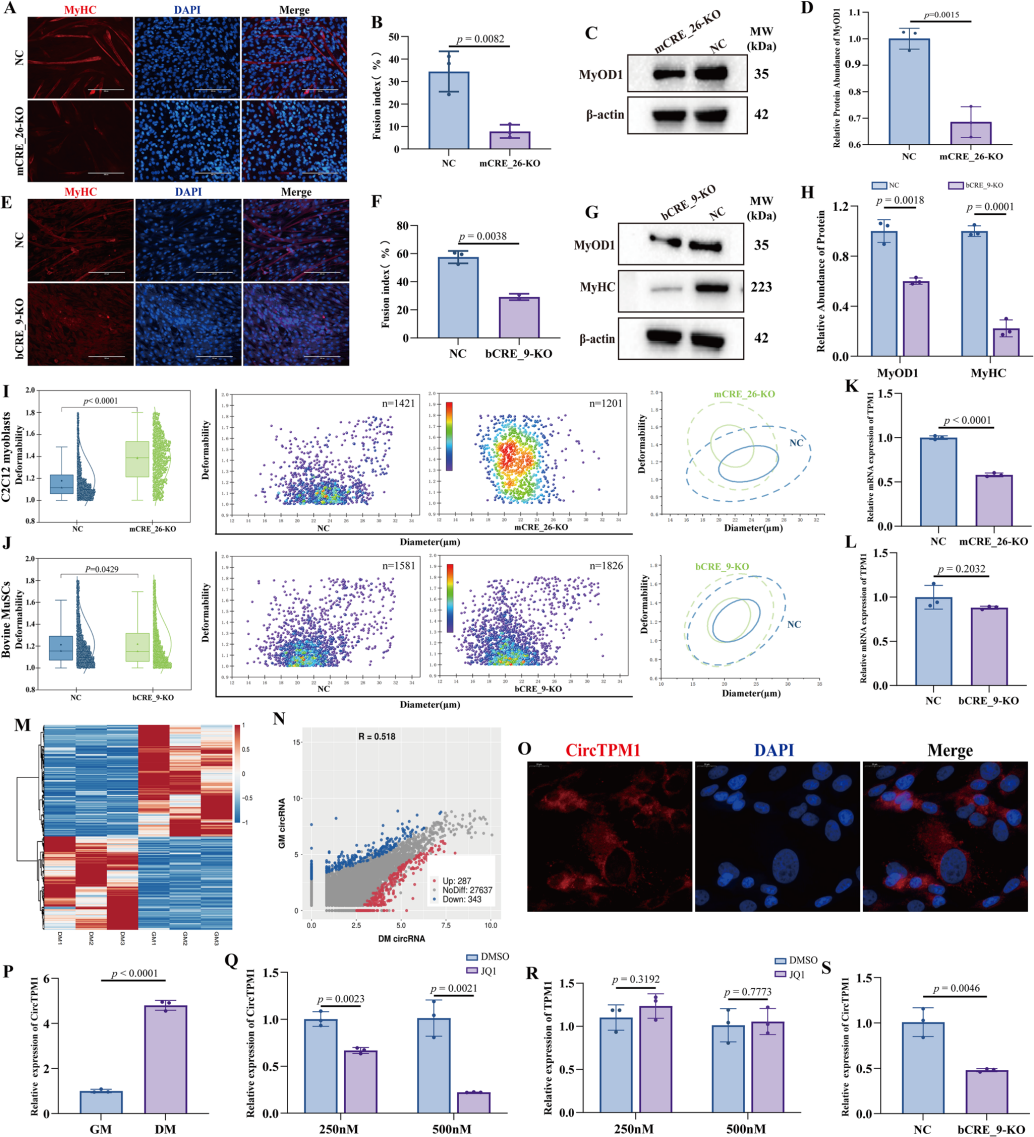
Loss of TPM1_SE impairs myogenic differentiation and *TPM1* transcription. A) Immunofluorescence analysis revealed reduced MyHC‐positive myotubes formation in mCRE_26‐deficient C2C12 myoblasts; DAPI (blue) marks nuclei. B) Fusion index was calculated as the percentage of nuclei in MyHC+ myotubes. Structures containing at least two nuclei are considered as a myotube. C and D) WB analysis and quantification confirmed downregulation of MyOD1 protein levels. E) In bovine MuSCs, deletion of bCRE_9 similarly impaired myogenic differentiation as indicated by decreased MyHC‐positive myotubes. F) Fusion index. G and H) WB and densitometric analysis demonstrated reduced MyOD1 and MyHC proteins expression. I and J) Cell deformability was assessed in mCRE_26‐KO C2C12 myoblasts (I), and bCRE_9‐KO MuSCs (J). The left panel presents a box chart illustrating the deformation values across these groups. The middle panel shows a scatter plot depicting the relationship between deformability and cell diameter. In the right panel, density contour plots are displayed, with solid and dashed lines representing the 50% and 95% density contours, respectively, for comparisons between different treatment groups. Data are from n ≥ 1000 cells per subgroup. Statistical significance was determined using the Shapiro‐Wilk normality test, followed by a Kruskal‐Wallis multiple‐comparison test. K and L) RT‐qPCR revealed that deletion of CRE elements significantly decreased *TPM1* mRNA levels in C2C12 myoblasts but did not affect its levels in bovine MuSCs. M, N) Heatmaps of differentially expressed circRNAs between GM and DM stages in bovine MuSCs identified 630 circRNAs with significant expression changes (log_2_FC > 1, p < 0.05). O) FISH analysis localized CircTPM1 primarily to the cytoplasm in bovine MuSCs. P) RT‐qPCR validated CircTPM1 expression dynamics. Q and R) Treatment of bovine MuSCs with JQ1, a super‐enhancer inhibitor, reduced levels of CircTPM1 without affecting TPM1 mRNA levels, implicating SE dependence. S) Deletion of bCRE_9 further confirmed SE‐mediated regulation of CircTPM1 expression. Data are presented as mean ± SEM (*n* = 3). Scale bars, 200 µm.

To elucidate the functional role of TPM1_SE in myoblast differentiation, we employed CRISPR‐Cas9 genome editing to delete the conserved regulatory elements mCRE_26 in C2C12 myoblasts (Figure S2, Supporting Information) and bCRE_9 bovine MuSCs (Figure S3, Supporting Information). While control (NC) C2C12 myoblasts and MuSCs readily underwent myogenic differentiation, knockout (KO) cells failed to differentiate, as indicated by a decreased number of MyHC‐positive myotubes and nuclei, significantly reduced fusion indices, depressed MyOD1, MyHC proteins (*p* < 0.01) (**Figure** **2**A–H). The inhibited myotube formation in mCRE_26‐KO C2C12 myoblasts and bCRE_9‐KO MuSCs indicated compromised cytoskeletal tension generation. To further investigate whether these defects were associated with altered cellular biomechanics, we employed real‐time deformability cytometry (RT‐DC) to assess cell stiffness. Compared to NC cells, both mCRE_26‐KO C2C12 myoblasts and bCRE_9‐KO MuSCs exhibited significantly greater deformation (Figure 2I,J), indicating a reduction in cell stiffness. Collectively, these findings suggest that TPM1_SE regulates myoblast differentiation through mechanotransduction signaling.

We next investigated whether TPM1_SE modulates transcription at the *TPM1* locus. RT‐qPCR analysis revealed that TPM1 mRNA levels were significantly reduced in mCRE_26‐KO C2C12 myoblasts (*p* < 0.0001) (Figure 2K), indicating that mCRE_26 directly regulates TPM1 transcription. Interestingly, bCRE_9‐KO MuSCs exhibited no change in TPM1 mRNA levels (*p* = 0.205) (Figure 2L), suggesting that bCRE_9 may regulate a non‐coding transcript from the TPM1 locus rather than the mRNA itself.

To explore this hypothesis, we performed circRNA‐seq on bovine MuSCs under GM and DM conditions and identified 630 differentially expressed circRNAs (log_2_FC > 1, *p* < 0.05) (Figure 2M,N; Table S6, Supporting Information). Notably, we discovered a 658‐nt circRNA derived from the TPM1 locus, designated CircTPM1. Validation by PCR, Sanger sequencing, RNase R digestion, and subcellular localization confirmed CircTPM1 as a bona fide circRNA (Figure 2O; Figure S4, Supporting Information), with expression significantly upregulated during myogenic differentiation (*p* < 0.001) (Figure 2P).

To determine whether CircTPM1 expression is regulated by SEs, we treated bovine MuSCs with JQ1, a selective inhibitor of SE function. RT‐qPCR analysis showed that JQ1 treatment significantly reduced CircTPM1 expression (*p* < 0.01) without affecting TPM1 mRNA levels (*p* = 0.782) (Figure 2Q,R), implicating SEs in the selective regulation of CircTPM1. Importantly, CircTPM1 expression was markedly decreased in bCRE_9‐KO MuSCs (*p* < 0.001) (Figure 2S), confirming its regulation by TPM1_SE. Together, these data reveal that TPM1_SE governs myogenic differentiation through transcriptional control of the TPM1 locus, including distinct regulatory outputs: linear mRNA in murine cells and circRNA in bovine cells.

### 2.3 Loss of TPM1_SE Reduces Muscle Weight and Delays Skeletal Muscle Regeneration

**Figure 3:**
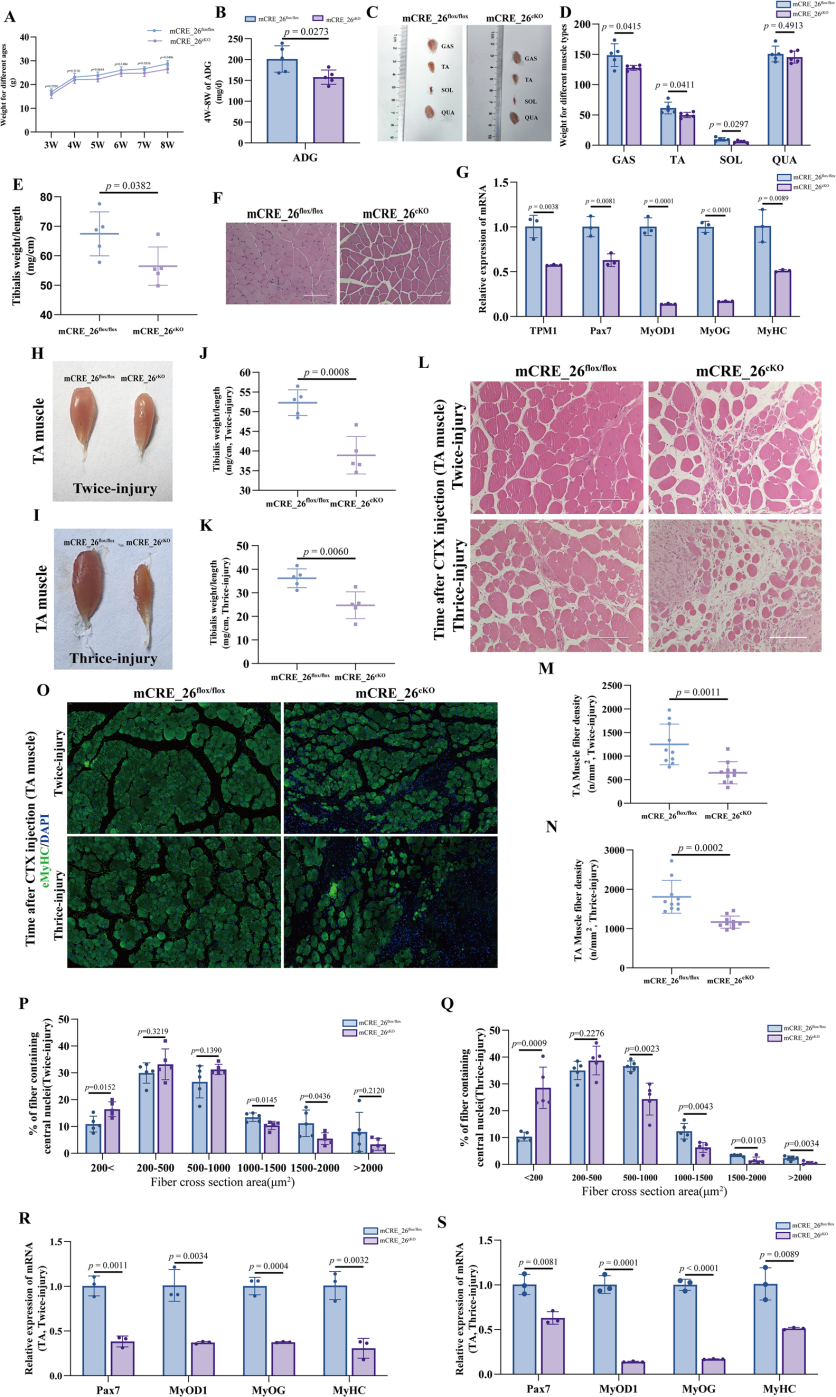
Deletion of TPM1_SE reduces muscle mass and delays skeletal muscle regeneration. A) Longitudinal measurement of body weight in male mice from 3 to 8 weeks. B) Growth rate comparison between mCRE_26^flox/flox^ and mCRE_26^cKO^ mice. C and D) Gross morphology and mass of GAS, TA, SOL, and QUA muscles in adult mCRE_26^flox/flox^ and mCRE_26^cKO^ mice. E) TA muscles weight‐to‐length ratios were calculated to assess muscle integrity. F) H&E staining of TA muscles sections revealed structural alterations in mCRE_26^cKO^ mice (scale bars, 100 µm). G) RT‐qPCR analysis showed decreased expression of TPM1, Pax7, MyOD1, MyOG, and MyHC in knockout mice. H and I) Gross phenotype of TA muscles following cardiotoxin (CTX)‐induced injury in control and knockout groups. Muscles were harvested at day 28 or day 14 after the last CTX injection for secondary or tertiary injury, respectively. J and K) Post‐injury TA muscles weight‐to‐length ratios were compared. L) Histological analysis of regenerating TA muscles via H&E staining; scale bars, 100 µm. M, N) Quantification of myofiber density following injury. O) Representative immunofluorescence images of eMyHC (green, differentiated myofibers), and DAPI (blue, nuclei) in regenerating TA muscle sections. Boxed regions are shown at higher magnification to the right. Scale bars, 20 µm. P and Q) Proportion of regenerated myofibers in CTX‐injured TA muscles cross‐sections quantified from 500 nucleus‐containing fibers using ImageJ and Image‐Pro Plus. R and S) RT‐qPCR validation of muscle regeneration markers Pax7, MyOD1, MyOG, and MyHC. Data are shown as mean ± SEM (*n* = 5).

To determine the functional role of TPM1_SE during muscle regeneration, we focused on the conserved element mCRE_26. We first generated mCRE_26^flox/flox^ mice harboring loxP sites flanking mCRE_26, and then crossed them with Myf5‐Cre mice to create muscle‐specific knockout animals (mCRE_26^cKO^) (Figure S5, Supporting Information). Both mCRE_26^cKO^ and mCRE_26^flox/flox^ mice were viable and fertile. Notably, mCRE_26^cKO^ mice began to exhibit significantly reduced body weights starting at 3 weeks of age (*p* < 0.05) (**Figure** **3**A; Table S7, Supporting Information), and showed a 7.94% decrease in body weight and a 21.63% reduction in growth rate between 4 and 8 weeks compared to controls (*p* < 0.01) (Figure 3A,B).

To determine whether the observed weight loss was attributable to muscle defects, we dissected and weighed major hindlimb muscles. The gastrocnemius (GAS), tibialis anterior (TA), soleus (SOL), and quadriceps femoris (QUA) muscles all exhibited reduced mass in mCRE_26^cKO^ mice by 2.81%, 5.07%, 34.34%, and 3.03%, respectively (Figure 3C,D). Consistent with this, the weight‐to‐length ratio of the TA muscles was also significantly decreased (*p* < 0.05) (Figure 3E). Histological analysis revealed a reduced proportion of myofibers across cross‐sectional areas of the TA muscles (Figure 3F; Figure S6A, Supporting Information). Correspondingly, RT‐qPCR analyses showed significantly lower mRNA levels of TPM1, Pax7, MyOD1, MyOG, and MyHC in TA muscle tissue from mCRE_26^cKO^ mice (*p* < 0.01) (Figure 3G). Given that TPM1 encodes a core structural protein of the sarcomere and was strongly downregulated in the knockout, we assessed its cell‐type specificity. RT‐qPCR confirmed that TPM1 expression was predominantly restricted to myoblasts, with minimal expression in non‐myogenic cell types—including adipocytes, fibroblasts, immune cells, and endothelial cells (Figure S6B), supporting its muscle‐specific role and underscoring the tissue‐autonomous nature of the observed phenotype.

To further investigate mCRE_26's role in MuSC‐mediated regeneration, we induced muscle injury using cardiotoxin (CTX) and monitored regenerative capacity (Figure S6C, Supporting Information). Post‐injury analyses (sampling at 28 days after the last injection for twice‐injury, and 14 days after the last injection for thrice‐injury) revealed pronounced regenerative deficits in mCRE_26^cKO^ mice. The ratio of TA muscles weight to length remained significantly reduced following both secondary and tertiary injuries (Figure 3H,I), and regeneration efficiency decreased by 25.36% and 30.09%, respectively (*p* < 0.001) (Figure 3J,K). Histologically, injured TA muscles sections from mCRE_26^cKO^ mice displayed looser myofiber organization, fewer large fibers (Figure 3L), reduced myofiber density (Figure 3M,N), and diminished proportions of regenerated myofibers (Figure 3P,Q). Moreover, the area of eMyHC⁺ regenerating myofibers were lower post‐injury in mCRE_26^cKO^ mice (Figure 3O; Figure S6D–F, Supporting Information), indicating severe depletion of the stem cells reservoir available for muscle repair. The mRNA expression of key myogenic regulators (Pax7, MyOD1, MyOG, MyHC) remained significantly downregulated (*p* < 0.01) (Figure 3R,S), as assessed at the end of the regeneration timeline to provide a consolidated view of the regenerative transcriptome. Collectively, these findings demonstrate that loss of TPM1_SE impairs MuSCs differentiation capacity and delays skeletal muscle regeneration, highlighting the essential role of mCRE_26 in maintaining muscle mass and regenerative potential.

### 2.4 TPM1_SE Regulates TPM1 Locus Transcription via TEAD4‐Mediated Chromatin Interactions

**Figure 4:**
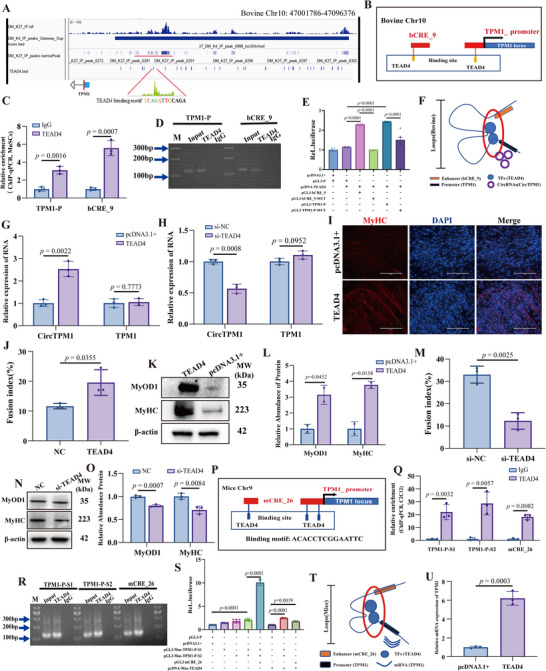
TPM1_SE regulates *TPM1* transcription through TEAD4‐mediated chromatin looping. A and B) ChIP‐seq and ATAC‐seq analyses of bovine MuSCs under GM and DM conditions identified *TEAD4* as a key transcription factor enriched at *TPM1_SE* (A). Bioinformatic predictions revealed potential *TEAD4* binding sites at both bCRE_9 and the *TPM1* promoter (B). C and D) ChIP‐qPCR (C) and agarose gel electrophoresis (D) confirmed TEAD4 binding to bCRE_9 and the *TPM1* promoter. E) Luciferase reporter assays showed that *TEAD4* enhances transcriptional activity when co‐expressed with bCRE_9 and the *TPM1* promoter. F) Chromatin conformation analysis illustrated a *TEAD4*‐facilitated loop linking bCRE_9 and the *TPM1* promoter. G) *TEAD4* overexpression increased CircTPM1 levels without significantly altering linear TPM1 mRNA abundance in bovine MuSCs. H) Interfering with *TEAD4* decreased CircTPM1 levels without significantly altering linear TPM1 mRNA abundance in bovine MuSCs I)Immunofluorescence revealed that *TEAD4* overexpression enhanced myogenic differentiation, evidenced by increased MyHC‐positive myotubes (DAPI, blue). J) Fusion index. K and L) WB and quantitative analysis confirmed that *TEAD4* promotes the expression of MyOD1 and MyHC proteins. M) Fusion index. N and O) WB and quantitative analysis confirmed that interfering with *TEAD4* inhibits the expression of MyOD1 and MyHC proteins. P) Predicted *TEAD4* binding sites were also identified in mCRE_26 and the murine *TPM1* promoter. Q, R), ChIP‐qPCR (Q) and gel electrophoresis (R) confirmed *TEAD4* binding at mCRE_26 and the murine *TPM1* promoter. S) Luciferase assays demonstrated that *TEAD4* activates transcription via the mCRE_26 and murine *TPM1* promoter regions. T) Chromatin interaction mapping further supported a *TEAD4*‐mediated loop between mCRE_26 and the *TPM1* promoter. U) *TEAD4* overexpression increased TPM1 mRNA expression in C2C12 myoblasts. Data are presented as mean ± SEM (*n* = 3). Scale bars, 200 µm.

To uncover the transcriptional mechanisms underlying TPM1_SE activity, we analyzed ATAC‐seq data from bovine MuSCs and murine C2C12 myoblasts (GSE126147). Motif analysis using HOMER and MEME‐ChIP identified conserved *TEAD4* binding motifs at both TPM1_SE and the TPM1 promoter, specifically within the bCRE_9 and mCRE_26 elements (**Figure** **4**A; Table S8, Supporting Information).

In bovine MuSCs, computational scanning predicted a single high‐affinity *TEAD4* motif in both bCRE_9 and the TPM1 promoter (Figure 4B), which was validated by ChIP‐qPCR showing significant *TEAD4* enrichment at both sites (*p* < 0.01) (Figure 4C,D). Consistent with these findings, dual‐luciferase assays demonstrated that *TEAD4* overexpression significantly enhanced transcriptional activity of bCRE_9 and the TPM1 promoter, whereas constructs with mutated *TEAD4* motifs showed no response (*p* < 0.0001) (Figure 4E). These results confirm that bCRE_9 and the promoter form a *TEAD4*‐dependent regulatory module (Figure 4F).

Furthermore, *TEAD4* expression increased during myogenic differentiation in bovine MuSCs (Figure S7A, Supporting Information). Forced expression significantly upregulated CircTPM1 expression (*p* < 0.01) without affecting TPM1 mRNA expression (*p* = 0.968) (Figure 4G; Figure S7B, Supporting Information). Conversely, *TEAD4*‐knockdown resulted in a significant decrease in CircTPM1 expression (Figure 4H; Figure S7C, Supporting Information). *TEAD4* overexpression also enhanced myogenic differentiation and myotube formation of MuSCs (Figure 4I–L; Figure S7D, Supporting Information), indicating that bCRE_9‐mediated regulation of CircTPM1 is TEAD4‐dependent. While control (si‐NC) MuSCs readily underwent myogenic differentiation, *TEAD4*‐knockdown cells failed to differentiate, as indicated by a decreased number of MyHC‐positive myotubes and fusion indices (Figure 4M; Figure S7E, Supporting Information), significantly depressed MyOD1, MyHC proteins (*p* < 0.01) (Figure 4N,O).

In murine cells, in silico analysis predicted one *TEAD4* motif in mCRE_26 and two in the TPM1 promoter (Figure 4P). *TEAD4* ChIP‐qPCR confirmed robust occupancy at mCRE_26 and TPM1 promoter (*p* < 0.001) (Figure 4Q,R). Luciferase assays showed that mCRE_26 significantly increased TPM1 promoter activity, which was further potentiated by *TEAD4* overexpression (*p* < 0.001) (Figure 4S). These findings demonstrate that mCRE_26 and the TPM1 promoter are physically and functionally connected through a *TEAD4*‐mediated chromatin loop (Figure 4T). Consistent with this, *TEAD4* overexpression significantly elevated endogenous *TPM1* mRNA in C2C12 myoblasts (*p* < 0.001) (Figure 4U).

Taken together, our results reveal that TPM1_SE establishes an evolutionarily conserved chromatin architecture through *TEAD4*‐mediated enhancer–promoter looping that regulates the *TPM1* locus. Importantly, despite this conservation, functional divergence exists between species in the type of transcript regulated—TPM1 mRNA in murine cells and CircTPM1 in bovine cells—highlighting species‐specific transcript processing at the same regulatory locus.

### 2.5 TPM1‐Derived RNA Transcripts Promote Myogenic Differentiation and Enhance Skeletal Muscle Regeneration

**Figure 5:**
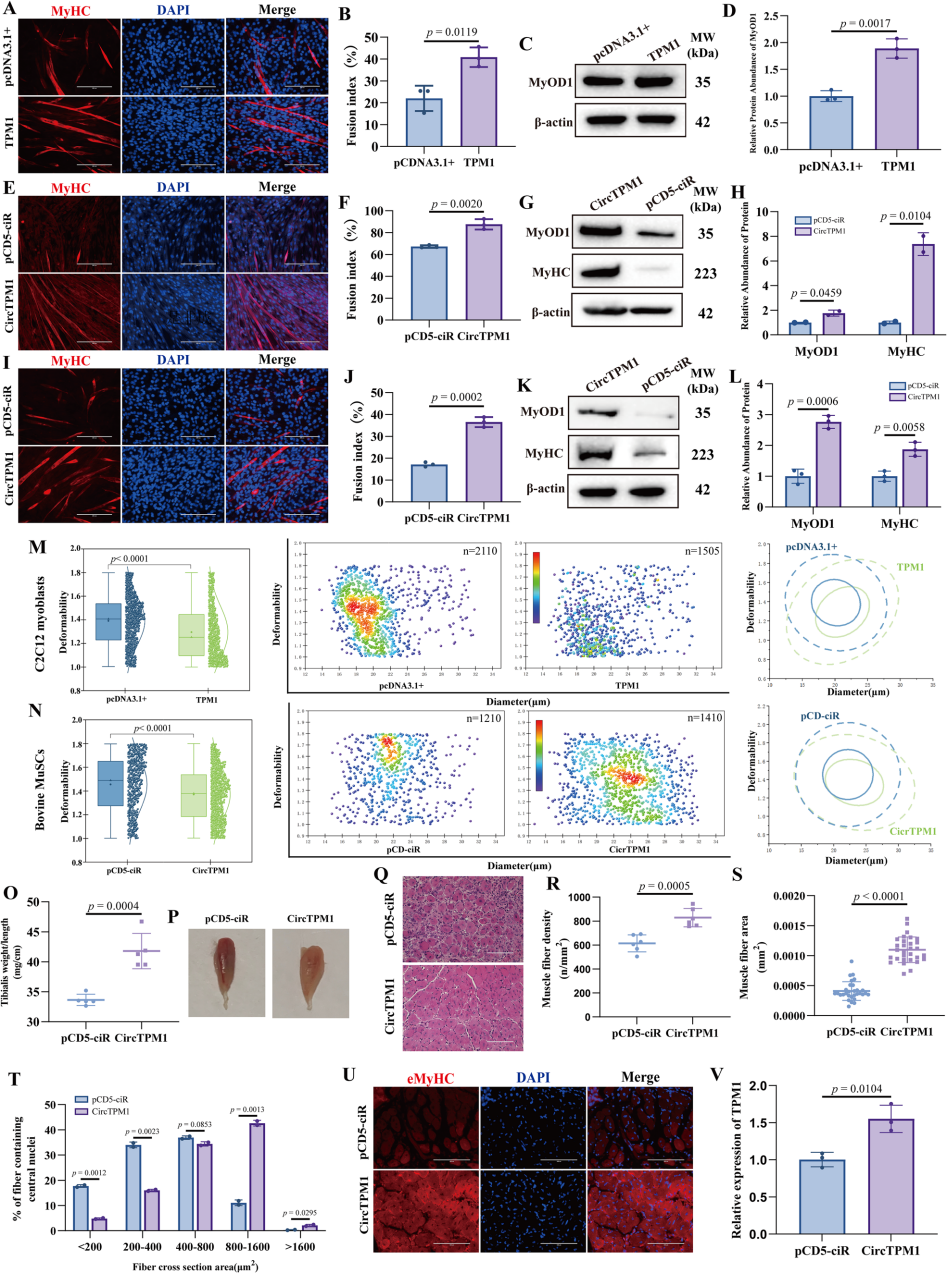
TPM1‐derived RNA transcripts promote myogenic differentiation and skeletal muscle regeneration. A) Overexpression of TPM1 significantly enhances the myogenic differentiation of C2C12 myoblasts, as evidenced by the increased number of MyHC‐positive myotubes visualized via immunofluorescence (DAPI, blue). B) Fusion index. C) WB analysis reveals elevated MyOD1 protein levels following murine TPM1 overexpression. D) Quantification of the WB results in panel C confirms the upregulation of MyOD1. E) Overexpression of CircTPM1 similarly promotes myogenic differentiation in bovine MuSCs, as demonstrated by increased MyHC‐positive myotubes formation (DAPI, blue). F) Fusion index. G) WB analysis shows that CircTPM1 overexpression upregulates MyOD1 and MyHC expression. H) Quantification of WB bands in panel G corroborates this finding. I) In C2C12 myoblasts, CircTPM1 overexpression also facilitates myogenic differentiation, confirmed by a higher number of MyHC‐positive myotubes (DAPI, blue). J) Fusion index. K) WB analysis further indicates increased protein levels of MyOD1 and MyHC. L) Quantification of the WB data in panel K supports these observations. M and N) Cell deformability was assessed in *TPM1*‐overexpressing C2C12 myoblasts (M) and CircTPM1‐overexpressing MuSCs (N). The left panel presents a box chart illustrating the deformation values across these groups. The middle panel shows a scatter plot depicting the relationship between deformability and cell diameter. In the right panel, density contour plots are displayed, with solid and dashed lines representing the 50% and 95% density contours, respectively, for comparisons between different treatment groups. Data are from n ≥ 1000 cells per subgroup. O and P) Measurement of the TA muscles weight‐to‐length ratio revealed a significant increase in the CircTPM1‐treated group. Q) H&E staining of TA muscles cross‐sections demonstrated improved muscle fiber structure following CircTPM1 overexpression. R and S) Quantitative analysis showed that CircTPM1 increased both myofiber density and cross‐sectional area. T) The percentage of regenerated fibers within the TA muscles cross‐sections was determined using ImageJ and Image‐Pro Plus on at least 500 nucleus‐containing myofibers. U) Immunofluorescence analysis confirmed a higher number of eMyHC‐positive myofibers in the CircTPM1‐treated group (DAPI, blue). V) RT‐qPCR also confirmed an increase in murine TPM1 transcript levels. Data are presented as mean ± SEM (*n* = 3–5). Scale bars, 200 µm.

To elucidate the functional roles of TPM1‐derived RNA transcripts in myogenesis, we performed species‐specific overexpression experiments: murine *TPM1* in C2C12 myoblasts and bovine CircTPM1 in MuSCs. Both transcripts were dynamically upregulated during differentiation, mirroring the expression profiles of canonical myogenic markers, including MyOD1, MyOG, and MyHC (Figure S8A,B, Supporting Information). Overexpression of murine *TPM1* in C2C12 myoblasts significantly promoted myogenic differentiation, as evidenced by an increase in MyHC‐positive, multinucleated myotubes, elevated MyOD1 protein levels (*p* < 0.01) (**Figure** **5**A–D), and upregulation of myogenic marker genes at the mRNA level (Figure S8C,D, Supporting Information).

Similarly, stable overexpression of CircTPM1 in bovine MuSCs led to a marked increase in multinucleated MyHC‐positive myotubes and fusion indices, elevated expression of MyOD1 and MyHC at both the protein (*p* < 0.05) (Figure 5E–H) and mRNA levels (Figure S8E, F, Supporting Information). Notably, CircTPM1 overexpression in C2C12 myoblasts also promoted multinucleated myotube formation (Figure 5I–L; Figure S8G,H, Supporting Information), demonstrating its functional conservation.

Accordingly, both murine TPM1 and bovine CircTPM1 promoted myogenic differentiation and myotube formation, suggesting their positive roles in enhancing cytoskeletal tension generation. Consistent with these functional outcomes, RT‐DC revealed that TPM1‐overexpressing C2C12 myoblasts and CircTPM1‐overexpressing MuSCs exhibited reduced cellular deformation compared to control cells (Figure 5M,N), indicating increased cell stiffness and a higher apparent elastic modulus. Collectively, these findings suggest that murine TPM1 and bovine CircTPM1 also contribute to the regulation of myogenic differentiation through mechanotransduction signaling.

We next examined the role of CircTPM1 in vivo using a cardiotoxin (CTX)‐induced muscle injury model. CircTPM1 plasmids were injected into the TA muscles of 6‐week‐old male C57BL/6 mice at days 2 and 4 post‐injury (Figure S8I, Supporting Information). Efficient expression of CircTPM1 in the TA muscles was confirmed (Figure S8J, Supporting Information). Histological analysis at day 12 post‐injury showed that CircTPM1 significantly improved TA muscles weight‐to‐length ratio (Figure 5O), reduced hematoma formation (Figure 5P), and promoted regeneration, as evidenced by increased density, area, and regeneration percentage of myofibers (Figure 5Q–T). Immunofluorescence staining revealed a greater number of eMyHC‐positive MuSCs in CircTPM1‐treated muscles (Figure 5U), accompanied by upregulation of Pax7, MyOD1, MyOG, and MyHC mRNA levels (*p* < 0.001) (Figure S8K, Supporting Information). These data quantitatively confirm enhanced contractile force generation during muscle regeneration, indirectly demonstrating restored biomechanical functionality. Notably, murine TPM1 mRNA levels were also significantly elevated during regeneration (*p* < 0.05) (Figure 5V), further implicating TPM1‐derived transcripts in promoting myogenic differentiation and muscle repair.

### 2.6 TPM1‐Derived Transcripts Promote Myogenesis via the PI3K/AKT Signaling Pathway

**Figure 6:**
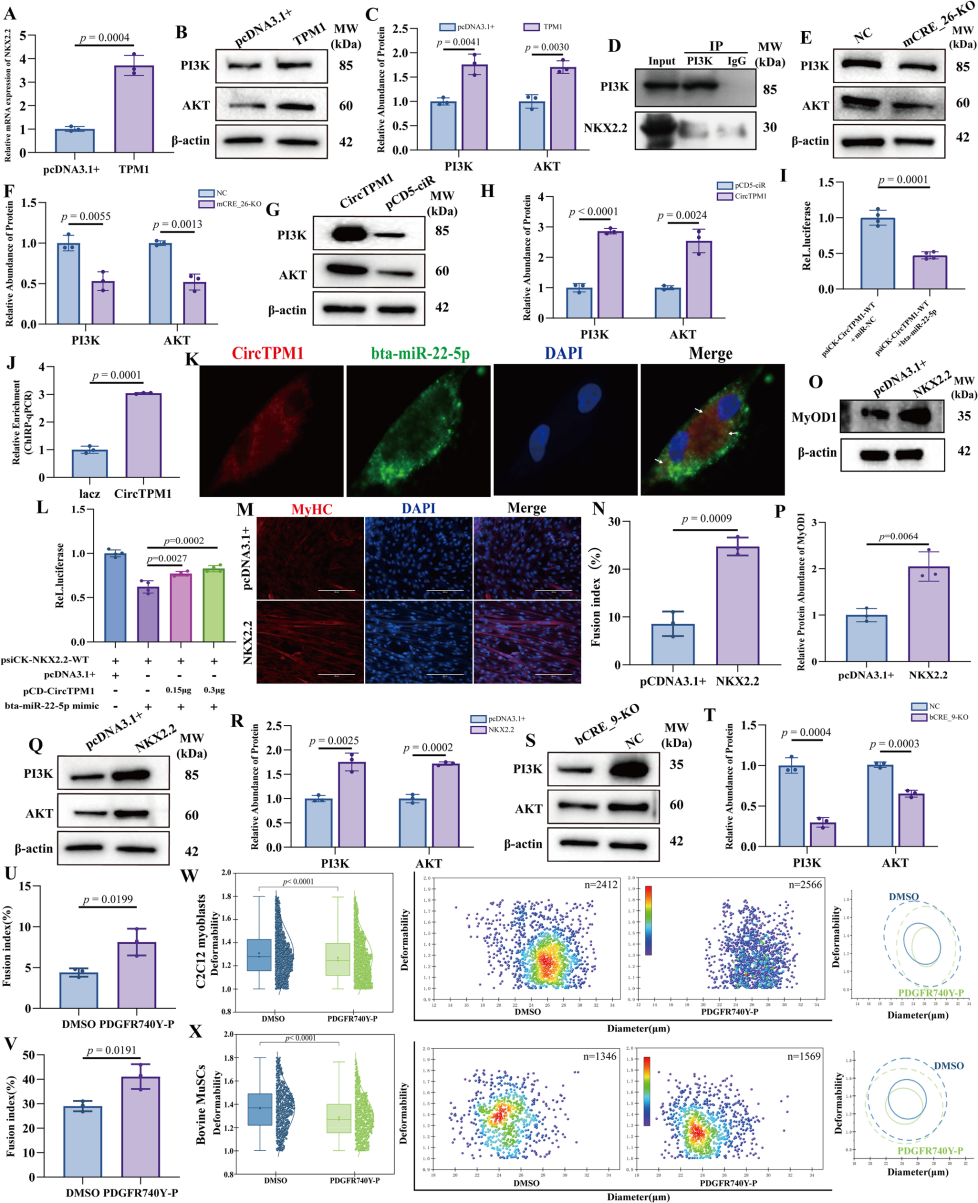
TPM1‐derived RNA transcripts facilitate myogenic differentiation via the PI3K/AKT signaling pathway. A) Overexpression of murine TPM1 led to increased mRNA expression of NKX2.2 in C2C12 myoblasts. B) WB analysis showed that murine TPM1 overexpression also elevated PI3K and AKT protein levels. C) Quantification of panel B confirmed these increases. D) Co‐IP followed by WB analysis demonstrated a direct interaction between PI3K and NKX2.2. E) Knockout of mCRE_26 reduced the expression of PI3K and AKT proteins in C2C12 myoblasts. F) Quantification of the WB data from panel E validated this downregulation. G) In parallel, CircTPM1 overexpression significantly upregulated PI3K and AKT protein levels in bovine MuSCs, as shown by WB. H) Quantitative analysis of panel G confirmed this effect. I) Luciferase reporter assays validated the interaction between CircTPM1 and bta‐miR‐22‐5p. J) ChIRP‐qPCR analysis demonstrated the enrichment of bta‐miR‐22‐5p by CircTPM1 in bovine MuSCs. K) FISH revealed co‐localization of CircTPM1 and bta‐miR‐22‐5p in bovine MuSCs (DAPI, blue; white arrows, the co‐localization of the two). L) Dual‐luciferase reporter assays further indicated that *NKX2.2* interacts with both CircTPM1 and bta‐miR‐22‐5p. M) Overexpression of *NKX2.2* enhanced the differentiation of bovine MuSCs, as indicated by the increased number of MyHC‐positive myotubes. N) Fusion index. O) WB analysis confirmed elevated levels of MyOD1. P) Quantification of the WB data from panel O confirmed the effect of *NKX2.2* overexpression. Q) *NKX2.2* also promoted PI3K and AKT expression at the protein levels in bovine MuSCs, as shown by WB. R) Quantification of the WB data from panel Q corroborated this upregulation. S) In contrast, knockout of bCRE_9 in bovine MuSCs significantly reduced PI3K and AKT protein levels. T) Quantification of WB results in panel S confirmed this downregulation. U and V) Fusion index. W and X) Cell deformability in C2C12 myoblasts (W) and bovine MuSCs (X) were measured upon activation of the PI3K/AKT pathway. The left panel presents a box chart illustrating the deformation values across these groups. The middle panel shows a scatter plot depicting the relationship between deformability and cell diameter. In the right panel, density contour plots are displayed, with solid and dashed lines representing the 50% and 95% density contours, respectively, for comparisons between different treatment groups. Data are from n ≥ 1000 cells per subgroup. Data are presented as mean ± SEM (*n* = 3). Scale bars, 200 µm.

Pathway enrichment analysis indicated a strong association between *TPM1* and the PI3K/AKT signaling pathway, consistent with prior studies.^[^ ^28^ ^]^ The transcription factor NKX2.2, previously validated as a PI3K/AKT activator,^[^ ^29^ ^]^ emerged as a critical mediator. Molecular dynamics simulations revealed spatial and thermodynamic compatibility between murine TPM1, NKX2.2, and PI3K (Figure S9A, Supporting Information). Overexpression of *TPM1* in C2C12 myoblasts led to increased expression of NKX2.2 and PI3K/AKT pathway components (*p* < 0.05) (**Figure** **6**A–C). Co‐immunoprecipitation assays suggested a potential interaction between NKX2.2 and PI3K (Figure 6D), supporting a TPM1–NKX2.2–PI3K/AKT regulatory axis. Correspondingly, deletion of mCRE_26 significantly downregulated PI3K and AKT expression (*p* < 0.01) (Figure 6E,F).

Similarly, CircTPM1 overexpression in bovine MuSCs enhanced PI3K and AKT protein expression (*p* < 0.01) (Figure 6G,H). Integrated bioinformatics and molecular dynamics analyses suggested that CircTPM1 functions through a ceRNA network involving bta‐miR‐22‐5p, *NKX2.2*, and PI3K/AKT signaling (Figure S9B–D, Table S9, Supporting Information).

RT‐qPCR confirmed that CircTPM1 overexpression significantly suppressed bta‐miR‐22‐5p (*p* < 0.01) (Figure S9E, Supporting Information), which itself is downregulated during MuSCs differentiation (*p* < 0.01) (Figure S9F, Supporting Information). Luciferase reporter assays demonstrated that bta‐miR‐22‐5p binds to CircTPM1 and reduces its activity (*p* < 0.01) (Figure 6I), and functionally inhibits myogenic differentiation (Figure S9G–L, Supporting Information). A Chromatin Isolation by RNA Purification (ChIRP) qPCR assay verified the physical interaction between CircTPM1 and bta‐miR‐22‐5p (*p* < 0.0001) (Figure 6J; Figure S9M, Supporting Information), while FISH analysis confirmed their cytoplasmic co‐localization (Figure 6K), reinforcing CircTPM1's role as a miRNA sponge.

To establish the ceRNA regulatory axis, we predicted and experimentally validated NKX2.2 as a direct target of bta‐miR‐22‐5p. Overexpression of bta‐miR‐22‐5p significantly downregulated NKX2.2 (*p* <0.0001) (Figure S9N, Supporting Information), whereas co‐expression of CircTPM1 rescued this repression (Figure S9O, Supporting Information). Dual‐luciferase assays confirmed that CircTPM1 could attenuate the inhibitory effect of bta‐miR‐22‐5p on NKX2.2 (*p* < 0.01) (Figure 6L). This establishes a functional CircTPM1/bta‐miR‐22‐5p/*NKX2.2* axis.

We further confirmed that NKX2.2 was upregulated during myogenic differentiation (*p* < 0.01) (Figure S9P, Supporting Information), and that its overexpression enhances myogenesis (*p* < 0.01) (Figure 6M–P; Figure S9Q, R, Supporting Information) and elevates PI3K and AKT protein levels (*p* < 0.01) (Figure 6Q,R; Figure S9S, Supporting Information), paralleling the effects of CircTPM1. Conversely, deletion of bCRE_9 reduced NKX2.2, PI3K, and AKT expression in bovine MuSCs (*p* < 0.001) (Figure 6S,T; Figure S9T, Supporting Information). Next, we examined whether the PI3K/AKT pathway influences the biomechanical properties of cells during myogenic differentiation. Consistent with its pro‐differentiation role, activation of the pathway in C2C12 myoblasts and bovine MuSCs not only promoted multinucleated myotube formation (Figure 6U,V; Figure S10A–D, I–L, Supporting Information) but also significantly reduced cellular deformation (Figure 6W,X), indicating a concomitant increase in cell stiffness and apparent elastic modulus. In contrast, inhibiting the pathway profoundly blocked differentiation (*p* < 0.001) (Figure S10C, E–H, K, M–P, Supporting Information) and led to a softer cellular phenotype (Figure S10Q, R, Supporting Information), as evidenced by increased deformation and a decreased elastic modulus. Together, these results establish a TPM1_SE→PI3K/AKT→cytoskeletal remodeling axis that bridges epigenetic control with cellular mechanobiology.

### 2.7 CircTPM1 Promotes Myogenic Differentiation by Activating MYL3 Expression via Binding to MYH10

**Figure 7:**
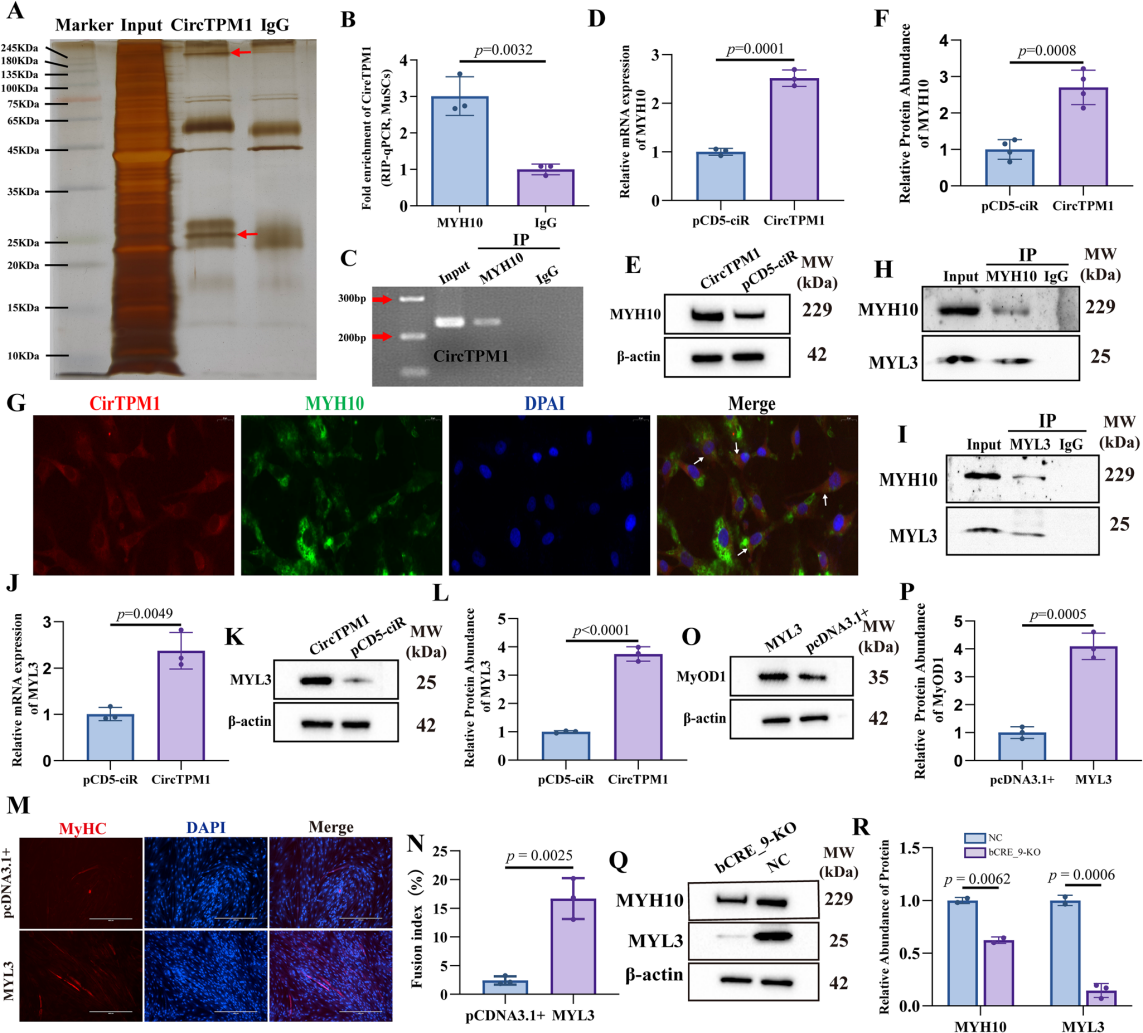
CircTPM1 promotes myogenic differentiation by enhancing *MYL3* expression through direct interaction with MYH10. A) Candidate proteins interacting with CircTPM1 were identified using chromatin isolation by RNA purification coupled with mass spectrometry (ChIRP/MS), revealing MYH10 as a potential binding partner. B and C) The interaction between CircTPM1 and MYH10 was confirmed by RIP‐qPCR (B) and visualized by agarose gel electrophoresis (C), establishing a direct association. D) RT‐qPCR analysis showed that overexpression of CircTPM1 significantly increased MYH10 mRNA levels in bovine MuSCs. E) WB analysis further demonstrated elevated MYH10 protein expression following CircTPM1 overexpression. F) Quantification of the WB data in panel E confirmed the upregulation of MYH10. G) FISH revealed co‐localization of CircTPM1 and MYH10 within bovine MuSCs (DAPI, blue; white arrows, the co‐localization of the two), supporting their physical interaction in situ. H and I) To identify downstream effectors of MYH10, co‐immunoprecipitation followed by WB (Co‐IP/WB) was performed, revealing MYL3 as a MYH10‐interacting protein. The interaction was validated in both forward (H) and reverse (I) directions. J) Overexpression of CircTPM1 led to a significant increase in MYL3 mRNA expression in bovine MuSCs, as assessed by RT‐qPCR. K) Corresponding increases in MYL3 protein levels were observed via WB. L) Quantitative analysis of the WB data in panel K confirmed these results. M) Functional assays demonstrated that *MYL3* overexpression promotes myogenic differentiation in bovine MuSCs, as indicated by an increased number of MyHC‐positive myotubes (DAPI, blue). N) Fusion index. O) WB analysis showed elevated MyOD1 protein levels following *MYL3* overexpression. (P) Quantification of the WB data in panel O validated this upregulation. Q) Disruption of bCRE_9 in bovine MuSCs resulted in significant reductions in both MYH10 and MYL3 protein levels, as shown by WB. R) Quantitative analysis of panel Q confirmed the downregulation. Data are presented as mean ± SEM (*n* = 3). Scale bars, 200 µm.

In addition to its role in a ceRNA network, circRNAs can function as sponges for RBPs, thereby modulating cell differentiation by altering RBPs activity. To explore this alternative mechanism for CircTPM1, we performed a ChIRP assay targeting CircTPM1 (**Figure** **7**A). Unlike conventional ChIRP‐qPCR, this approach retained the protein fraction after crosslinking and precipitation for mass spectrometry analysis, leading to the identification of 261 candidate RBPs (Table S10, Supporting Information). Among these, MYH10 was significantly enriched.

We first verified that the mRNA levels of MYH10 is highly expressed during MuSC myogenic differentiation (*p* < 0.0001) (Figure S11A, Supporting Information). To confirm a direct interaction between CircTPM1 and MYH10, we conducted RNA‐binding protein immunoprecipitation (RIP) qPCR using a MYH10 antibody, which demonstrated significant enrichment of CircTPM1 (*p* < 0.01) (Figure 7B,C). Overexpression of CircTPM1 led to a marked increase in both mRNA and protein levels of MYH10 (*p* < 0.001) (Figure 7D–F). Furthermore, FISH analysis revealed cytoplasmic co‐localization of CircTPM1 and MYH10 (Figure 7G), supporting a direct functional interaction.

Previous studies suggest that MYH10 may influence myogenesis through interactions with other muscle‐related proteins.^[^ ^30^ ^]^ To investigate this, we conducted a Co‐IP assay targeting MYH10, followed by mass spectrometry, and identified 180 interacting proteins. Among them, MYL3 and LDHB were significantly enriched (Table S11, Supporting Information). However, Co‐IP followed by western blotting confirmed interaction only between MYH10 and MYL3 (Figure 7H,I); LDHB did not interact with MYH10 (Figure S11B, Supporting Information).

MYL3 expression was significantly upregulated during myogenic differentiation of MuSCs (*p* < 0.01) (Figure S11C, Supporting Information), and its levels were further increased by CircTPM1 overexpression at both the mRNA and protein (*p* < 0.01) (Figure 7J–L). Functionally, MYL3 overexpression enhanced myogenesis, as evidenced by an increase in MyHC‐positive multinucleated myotubes and fusion indices, (Figure 7M,N), elevated MyOD1 protein levels, and upregulated expression of key myogenic marker genes (Figure 7O,P; Figure S11D, E, Supporting Information). These findings indicate that MYH10 promotes myogenic differentiation primarily through MYL3.

Finally, deletion of the bCRE_9 enhancer significantly reduced expression of both MYH10 and MYL3 in bovine MuSCs (*p* < 0.01) (Figure 7Q,R), further confirming their regulatory linkage. Collectively, these results demonstrate that TPM1_SE promotes myogenic differentiation not only through a ceRNA network but also via an RBP‐mediated mechanism involving the CircTPM1–MYH10/MYL3 axis. This axis facilitates myoblast alignment and fusion—a biomechanically regulated process critical for functional regeneration.

## 3 Discussion

Skeletal muscle is the largest tissue system in mammals, playing essential roles in motor function, metabolic homeostasis, and—in livestock—meat quality and production efficiency.^[^ ^31^ ^]^ Skeletal muscle regeneration is orchestrated by intricate crosstalk between epigenetic programs and biomechanical cues. However, muscle atrophy and functional decline pose serious challenges to healthy aging and the sustainability of animal husbandry.^[^ ^32^, ^33^, ^34^ ^]^ Current research efforts primarily focus on enhancing muscle regeneration to repair damaged tissue and modulating muscle development to improve growth performance.^[^ ^35^ ^]^ Although the regulatory roles of transcription factors (*MyOD1*, *MyOG*), myogenic structural genes (*MYH* family), and non‐coding RNAs (LncRNA‐MEG3, CircDdb1) have been extensively studied,^[^ ^36^, ^37^ ^]^ the mechanisms by which SEs, core chromatin elements that drive transcriptional programs, govern myogenesis and regeneration remain poorly understood.

While our study establishes TPM1_SE as a pivotal epigenetic regulator of myogenesis through transcriptional (*TEAD4*‐mediated looping) and post‐transcriptional (CircTPM1‐dependent) mechanisms, emerging evidence suggests that chromatin dynamics and cellular mechanics engage in bidirectional feedback during tissue repair. Specifically, the *TPM1* locus—encoding core cytoskeletal stabilizers—may serve as a critical node integrating epigenetic and biomechanical signaling. Our data demonstrate that TPM1_SE‐driven expression of linear TPM1 mRNA (mice) and CircTPM1 (bovine) both lead to PI3K/AKT activation. Furthermore, CircTPM1 directly binds to the MYH10/MYL3 complex to facilitate myotube formation. Notably, *TPM1* stabilizes actin filaments to maintain sarcomere integrity,^[^ ^20^ ^]^ directly influencing cellular stiffness and contractile force generation. Loss of TPM1_SE reduced regeneration efficiency and myofiber density by 25–30%, suggesting impaired tension homeostasis.

The identification of TPM1_SE as a conserved regulatory node highlights its central role in skeletal muscle biology. In vivo deletion of TPM1_SE (mCRE_26) in Myf5‐lineage embryonic progenitor cells and their progeny (MuSCs and myofibers) impaired muscle growth and regenerative capacity. TPM1_SE deficiency led to reduced expression of Pax7, MyOD1, and MyHC after injury, along with a significant decline in *Pax7*/*MyHC* double‐positive MuSCs. Given that Pax7 is a key regulator of MuSCs proliferation and self‐renewal, its loss leads to severe deficits in myogenesis.^[^ ^38^ ^]^ These results indicate that TPM1_SE is crucial for maintaining the MuSCs reservoir. This phenotype parallels the regenerative impairments observed in previous knockout models targeting *Tfr1*,^[^ ^39^ ^]^ *IRE1α*,^[^ ^40^ ^]^ and *Metrnl*,^[^ ^41^ ^]^ further underscoring the indispensable role of TPM1_SE in sustaining MuSCs function. In Duchenne muscular dystrophy (DMD), dystrophin enhancer inactivation compromises costamere integrity, reducing myofiber elasticity by >40%.^[^ ^42^ ^]^ Our TPM1_SE knockout model phenocopies this via PI3K/AKT suppression—implying TPM1_SE agonists could restore both epigenetic signaling and tissue mechanics. In livestock, selective breeding for enhanced TPM1_SE activity could improve meat quality and production efficiency by optimizing myofiber tension/stiffness ratios and promoting myofiber hyperplasia. This aligns with the well‐documented success of manipulating Zinc finger BED‐type containing 6 (*ZBED6*) to augment muscle growth, suggesting that CRISPR‐mediated amplification of TPM1_SE could serve as a parallel strategy to enhance muscle growth under intensive farming conditions.^[^ ^43^ ^]^ By targeting TPM1_SE, such interventions may not only refine meat texture through biomechanical adjustments but also support sustainable livestock productivity through enhanced tissue repair and performance resilience.

Consistent with these in vivo findings, *TPM1* mRNA expression was significantly reduced in mCRE_26‐deficient C2C12 myoblasts. In contrast, bovine bCRE_9 knockout did not alter *TPM1* mRNA levels but significantly downregulated CircTPM1 expression. Together, these data suggest that TPM1_SE regulates myogenesis through species‐specific transcript output, with linear mRNA in mice versus circRNA in bovine, highlighting its functional versatility.

The hallmark feature of SEs is their enrichment for master TFs.^[^ ^44^ ^]^ Our data show that chromatin looping between TPM1_SE and the TPM1 promoter is reinforced by *TEAD4* binding, establishing a conserved framework for temporal and spatial gene regulation. Notably, while linear TPM1 mRNA promotes myogenic differentiation in mice, bovine MuSCs rely on CircTPM1, a circRNA derived from the same locus. This divergence illustrates evolutionary plasticity in RNA processing within conserved regulatory landscapes. *TEAD4* overexpression enhanced myogenesis in bovine MuSCs, consistent with earlier reports,^[^ ^45^, ^46^ ^]^ suggesting it may serve as a core activator in muscle lineage commitment across species. Overexpressing CircTPM1 in vivo upregulated TPM1, Pax7, MyOD1, and MyHC mRNA levels and significantly improved muscle regeneration, further positioning the TPM1 locus as a valuable target for enhancing myogenic potential. Furthermore, the mechanism by which mechanical stretch activates H3K27ac enrichment at the TPM1_SE via the YAP/TEAD4 pathway during *TPM1*‐mediated sarcomere assembly warrants further investigation. This mechanism may elucidate the synergistic interplay between mechanical signals and epigenetic regulation in muscle regeneration, providing novel insights into targeted interventions for improving muscle quality.

The PI3K/AKT pathway is widely recognized as a classical mechanosensitive signaling cascade.^[^ ^47^ ^]^ Extensive research has demonstrated its ability to sense and respond to diverse mechanical stimuli, including shear stress, stretch, matrix stiffness, and compressive stress, thereby regulating cell proliferation, differentiation, survival, and metabolism.^[^ ^48^, ^49^, ^50^ ^]^ Bioinformatic analyses predicted *TPM1*’s involvement in the PI3K/AKT signaling pathway, which we functionally validated through molecular dynamics simulations, showing stable binding between murine TPM1 and NKX2.2. We confirmed *NKX2.2* as a direct activator of this pathway, and both murine *TPM1* mRNA and bovine CircTPM1 potentiated PI3K/AKT signaling via *NKX2.2*, driving myogenic differentiation. This dual regulatory paradigm, wherein a single genetic locus modulates signaling through both linear and circular RNA isoforms, represents a previously unrecognized mechanism in skeletal muscle biology. Additionally, we uncovered a novel post‐transcriptional regulatory layer: CircTPM1 acts as a sponge for bat‐miR‐22‐5p, thereby de‐repressing *NKX2.2* and promoting PI3K/AKT activation. This ceRNA mechanism parallels the functions of other myogenic circRNAs, such as circNDST1,^[^ ^51^ ^]^ circACTA1,^[^ ^52^ ^]^ and circMEF2D.^[^ ^53^ ^]^

A seemingly paradoxical aspect of our findings involves the role of the PI3K/AKT pathway. While widely recognized as a potent driver of cell proliferation, it also unequivocally promotes myogenic differentiation.^[^ ^54^, ^55^, ^56^ ^]^ This apparent contradiction underscores the context‐dependent nature of PI3K/AKT signaling, where its functional output is determined by temporal activation, interaction with other pathways, and the cellular microenvironment.^[^ ^56^, ^57^ ^]^ Our data suggest that the strength and duration of PI3K/AKT activation by the TPM1_SE pathway are tailored to engage downstream targets that favor differentiation over those driving proliferation. Consistent with this model, activation of the PI3K/AKT pathway in C2C12 myoblasts and bovine MuSCs not only promoted multinucleated myotube formation but also significantly reduced cellular deformability. Therefore, we propose that the TPM1_SE does not simply act as a generic activator of PI3K/AKT but orchestrates a coordinated transcriptional‐mechanical program. This program ensures that the pro‐differentiation outputs of PI3K/AKT are dominant and coupled with the structural changes necessary for myotube formation, thereby effectively bypassing or overriding its proliferative potential.

Beyond miRNA interactions, circRNAs can act as RBP sponges to influence myogenic pathways.^[^ ^58^ ^]^ We demonstrated that CircTPM1 directly binds MYH10 (non‐muscle myosin IIB), suggesting regulated actomyosin contractility. MYH10 has been previously implicated in muscle development,^[^ ^30^ ^]^ and we further found that it forms a functional axis with MYL3, a myosin light chain family member. *MYL3* promotes myotube fusion in bovine MuSCs and, like TPM3,^[^ ^59^ ^]^ *TNNT1*, and *MYLPF*,^[^ ^60^ ^]^ supports muscle fiber maturation. We propose that CircTPM1‐MYH10/MYL3 axis not only enhances differentiation but also modulates cortical tension in MuSCs, facilitating alignment and fusion during regeneration—a process mechanosensitive to substrate rigidity.

**Figure 8:**
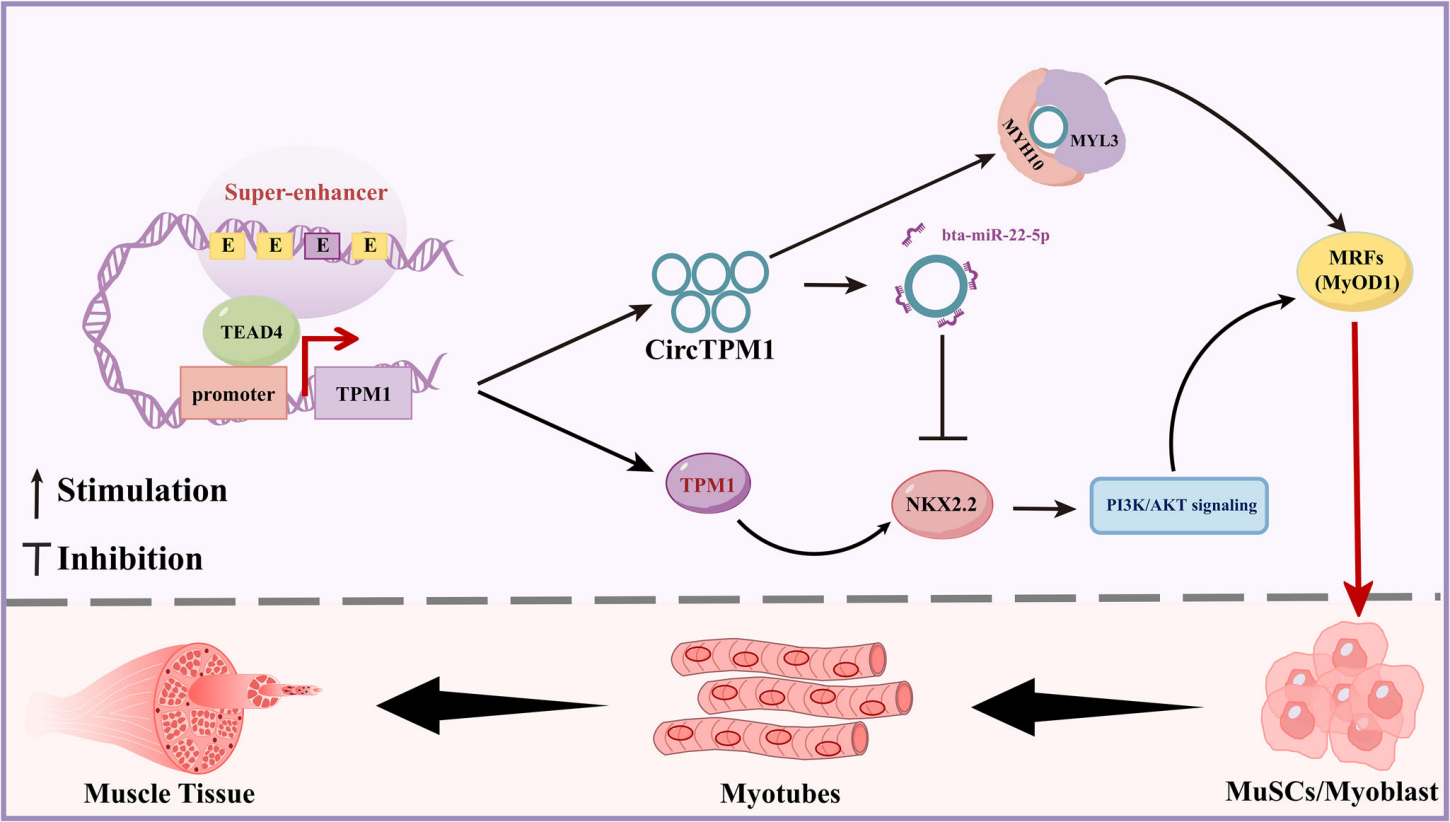
TPM1_SE activates the TPM1 locus to enhance PI3K/AKT signaling and the MYH10/MYL3 axis, thereby driving skeletal muscle regeneration. TPM1_SE activates TPM1 transcription and CircTPM1 formation via TEAD4‐mediated chromatin looping, which in turn enhances PI3K/AKT signaling and MYH10/MYL3 activity to orchestrate cytoskeletal reorganization during myotube formation in skeletal muscle regeneration.

In summary, we identify TPM1_SE as an evolutionarily conserved SE that bridges epigenetic control and mechanotransduction to drive skeletal muscle regeneration. Mechanistically, TPM1_SE activates TPM1 transcription and CircTPM1 biogenesis through TEAD4‐mediated chromatin looping, which in turn enhances PI3K/AKT signaling and MYH10/MYL3 activity to orchestrate cytoskeletal reorganization during myotube formation (**Figure** **8**). These findings establish that augmenting TPM1_SE activity restores mechano‐epigenetic coordination during sarcomere assembly and muscle regeneration. Importantly, although TPM1_SE is functionally conserved between mice and bovine, the species‐specific transcript output—linear *TPM1* mRNA in mice versus CircTPM1 in bovine—underscores divergent translational applications. From a biomedical perspective, our murine model provides a mechanistic basis for targeting TPM1_SE or *TPM1* to develop therapies for human muscle disorders and enhance regeneration. Conversely, in agricultural science, the bovine‐specific TPM1_SE mechanism reveals a novel genetic target for livestock breeding, where enhancing TPM1_SE or CircTPM1 activity holds promise for improving myofiber density, contractile function, and meat production efficiency.

## 4 Experimental Section

### Ethical Standards

All animal experiments were conducted in strict accordance with institutional guidelines for animal welfare. Experimental procedures were approved by the Guangxi University Experimental Animal Ethics Committee (Approval No: Gxu‐2023‐0208) and were carried out at the Animal Experiment Center of the Guangxi Institute for Food and Drug Control, in collaboration with Cyagen Biosciences (Suzhou, China).

### Key Resources Table

C57BL/6 male mice were obtained from Cyagen Biosciences (Suzhou, China) and Beijing Vital River Laboratory Animal Technology Co., Ltd. A complete list of antibodies, reagents, and plasmids used in this study was provided in the Key Resources Table.

### Cell Culture

Bovine MuSCs were isolated as described previously.^[^ ^61^ ^]^ Bovine MuSCs, C2C12 myoblasts, and HEK293T cells were maintained at Guangxi University. Cells were cultured in growth medium (DMEM supplemented with 10% fetal bovine serum and 1% penicillin–streptomycin) at 37 °C in a humidified incubator with 5% CO_2_. Upon reaching ≈80% confluency, bovine MuSCs and C2C12 myoblasts were induced to differentiate by switching to differentiation medium (DMEM with 2% horse serum) for four days.

### Cell Samples

Proliferating C2C12 myoblasts, bovine MuSCs were designated as GM samples, while differentiated myotubes were designated as DM samples. All samples were stored at −80 °C until RNA and DNA extraction.

### Total RNA Extraction

Total RNA was extracted from cells and tissues using the TRIzol reagent, following the manufacturer's protocol. RNA concentration and purity were assessed by spectrophotometry and agarose gel electrophoresis. Only high‐quality RNA was used for downstream analyses.

### Genomic Extraction

Genomic DNA was isolated from cells and tissues using a TD468 Universal Genomic DNA Extraction Kit, in accordance with the manufacturer's instructions.

### ChIP–Seq Analysis

Chromatin immunoprecipitation (ChIP) was performed using a Pierce™ ChIP Kit (Thermo Scientific, USA). Bovine MuSCs in GM and DM phases were harvested (1 × 10⁷ cells per sample; *n* = 3 per group). Cells were sequentially fixed, lysed, and digested, followed by immunoprecipitation using antibodies against H3K4me1 and H3K27ac. After DNA purification, libraries were prepared and sequenced on the Illumina HiSeq™ 2500 platform. Raw reads were base‐called and quality‐controlled according to Illumina's default pipeline, then aligned to the bovine reference genome (Bos_taurus.ARS‐UCD1.2.101) using Bowtie2. Peak calling for enriched H3K4me1 and H3K27ac regions was performed using MACS2 (*p* < 0.05).

### ATAC‐Seq Analysis

To identify differential transcription factors associated with the transition from GM to DM in bovine MuSCs, an integrative analysis of ATAC–seq data was performed (1 × 10⁷ cells per sample; *n* = 3 per group). Genes were categorized into three groups: (1) differentially expressed and differentially accessible; (2) differentially expressed only; and (3) differentially accessible only. Candidate regulatory genes were selected from the first group. Motif enrichment analysis was conducted separately for genes upregulated in DM and GM states, and core transcription factors were ranked by enrichment scores. The reference genome used was Bos_taurus.ARS‐UCD1.2.101 (bosTau9).

### CircRNA‐Seq Analysis

RNA samples from bovine MuSCs in GM and DM phases (1 × 10⁷ cells per sample; *n* = 3 per group) were used for circRNA sequencing. Libraries were constructed from 10 µg of total RNA per sample, following rRNA and linear RNA depletion. Sequencing was performed on the Illumina HiSeq™ 2500 platform. Raw reads were filtered to generate clean reads, which were then mapped to the bovine reference genome (bosTau9). Anchor reads were used to identify circRNAs using the find_circ algorithm. CircRNA characteristics—including type, length distribution, abundance, and chromosomal location—were analyzed. Differential expression analysis was performed using DESeq2. The reference genome used was Bos_taurus.ARS‐UCD1.2.101 (bosTau9).

### SE and circRNA Identification

SEs were identified using the ROSE algorithm based on H3K4me1 and H3K27ac ChIP–seq data. H3K4me1 peaks within 12.5 kb were stitched to define constitutive enhancers. H3K27ac signal intensities (0–300) within these enhancers were quantified from BAM files, and enhancers were ranked to distinguish SEs from typical enhancers. Combining ATAC‐seq data, further screening of SEs involved in chromatin opening. Genes located within 1 Mb of an SE were designated SE target genes, and circRNAs transcribed from these genes were classified as SE‐regulated circRNAs. Visualization was performed using IGV v2.9.4 and Juicebox v1.11.08.

### DNA and RNA Binding Site Analysis

To characterize binding sites within TPM1_SE, the TPM1 promoter, and *TEAD4*, ChIP‐seq and ATAC‐seq datasets were analyzed using HOMER, MEME‐ChIP, and IGV. Based on circRNA‐seq data, circRNA–miRNA–mRNA interactions were predicted using RNAhybrid (v2.1.2) + svm_light (v6.01), Miranda (v3.3a), TargetScan (v7.0), and the RegRNA2.0 platform. Additionally, the UCSC Genome Browser was used to identify human, mice genomic cis‐regulatory elements (CREs) homologous to the bovine TPM1_SE locus. These CREs were cross‐referenced with a mice skeletal muscle‐specific SE database to quantify the number of constitutive enhancers, retrieve enhancer sequences, and identify *TEAD4* binding sites within these regions.

### Reverse Transcription PCR (RT‐PCR)

Reverse transcription was performed using a HiScript II 1st Strand cDNA Synthesis Kit with gDNA wiper. Amplification was carried out with Q5^®^ Hot Start High‐Fidelity 2X Master Mix. PCR products were verified via agarose gel electrophoresis and confirmed by Sanger sequencing. Primer sequences were listed in Table S12 (Supporting Information).

### Real‐Time Quantitative PCR (RT‐qPCR)

RT‐qPCR was conducted using a HiScript R II One Step RT‐PCR Kit and ChamQ SYBR qPCR Master Mix on a LightCycler^®^ 96 Instrument (Roche). β‐actin and U6 served as internal controls. Relative expression levels were calculated using the 2^−ΔΔCt^ method. All primer sequences used were shown in Table S12 (Supporting Information).

### Vector Construction

Construction of overexpression, dual‐luciferase reporter, and CRISPR/Cas9 gene‐editing vectors was performed as previously described.^[^ ^61^ ^]^ Plasmid information was detailed in Key resources table, and corresponding primer sequences were available in Table S12 (Supporting Information).

### Western Blotting (WB) Assay

WB was performed following established protocols.^[^ ^61^ ^]^ Proteins were extracted using RIPA buffer and separated via SDS‐PAGE (12% resolving and 5% stacking gels), followed by PVDF membrane transfer. Membranes were incubated with primary antibodies overnight at 4°C and then with secondary antibodies at room temperature. Protein detection and densitometric analysis were carried out using the ChemiDoc XRS+ system and ImageJ software.

### Immunofluorescent Staining (Cells)

As described previously,^[^ ^61^ ^]^ cells were fixed with 4% paraformaldehyde (PFA), permeabilized with 1% Triton X‐100, blocked with 1% BSA, and sequentially incubated with primary and secondary antibodies, followed by DAPI counterstaining. Images were acquired using a VOS automated cell imaging system.

### Fusion Index

The fusion index was calculated as the percentage of nuclei incorporated into MyHC‐positive myotubes, following established protocols.^[^ ^31^ ^]^ Myotubes were defined as multinucleated structures containing two or more nuclei.

### Mechanical Characterization of Cells

Mechanical characterization of cells was performed using real‐time deformability cytometry (RT‐DC) as previously described.^[^ ^62^ ^]^ Briefly, harvested cell samples were analyzed within a high‐throughput microfluidic system designed for mechanical phenotyping. Initially, randomly distributed cells at the inlet were hydrodynamically focused into a single stream along the central axis and guided toward the electrical sensing zone. When a single cell passed through the detection region, it generated a differential current pulse, which was registered by an impedance analyzer. The analyzer subsequently triggered a high‐speed camera. During the camera acquisition period, the detected cell continued to travel toward a channel bifurcation, where it experienced hydrodynamic stress and underwent deformation. After being recorded, the cell exited the channel. The acquired images of dynamically deforming cells were analyzed to generate two‐dimensional scatter plots depicting initial cell diameter versus deformation capacity.

### Fluorescence In Situ Hybridization (FISH) Assay

FISH was conducted using a commercial in situ hybridization kit according to the manufacturer's instructions.^[^ ^61^ ^]^ Post‐hybridization, nuclei were counterstained, and images were collected using a VOS automated cell imaging system. Probe information was detailed in Table S12 (Supporting Information).

### RNase R Treatment and circRNA Validation

Ribosomal RNA‐depleted samples were treated with RNase R (5 U/µg RNA) at 37 °C for 15 min. CircTPM1 was validated by RT‐PCR and sequencing. Primer sequences were listed in Table S12 (Supporting Information).

### Small Interfering RNA (siRNA) and miRNA Mimics Synthesis

Based on the transcript sequence, three siRNA sequences targeting TEAD4 (RiboBio, Guangzhou, China) was designed and synthesized. In addition, a miRNA mimic targeting bta‐miR‐22‐5p (5’‐ AUUUCGAACGGUGACUUCUUGA‐3’) was also synthesized by the same company.

### Cell Treatments

Bovine MuSCs, C2C12 myoblasts, and HEK293T cells were seeded into plates and cultured to 70–80% confluence. Plasmid transfections were performed using X‐tremeGENE HP DNA Transfection Reagent or electroporation, while miRNA mimics were delivered using X‐tremeGENE siRNA Transfection Reagent. Cells were treated with JQ1 (250 or 500 nM), PDGFR 740Y‐P (50 nM), PI3K/AKT‐IN‐1 (50 nM) or DMSO for 24 hours prior to subsequent analyses.

### Dual‐Luciferase Reporter System Assay

HEK293T cells were seeded into 96‐well plates and co‐transfected after 24 hours with luciferase reporter plasmids, miRNA mimics, and additional constructs using X‐tremeGENE reagent. After 48 hours, luciferase activity was measured using a Luc‐Pair™ Duo‐Luciferase HS Assay Kit.

### Chromatin Isolation by RNA Purification (ChIRP) Assay

ChIRP assays were conducted using a commercial kit according to the manufacturer's protocol. Enriched protein and RNA fractions were analyzed via LC‐MS and RT‐qPCR. All probes and primers were listed in Table S12 (Supporting Information).

### RNA‐Binding Protein Immunoprecipitation (RIP) Assay

RIP was performed using an EZ‐Magna RIP kit. MYH10 antibody and IgG (as a negative control) were employed for immunoprecipitation. After RNA extraction from the immunocomplex, co‐precipitated RNAs were quantified by RT‐qPCR following reverse transcription. Primer sequences were provided in Table S12 (Supporting Information).

### Co‐Immunoprecipitation (Co‐IP) Assay

Proteins were extracted from confluent bovine MuSCs grown in 5 × 10 cm dishes. Lysates were incubated with MYH10, MYL3, PI3K, or IgG antibodies overnight at 4 °C with rotation. Protein complexes were captured using PBS‐washed A/G magnetic beads and incubated for 1 hour at 4 °C. After three washes, the complexes were separated using a magnet and analyzed by WB.

### Chromatin Conformation Capture (3C) Assay

The 3C assay was performed as described by Zuin J *et al.* ^[^ ^63^ ^]^ *BamH* I sites were used to detect potential interactions between the bCRE_9 and TPM1 promoter, while *Nhe* I sites were designed to assess interactions between the mCRE_26 and TPM1 promoter. Ligation products were quantified by RT‐qPCR. All primer sequences were listed in Table S12 (Supporting Information).

### ChIP–qPCR Assay

To validate H3K27ac acetylation levels at promoter and enhancer regions and to assess TF binding, chromatin immunoprecipitation followed by qPCR (ChIP–qPCR) was performed using an EZ‐Magna ChIP A/G Chromatin Immunoprecipitation Kit, following the manufacturer's instructions. Antibodies specific to H3K27ac, TEAD4, and IgG (as a negative control) were employed. DNA‐protein complexes were eluted, and the purified DNA was subjected to RT‐qPCR to quantify enrichment at specific loci. All primer sequences were listed in Table S12 (Supporting Information).

### Generation of CRISPR‐Cas9 Knockout Mice

Conditional knockout mice lacking mCRE_26 (termed mCRE_26^cKO^) were generated using CRISPR/Cas9 and the loxP/Cre recombination system. Fertilized oocytes were produced via in vitro fertilization using sexually mature C57BL/6 mice. mCRE_26^flox/+^ mice were created through co‐microinjection of Cas9 ribonucleoprotein complexes and single‐stranded oligodeoxynucleotides (ssODNs) from Sayer (Suzhou) Biotechnology Co. Ltd. Subsequently, Myf5‐Cre and mCRE_26^flox/+^ mice were crossed with mCRE_26^flox/flox^ mice to generate the mCRE_26^cKO^ mice (mCRE_26^flox/flox; Myf5‐Cre^). Genotypes were confirmed via PCR of tail DNA using primer pairs F1:R1, F2:R2, and F3:R3, yielding 546 bp, 400 bp, and 350 bp bands corresponding to the floxed, WT, and Cre alleles, respectively. All primer sequences were listed in Table S12 (Supporting Information).

### Monitoring of Growth and Muscle Development in Mice

To assess growth dynamics, body weights of male C57BL/6 mice across different genotypes were recorded over time. At 8 weeks of age, mice were fasted overnight and euthanized by decapitation. Skeletal muscles—including the GAS, TA, SOL, and QUA—were harvested from the hind limbs. Tissues were processed for weight‐to‐length (mg/cm) ratio measurements, gene expression profiling, hematoxylin and eosin (H&E) staining, and immunofluorescent analysis.

### Establishment of Muscle Injury Model in Mice

Animal experiments were conducted in SPF conditions at the Guangxi Institute for Food and Drug Control. Muscle injury was induced by intramuscular injection of 50 µL of cardiotoxin (CTX; 1 mg mL^−1^) into the mid‐belly of the TA muscle. Mice were randomized into experimental and control groups (*n* = 5 per group).

In Test 1 (Figure S6C, Supporting Information), to assess muscle injury and regeneration capacity, mCRE_26^cKO^ and mCRE_26^flox/flox^ mice was subjected to multiple rounds of CTX‐induced injury using two distinct experimental schemes: twice‐injury and thrice‐injury paradigms. In the twice‐injury experiment, mice received intramuscular injections of 50 µL CTX on Day 0 and Day 7, and muscle tissues were harvested on Day 28 following the final injection for evaluation of regeneration. In the thrice‐injury experiment, CTX was administered on Day 0, Day 7, and Day 21, with samples collected on Day 14 after the last injection for assessment of muscle regrowth and histological analysis.

In Test 2 (Figure S8I, Supporting Information), 6‐week‐old body weight‐matched male mice were intramuscularly transfected with 6.25 µg of CircTPM1 overexpression plasmid using Entranster™‐in vivo DNA reagent at 2 days and 4 days post‐CTX injection. Control mice received equivalent amounts of the pCD5‐ciR null plasmid. TA muscles were harvested post‐treatment for weight‐to‐length ratio analysis, gene expression assays, and histological evaluation.

### H&E Staining and Myofiber Quantification

Following euthanasia, muscle tissues were collected and fixed in 4% formaldehyde. Paraffin embedding and sectioning were followed by deparaffinization in xylene (I and II for 20 minutes each), dehydration in ethanol (absolute I and II for 5 min each), and rehydration in 5% ethanol for 5 minutes. After rinsing with distilled water, sections were sequentially stained with hematoxylin and eosin, dehydrated, and sealed. Images were acquired using a fully automated VOS cell imaging system. Quantification of myofiber number, diameter, cross‐sectional area (FCSA), and density was performed using ImageJ and Image‐Pro Plus software.

### Immunofluorescent Staining (Muscle tissues)

Immunofluorescence staining of muscle sections was conducted as described by He et al.^[^ ^40^ ^]^ After H&E staining, sections were incubated sequentially with primary antibodies against emyhc (1: 200, Servicebio, Wuhan, China), appropriate secondary antibodies, and DAPI at 4 °C. Fluorescent images were captured using a fully automated VOS cell imaging system.

### Statistical Analysis and Data Reproducibility

Statistical analyses were performed using Excel 2019 and GraphPad Prism 8.0.1 (GraphPad Software, La Jolla, CA, USA). For comparisons between two groups, two‐tailed Student's t‐tests assuming equal variance were employed. Data were presented as mean ± SEM. The number of biological replicates, sample sizes, and specific statistical tests used were indicated in the corresponding Figure legends. Statistical significance was defined as p < 0.05.

## Conflict of Interest

The authors declare no conflict of interest.

## Author Contributions

Conceptualization was performed by R.M.Z., Y.F.D., S.F.Y., and Y.M.W. writing—original draft by R.M.Z., W.Y.F., and Y.Y.Y. Methodology was performed by Y.P., C.X.Z., L.Y.W., and S.B.Z. Supervision was performed by Y.M.W., Y.F.D., S.F.Y., and D.S.S. Project administration was performed by Y.M.W., Y.F.D., S.F.Y., and D.S.S. Visualization was performed by Y.M.Z., Y.M.W., J.L.W., Q.A., Z.H.Z., and Y.Y.Y. Formal analysis was performed by R.M.Z., W.Y.F., Y.Y.Y., J.L.W., K.H.C., J. W. W., and Y.W.Z. Software was performed by R.M.Z., W.Y.F., Y.Y.Y., Y.P., M.H.L, and C.X.Z. Data curation was performed by R.M.Z., H.H., Y.R.X., L.Z., H.L.G, J.R.C., J. W. W., M.H.L, and J.Y.L. Resources was performed by Z.H.Z., Q.Y.J., J.W.W., D.S.S., and Y.M.W. Validation was performed by R.M.Z, W.Y.F., Y.Y.Y., J.L.W., K.H.C., and Y.W.Z. Investigation was performed by R.M.Z, Y.F.D., S.F.Y., Y.P., and C.X.Z. Funding acquisition was performed by Y.M.W., Y.F.D., and S.F.Y. Writing—review and editing was performed by W.Y.F., Y.Y.Y., R.M.Z., Y.F.D., S.F.Y., and Y.M.W.

## Supporting information



Supporting Information

## Data Availability

The data that support the findings of this study are available from the corresponding author upon reasonable request.

## References

[1] A. Kaneshige , T. Kaji , L. Zhang , H. Saito , A. Nakamura , T. Kurosawa , M. Ikemoto‐Uezumi , K. Tsujikawa , S. Seno , M. Hori , Y. Saito , T. Matozaki , K. Maehara , Y. Ohkawa , M. Potente , S. Watanabe , T. Braun , A. Uezumi , S. I. Fukada , Cell Stem Cell 2022, 29, 265. 34856120 10.1016/j.stem.2021.11.003

[2] B. Wang , W. Nie , X. Fu , J. M. de Avila , Y. Ma , M. J. Zhu , M. Maquivar , S. M. Parish , J. R. Busboom , M. L. Nelson , M. Du , J. Anim. Sci. Biotechnol. 2018, 9, 82. 30459947 10.1186/s40104-018-0296-3 PMC6236944

[3] P. Feige , C. E. Brun , M. Ritso , M. A. Rudnicki , Cell Stem Cell 2018, 23, 653. 30388423 10.1016/j.stem.2018.10.006 PMC6262894

[4] K. W. Lee , S. Y. Yeo , J. R. Gong , O. J. Koo , I. Sohn , W. Y. Lee , H. C. Kim , S. H. Yun , Y. B. Cho , M. A. Choi , S. An , J. Kim , C. O. Sung , K. H. Cho , S. H. Kim , Nat. Commun. 2022, 13, 2793. 35589735 10.1038/s41467-022-30484-4 PMC9120014

[5] Y. Zhao , Y. Ding , L. He , Q. Zhou , X. Chen , Y. Li , M. V. Alfonsi , Z. Wu , H. Sun , H. Wang , Sci. Adv. 2023, 9, o1360. 10.1126/sciadv.abo1360 PMC9937580 36800432

[6] B. D. Cosgrove , L. R. Bounds , C. K. Taylor , A. L. Su , A. J. Rizzo , A. Barrera , T. Sun , A. Safi , L. Song , T. Whitlow , A. Tata , N. Iglesias , Y. Diao , P. R. Tata , B. D. Hoffman , G. E. Crawford , C. A. Gersbach , Science 1988, 2025, l. 10.1126/science.adl1988 PMC13005951 40997217

[7] V. A. Codelia , K. D. Irvine , Cell 2012, 150, 669. 22901800 10.1016/j.cell.2012.07.020

[8] S. Dupont , L. Morsut , M. Aragona , E. Enzo , S. Giulitti , M. Cordenonsi , F. Zanconato , J. L. Digabel , M. Forcato , S. Bicciato , N. Elvassore , S. Piccolo , Nature 2011, 474, 179. 21654799 10.1038/nature10137

[9] J. Soto , Y. Song , Y. Wu , B. Chen , H. Park , N. Akhtar , P. Y. Wang , T. Hoffman , C. Ly , J. Sia , S. Wong , D. O. Kelkhoff , J. Chu , M. M. Poo , T. L. Downing , A. C. Rowat , S. Li , Adv. Sci. 2023, 10, 2300152. 10.1002/advs.202300152 PMC10460843 37357983

[10] M. Du , S. H. Stitzinger , J. H. Spille , W. K. Cho , C. Lee , M. Hijaz , A. Quintana , I. I. Cisse , Cell 2024, 187, 331. 38194964 10.1016/j.cell.2023.12.005

[11] S. Ho , T. Sheng , M. Xing , W. F. Ooi , C. Xu , R. Sundar , K. K. Huang , Z. Li , V. Kumar , K. Ramnarayanan , F. Zhu , S. Srivastava , Z. Isa , C. G. Anene‐Nzelu , M. Razavi‐Mohseni , D. Shigaki , H. Ma , A. Tan , X. Ong , M. H. Lee , S. T. Tay , Y. A. Guo , W. Huang , S. Li , M. A. Beer , R. Foo , M. Teh , A. J. Skanderup , B. T. Teh , P. Tan , Gut 2023, 72, 226. 35817555 10.1136/gutjnl-2021-326483

[12] C. Wang , L. Zhang , L. Ke , W. Ding , S. Jiang , D. Li , Y. Narita , I. Hou , J. Liang , S. Li , H. Xiao , E. Gottwein , K. M. Kaye , M. Teng , B. Zhao , Nat. Commun. 2020, 11, 6318. 33298918 10.1038/s41467-020-20136-w PMC7726151

[13] E. R. Hinkle , R. E. Blue , Y. H. Tsai , M. Combs , J. Davi , A. R. Coffey , A. M. Boriek , J. M. Taylor , J. S. Parker , J. Giudice , Commun. Biol. 2022, 5, 987. 36123433 10.1038/s42003-022-03915-7 PMC9485123

[14] P. Martin , C. Pardo‐Pastor , R. G. Jenkins , J. Rosenblatt , Science 2024, 386, p2974. 10.1126/science.adp2974 PMC7617408 39636982

[15] H. I. Suzuki , R. A. Young , P. A. Sharp , Cell 2017, 168, 1000. 28283057 10.1016/j.cell.2017.02.015 PMC5350633

[16] Y. Tan , C. Jiang , Q. Jia , J. Wang , G. Huang , F. Tang , Cell Death Dis. 2022, 13, 401. 35461306 10.1038/s41419-022-04846-1 PMC9035166

[17] S. Huang , X. Li , H. Zheng , X. Si , B. Li , G. Wei , C. Li , Y. Chen , Y. Chen , W. Liao , Y. Liao , J. Bin , Circulation 2019, 139, 2857. 30947518 10.1161/CIRCULATIONAHA.118.038361 PMC6629176

[18] S. S. Halder , M. J. Rynkiewicz , L. Kim , M. E. Barry , A. G. Zied , L. R. Sewanan , J. A. Kirk , J. R. Moore , W. J. Lehman , S. G. Campbell , J. Clin. Invest. 2024, 134, e179135. 39436707 10.1172/JCI179135 PMC11645150

[19] Z. X. Wang , X. Lin , J. Cao , Y. W. Liu , Z. W. Luo , S. S. Rao , Q. Wang , Y. Y. Wang , C. Y. Chen , G. Q. Zhu , F. X. Li , Y. J. Tan , Y. Hu , H. Yin , Y. Y. Li , Z. H. He , Z. Z. Liu , L. Q. Yuan , Y. Zhou , Z. G. Wang , H. Xie , J. Nanobiotechnol. 2024, 22, 208. 10.1186/s12951-024-02367-x PMC11046877 38664789

[20] Y. Aratyn‐Schaus , M. L. Gardel , Science 2008, 322, 1646. 19074337 10.1126/science.1168102

[21] P. Teekakirikul , W. Zhu , X. Xu , C. B. Young , T. Tan , A. M. Smith , C. Wang , K. A. Peterson , G. C. Gabriel , S. Ho , Y. Sheng , D. B. A. Moreau , D. A. Sonnenberg , J. H. Lin , E. Fotiou , G. Tenin , M. X. Wang , Y. L. Wu , T. Feinstein , W. Devine , H. Gou , A. S. Bais , B. J. Glennon , M. Zahid , T. C. Wong , F. Ahmad , M. J. Rynkiewicz , W. J. Lehman , B. Keavney , T. P. Alastalo , et al., Cell Rep. Med. 2022, 3, 100501. 35243414 10.1016/j.xcrm.2021.100501 PMC8861813

[22] Z. Liu , X. Zhang , H. Lei , N. Lam , S. Carter , O. Yockey , M. Xu , A. Mendoza , E. R. Hernandez , J. S. Wei , J. Khan , M. E. Yohe , J. F. Shern , C. J. Thiele , Nat. Commun. 2020, 11, 911. 32060262 10.1038/s41467-020-14684-4 PMC7021771

[23] J. Wei , M. Li , C. Xue , S. Chen , L. Zheng , H. Deng , F. Tang , G. Li , W. Xiong , Z. Zeng , M. Zhou , J. Exp. Clin. Cancer Res. 2023, 42, 86. 37060016 10.1186/s13046-023-02657-6 PMC10105446

[24] X. Wei , H. Li , J. Yang , D. Hao , D. Dong , Y. Huang , X. Lan , M. Plath , C. Lei , F. Lin , Y. Bai , H. Chen , Cell Death Dis. 2017, 8, 3153. 10.1038/cddis.2017.541 PMC5680912 29072698

[25] K. Huang , Z. Li , D. Zhong , Y. Yang , X. Yan , T. Feng , X. Wang , L. Zhang , X. Shen , M. Chen , X. Luo , K. Cui , J. Huang , S. U. Rehman , Y. Jiang , D. Shi , A. Pauciullo , X. Tang , Q. Liu , H. Li , Adv. Sci. 2024, 11, 2300702. 10.1002/advs.202300702 PMC10797441 38036415

[26] Z. Lin , F. Xie , X. He , J. Wang , J. Luo , T. Chen , Q. Jiang , Q. Xi , Y. Zhang , J. Sun , Int. J. Biol. Macromol. 2024, 257, 128609. 38056741 10.1016/j.ijbiomac.2023.128609

[27] A. Qi , W. Ru , H. Yang , Y. Yang , J. Tang , S. Yang , X. Lan , C. Lei , X. Sun , H. Chen , J. Agric. Food Chem. 2022, 70, 3357. 35234473 10.1021/acs.jafc.1c07762

[28] K. Zou , H. Dong , M. Li , Y. Zhang , K. Zhang , D. Song , C. Chu , Genomics 2023, 115, 110687. 37454940 10.1016/j.ygeno.2023.110687

[29] X. Shi , Y. Zheng , L. Jiang , B. Zhou , W. Yang , L. Li , L. Ding , M. Huang , S. Gery , D. C. Lin , H. P. Koeffler , Nucleic Acids Res. 2020, 48, 11434. 33080033 10.1093/nar/gkaa901 PMC7672457

[30] Y. Li , M. Wang , Q. Li , Y. Gao , Q. Li , J. Li , Y. Cao , PLoS One 2020, 15, 235218.

[31] X. Zhang , L. He , L. Wang , Y. Wang , E. Yan , B. Wan , Q. Zeng , P. Zhang , X. Zhao , J. Yin , Sci. Adv. 2024, 10, q6795. 10.1126/sciadv.adq6795 PMC11468980 39999205

[32] K. Huang , Z. Li , D. Zhong , Y. Yang , X. Yan , T. Feng , X. Wang , L. Zhang , X. Shen , M. Chen , X. Luo , K. Cui , J. Huang , S. U. Rehman , Y. Jiang , D. Shi , A. Pauciullo , X. Tang , Q. Liu , H. Li , Adv. Sci. 2024, 11, 2300702. 10.1002/advs.202300702 PMC10797441 38036415

[33] W. Wu , L. Zhang , Y. Chen , C. Huang , L. Yang , D. Lin , Molecules 2025, 30, 2003. 40363810 10.3390/molecules30092003 PMC12073869

[34] Q. Zhao , Y. Jing , X. Jiang , X. Zhang , F. Liu , H. Huang , Z. Zhang , H. Wang , S. Sun , S. Ma , W. Zhang , Y. Yu , X. Fu , G. Zhao , J. Qu , S. Wang , G. H. Liu , Nat. Metab. 2025, 7, 556. 40087407 10.1038/s42255-025-01235-8

[35] W. G. Zhu , A. Thomas , G. M. Wilson , C. Mcglory , J. E. Hibbert , C. G. Flynn , R. Sayed , H. G. Paez , M. Meinhold , K. W. Jorgenson , J. S. You , N. D. Steinert , K. H. Lin , M. J. Macinnis , J. J. Coon , S. M. Phillips , T. A. Hornberger , Nat. Metab. 2025, 7, 1404. 10.1038/s42255-025-01298-7 PMC12875287 40374925

[36] Y. Yao , C. Yan , H. Huang , S. Wang , J. Li , Y. Chen , X. Qu , Q. Bao , L. Xu , Y. Zhang , D. Fan , X. He , Y. Liu , Y. Zhang , Y. Yang , Z. Tang , Adv. Sci. 2025, 12, 2417715. 10.1002/advs.202417715 PMC12199413 40285575

[37] X. Zhu , T. Yang , Y. Zheng , Q. Nie , J. Chen , Q. Li , X. Ren , X. Yin , S. Wang , Y. Yan , Z. Liu , M. Wu , D. Lu , Y. Yu , L. Chen , E. Chatterjee , G. Li , D. Cretoiu , T. S. Bowen , J. Li , J. Xiao , Adv. Sci. 2024, 11, 2406986. 10.1002/advs.202406986 PMC11615752 39412095

[38] M. E. Esper , C. E. Brun , A. Lin , P. Feige , M. J. Catenacci , M. C. Sincennes , M. Ritso , M. A. Rudnicki , J. Cachexia Sarcopenia Muscle 2025, 16, 13682. 10.1002/jcsm.13682 PMC11669949 39723578

[39] H. Ding , S. Chen , X. Pan , X. Dai , G. Pan , Z. Li , X. Mai , Y. Tian , S. Zhang , B. Liu , G. Cao , Z. Yao , X. Yao , L. Gao , L. Yang , X. Chen , J. Sun , H. Chen , M. Han , Y. Yin , G. Xu , H. Li , W. Wu , Z. Chen , J. Lin , L. Xiang , J. Hu , Y. Lu , X. Zhu , L. Xie , J. Cachexia Sarcopenia Muscle 2021, 12, 746. 33955709 10.1002/jcsm.12700 PMC8200440

[40] S. He , T. Fu , Y. Yu , Q. Liang , L. Li , J. Liu , X. Zhang , Q. Zhou , Q. Guo , D. Xu , Y. Chen , X. Wang , Y. Chen , J. Liu , Z. Gan , Y. Liu , J. Clin. Invest. 2021, 131, e143737. 34283807 10.1172/JCI143737 PMC8409588

[41] G. S. Baht , A. Bareja , D. E. Lee , R. R. Rao , R. Huang , J. L. Huebner , D. B. Bartlett , C. R. Hart , J. R. Gibson , I. R. Lanza , V. B. Kraus , S. G. Gregory , B. M. Spiegelman , J. P. White , Nat. Metab. 2020, 2, 278. 32694780 10.1038/s42255-020-0184-y PMC7504545

[42] D. Mazala , R. Hindupur , Y. J. Moon , F. Shaikh , I. H. Gamu , D. Alladi , G. Panci , M. Weiss‐Gayet , B. Chazaud , T. A. Partridge , J. S. Novak , J. K. Jaiswal , Cell Death Discov. 2023, 9, 224. 37402716 10.1038/s41420-023-01503-0 PMC10319851

[43] C. Wei , D. Wang , Y. Ma , S. Wang , D. Pan , Y. Ma , L. Jiang , J. Cachexia Sarcopenia Muscle 2025, 16, 13829. 10.1002/jcsm.13829 PMC12134784 40464206

[44] Y. Tan , Y. Li , F. Tang , Mol. Cancer 2020, 19, 74. 32278350 10.1186/s12943-020-01195-5 PMC7149907

[45] N. Figeac , A. D. Mohamed , C. Sun , M. Schonfelder , D. Matallanas , A. Garcia‐Munoz , E. Missiaglia , E. Collie‐Duguid , V. De Mello , A. V. Pobbati , J. Pruller , O. Jaka , S. Harridge , W. Hong , J. Shipley , N. Vargesson , P. S. Zammit , H. Wackerhage , J. Cell Sci. 2019, 132, jcs225946. 31138678 10.1242/jcs.225946 PMC6633393

[46] J. Wang , F. Zhang , H. Yang , H. Wu , R. Cui , Y. Zhao , C. Jiao , X. Wang , X. Liu , L. Wu , G. Li , X. Wu , Am. J. Transl. Res. 2018, 10, 998. 29636889 PMC5883140

[47] N. Borreguero‐Munoz , G. C. Fletcher , M. Aguilar‐Aragon , A. Elbediwy , Z. I. Vincent‐Mistiaen , B. J. Thompson , PLoS Biol. 2019, 17, 3000509. 10.1371/journal.pbio.3000509 PMC6814241 31613895

[48] F. Song , D. Jiang , T. Wang , Y. Wang , Y. Lou , Y. Zhang , H. Ma , Y. Kang , Biomed Res. Int. 2017, 2017, 6027402. 28286769 10.1155/2017/6027402 PMC5329655

[49] Y. Da , Y. Mou , M. Wang , X. Yuan , F. Yan , W. Lan , F. Zhang , Mol. Med. Rep. 2020, 21, 470. 31746379 10.3892/mmr.2019.10808

[50] E. M. Small , J. R. O'Rourke , V. Moresi , L. B. Sutherland , J. Mcanally , R. D. Gerard , J. A. Richardson , E. N. Olson , Proc. Natl. Acad. Sci. USA 2010, 107, 4218. 20142475 10.1073/pnas.1000300107 PMC2840099

[51] Z. Yang , C. Song , R. Jiang , Y. Huang , X. Lan , C. Lei , X. Qi , C. Zhang , B. Huang , H. Chen , J. Agric. Food Chem. 2022, 70, 10044. 35916743 10.1021/acs.jafc.1c08167

[52] A. Qi , W. Ru , H. Yang , Y. Yang , J. Tang , S. Yang , X. Lan , C. Lei , X. Sun , H. Chen , J. Agric. Food Chem. 2022, 70, 3357. 35234473 10.1021/acs.jafc.1c07762

[53] X. Zhang , S. Yang , Z. Kang , W. Ru , X. Shen , M. Li , X. Lan , H. Chen , J. Agric. Food Chem. 2022, 70, 8145. 35749701 10.1021/acs.jafc.2c01888

[54] C. Serra , D. Palacios , C. Mozzetta , S. V. Forcales , I. Morantte , M. Ripani , D. R. Jones , K. Du , U. S. Jhala , C. Simone , P. L. Puri , Mol. Cell 2007, 28, 200. 17964260 10.1016/j.molcel.2007.08.021 PMC2693200

[55] B. Chatterjee , D. W. Wolff , M. Jothi , M. Mal , A. K. Mal , Skelet. Muscle 2016, 6, 28. 27551368 10.1186/s13395-016-0100-z PMC4993004

[56] X. Chen , Y. Zhang , Z. Deng , C. Song , L. Yang , R. Zhang , P. Zhang , Y. Xiu , Y. Su , J. Luo , J. Xu , H. Dai , J. Cachexia Sarcopenia Muscle 2025, 16, 13724. 10.1002/jcsm.13724 PMC11832428 39962589

[57] A. M. Singh , D. Reynolds , T. Cliff , S. Ohtsuka , A. L. Mattheyses , Y. Sun , L. Menendez , M. Kulik , S. Dalton , Cell Stem Cell 2012, 10, 312. 22385658 10.1016/j.stem.2012.01.014 PMC3294294

[58] X. Li , Y. Yang , Z. Wang , X. Lin , X. Fu , X. He , M. Liu , J. X. Wang , T. Yu , P. Sun , J. Adv. Res. 2025, 69, 329. 38621622 10.1016/j.jare.2024.04.011 PMC11954820

[59] M. R. Lambert , E. Gussoni , Skelet. Muscle 2023, 13, 18. 37936227 10.1186/s13395-023-00327-x PMC10629095

[60] P. Kaewsatuan , C. Poompramun , S. Kubota , J. Yongsawatdigul , W. Molee , P. Uimari , A. Molee , Poult. Sci. 2023, 102, 102741. 37186966 10.1016/j.psj.2023.102741 PMC10192536

[61] R. Zhang , Y. Pan , W. Feng , Y. Zhao , Y. Yang , L. Wang , Y. Zhang , J. Cheng , Q. Jiang , Z. Zheng , M. Jiang , S. Yang , Y. Deng , D. Shi , Y. Wei , J. Agric. Food Chem. 2022, 70, 9166. 35837734 10.1021/acs.jafc.2c03384

[62] M. Liang , D. Yang , Y. Zhou , P. Li , J. Zhong , Y. Ai , Anal. Chem. 2021, 93, 4567. 33661609 10.1021/acs.analchem.0c05009

[63] J. Zuin , G. Roth , Y. Zhan , J. Cramard , J. Redolfi , E. Piskadlo , P. Mach , M. Kryzhanovska , G. Tihanyi , H. Kohler , M. Eder , C. Leemans , B. van Steensel , P. Meister , S. Smallwood , L. Giorgetti , Nature 2022, 604, 571. 35418676 10.1038/s41586-022-04570-y PMC9021019

