# Supplementary material for: The Evolutionarily Conserved TPM1 Super‐Enhancer Drives Skeletal Muscle Regeneration via Mechanotransduction Signaling
Source: Adv Sci (Weinh). 2025 Nov 16;13(10):e14271. doi: 10.1002/advs.202514271 (PMC12915160; doi:10.1002/advs.202514271)
Supplement: Supplementary file 1 — Supporting Information [file ADVS-13-e14271-s001.docx]

**Supplementary Materials for**

**The Evolutionarily Conserved TPM1 Super-Enhancer Drives Skeletal Muscle Regeneration via Mechanotransduction Signaling**

Ruimen Zhang^1^, Wanyou Feng^1,2^, Yanyan Yang^1^, Yu Pan^1^, Chaoxia Zou^1^, Leyi Wang^1^, Sanbao Zhang^1^, Yimin Zhao^1^, Yongmei Wu^1^, Jinling Wang^1^, Jianwei Zou^1^, Kehe Cen^1^, Yongwang Zhang^1^, Han Huang^1^, Yurong Xu^1^, Li Zhong^1^, Hailong Gong^1^, Juanru Cheng^1^, Jingyuan Liang^1^, Zihua Zheng^1^, Qinyang Jiang^1^, Jingwei Wei^1^, Hui Li^1^, Minhui Liang^3^, Deshun Shi^1^, Sufang Yang ^1*^, Yanfei Deng^1*^ and Yingming Wei ^1*^

^1^State Key Laboratory for Conservation and Utilization of Subtropical Agro-Bioresources, Guangxi Key Laboratory of Animal Breeding, Disease Control and Prevention, College of Animal Science and Technology, Guangxi University, Nanning 530004, China.

^2^Nanning Normal University, Nanning 530023, China.

^3^School of Mechanical Engineering, Guangxi University, Nanning 530004, China.

**Correspondence:** [ysfang3511@163.com(S.F.Y)](mailto:ysfang3511@163.com(S.F.Y)), [yanfei-dun@163.com(Y.F.D.)](mailto:yanfei-dun@163.com(Y.F.D.)),

[dkywym@163.com](mailto:dkywym@163.com)(Y. M.W.)

**The PDF file includes:**

**Table of Contents**

[Supplementary Note 2](#_Toc201585484)

[Description of ChIP-seq, ATAC-Seq data and SE on bovine MuSCs 2](#_Toc201585485)

[Description of TPM1_SE conditional knockout mice model 3](#_Toc201585486)

[Impact of the TPM1_SE on muscle development in mice 4](#_Toc201585487)

[Impact of TPM1_SE on myogenic differentiation 4](#_Toc201585488)

[Identification of CircTPM1 4](#_Toc201585489)

[Validation of TPM1_SE, *TPM1* locus, and *TEAD4* 5](#_Toc201585490)

[Supplementary Figures 6](#_Toc201585491)

[Uncropped Western blots 19](#_Toc201585492)

[Supplementary table caption 29](#_Toc201585493)

# Supplementary Note

## Description of ChIP-seq, ATAC-Seq data and SE on bovine MuSCs

**-Collection of cell samples**

- The samples each from bovine MuSCs in the proliferative (GM) and myogenic differentiation and maturation (DM) phases, a total of samples were collected for ChIP-seq and ATAC-Seq analysis.

**-High-throughput sequencing**

- The target proteins of the samples were immunoprecipitated using antibodies specific for methylation of the fourth lysine residue of histone H3 (H3K4me1) and acetylation of the 27th lysine residue of histone H3 (H3K27ac). And the genomic DNA fragments bound by the H3K4me1 and H3K27ac proteins were enriched. Next, the DNA products were subjected to high-throughput sequencing by Illumina HiSeq™2500.

**-Sequence matching**

- The raw data were cleaned to obtain clean data. Sequence matching software Bowtie2 was used to compare the ChIP-seq clean data with the reference genome respectively. And the comparison result file (Binary Alignment/Map format, BAM) was obtained for the subsequent genomic region enrichment process. The reference genome selected for this method was bosTau 9 (Ensembl version: Bos_taurus.ARS-UCD1.2.101).

**-Annotation of binding peaks**

- Analysis of reads-enriched genomic regions using an H3K4me1, H3K27ac double-tagging method. Firstly, the reads-enriched genomic regions were analyzed using MACS2 software for sequencing data separately (confidence parameter: p value < 0.05) in order to obtain the H3K4me1 and H3K27ac-enriched regions, which were outputted as a Binding Peak Annotation file (Browser Extensible Data, BED). Secondly, the obtained BED file of H3K4me1 would be used for subsequent enhancer-enriched peak analysis together with the BAM file of H3K27ac obtained in step (Sequence matching).

**-Identification of SEs**

- SEs identification analyses were performed using the RANK ORDERING OF SUPER-ENHANCERS (ROSE) programming tool. Firstly, the BED file of H3K4me1 was used as the GFF (General Feature Format, GFF) file for ROSE input and then the BAM file of H3K27ac was used as the BAM file for ROSE input. Secondly, after inputting the two files, the ROSE algorithm would first stitch the H3K4me1 binding peaks in the range of 12.5 kb to obtain the constitutive enhancers. Finally, the signal values (0~100) within each constitutive enhancer were calculated from the H3K27ac BAM file. Then, the SEs were obtained by sorting the constitutive enhancers based on the H3K27ac signal values.

**-Visualization of SEs**

- The deep tools programming tool were used to further visualize the SEs data, selecting scale-region and reference-point modes to map the cellular SEs signal abundance patterns and their genetic maps. To visualize ChIP-Seq, ATAC-Seq data in IGV, a TDF file converted from a BAM file is used, which can be loaded directly into IGV to obtain track visualization information of ChIP-Seq for each sample.

**-Annotation of SEs**

- SEs vertices were annotated according to the distance of their location from the transcription start site of their associated genes. The distance of the SEs vertices from the transcription start site of the associated genes was less than 1 kb, it was annotated as a promoter (<1 kb). And the distance was more than 3 kb, it was annotated as a distal intergenic region. Meanwhile, genes within 1 Mb distance from the genome of SEs were referred to as SE-regulated genes, while circRNAs derived from SE-target genes were SE-regulated circRNAs.

## Description of TPM1_SE conditional knockout mice model

Firstly, flox heterozygous mice (flox/+) containing two loxP sites at both ends of the enhancer mCRE_26 were obtained by CRISPR-Cas9 knock-in. Then, flox heterozygous mice were crossed with Myf5-Cre mice to obtain heterozygous mice (flox/+, Myf5-Cre). Finally, the heterozygous mice (flox/+, Myf5-Cre) were crossed with flox (flox/+) to obtain pure mice (flox/flox, Myf5-Cre). Mice genotypes were identified by PCR, and mice were screened by PCR analysis of tail DNA, using the primer pairs F1:R1, F2:R2, F3:R3 which generates 546bp, 400bp, and 350bp products from the flox, WT and Cre alleles, respectively. Therefore, by genotyping, we would determine that No. 6, 10, and 11 were knockout mice, and No. 4 was wild-type.

## Impact of the TPM1_SE on muscle development in mice

Using mCRE_26^flox/flox^ mice as controls, we assessed the impact of enhancer mCRE_26 knockout on muscle development. In this study, we monitored phenotypic changes in body size, body weight, and external appearance in mice. Meanwhile, we performed overnight fasting, decapitation and execution of mice, and dissected and removed GAS, TA, SOL, and QUA muscles from the hind limbs. Next, prior to muscle injury, we analyzed TA muscles for weight to length (mg/cm) ratio, H&E staining, myofiber density and area ratio. Moreover, to explore the effects of enhancer mCRE_26 knockout on the maintenance of MuSCs pool and depletion after multiple regenerations, we evaluated muscle injury regeneration by injecting mice with two and three injections of CTX, respectively. Afterwards, we fasted and executed the mice, and dissected and removed the TA muscles. Meanwhile, we performed gene (*Pax7*, *MyOD1*, *MyOG*, and *MyHC*) expression detection and immunofluorescence staining analysis on TA muscles.

## Impact of TPM1_SE on myogenic differentiation

In order to further explore the functional similarities and differences between bovine and murine TPM1_SE, we performed loss-of-function experiments using CRISPR-Cas9 tools with enhancers bCRE_9 and mCRE_26 as typical components. First of all, we respectively designed and constructed knockout vectors for enhancer bCRE_9 and mCRE_26, and screened for gRNAs with high knockout efficiency. Knockout of mCRE_26, bCRE_9 inhibited the mRNA expression levels of *MyOD1*, *MyOG*, and *MyHC*. In conclusion, knockout of enhancer bCRE_9 and mCRE_26 inhibited the myogenic differentiation.

## Identification of CircTPM1

To further SEs and related circRNAs, we performed joint analysis of ChIP-seq as well as circRNA-seq data from bovine MuSCs. We sent the amplification product of CircTPM1 to Sanger sequencing and confirmed that CircTPM1 was generated from the Tropomyosin 1 (*TPM1*) gene. Next, we used cDNA and genomic DNA from MuSCs to design convergent primers to amplify *TPM1* mRNA and divergent primers to amplify CircTPM1. The results showed that CircTPM1 could be amplified in cDNA but not in genomic DNA. In addition, CircTPM1 was also resistant to digestion with RNase R. CircTPM1 was mainly expressed in bovine dorsal longest muscle and cardiac muscle.

## Validation of TPM1_SE, *TPM1* locus, and *TEAD4*

Based on the motif enrichment analysis of TPM1_SE positional information by bioinformatics software suites such as Homer, MEME-ChIP and IGV, we performed mapping association prediction using bovine muscle cell ATAC-seq data. The results indicated that TPM1_SE comprised a total of 26 components, of which 18 components, including E1, E2, E5, E6, E7, **bCRE_9**, E10, E11, E12, E13, E15, E16, E17, E18, E21, E22, E23, and E25, were associated with possible binding sites on the genome for transcription factors such as *TEAD4*, *Pitx1*, *Elk4*, *Gata1*, *Gfli1b*, *HIF1A*, *MYB*, and *Spib.* After ChIP/3C-qPCR analyses, we successfully verified the chromatin interactions between bovine TPM1_SE, *TPM1* promoter, and *TEAD4***.**

We analyzed the TPM1_SE region of murine skeletal muscle using UCSC genome browser(<https://genomeasia.ucsc.edu/cgibin/hgGateway?hgsid=759943672_M2XtgIjJnaOjv3rolVnM3ghMIaat>) and found that the bCRE_9 is homologous to the mCRE_26. In addition, we successfully verified the chromatin interactions between murine TPM1_SE, *TPM1* promoter, and *TEAD4* by ChIP/3C-qPCR.

# Supplementary Figures


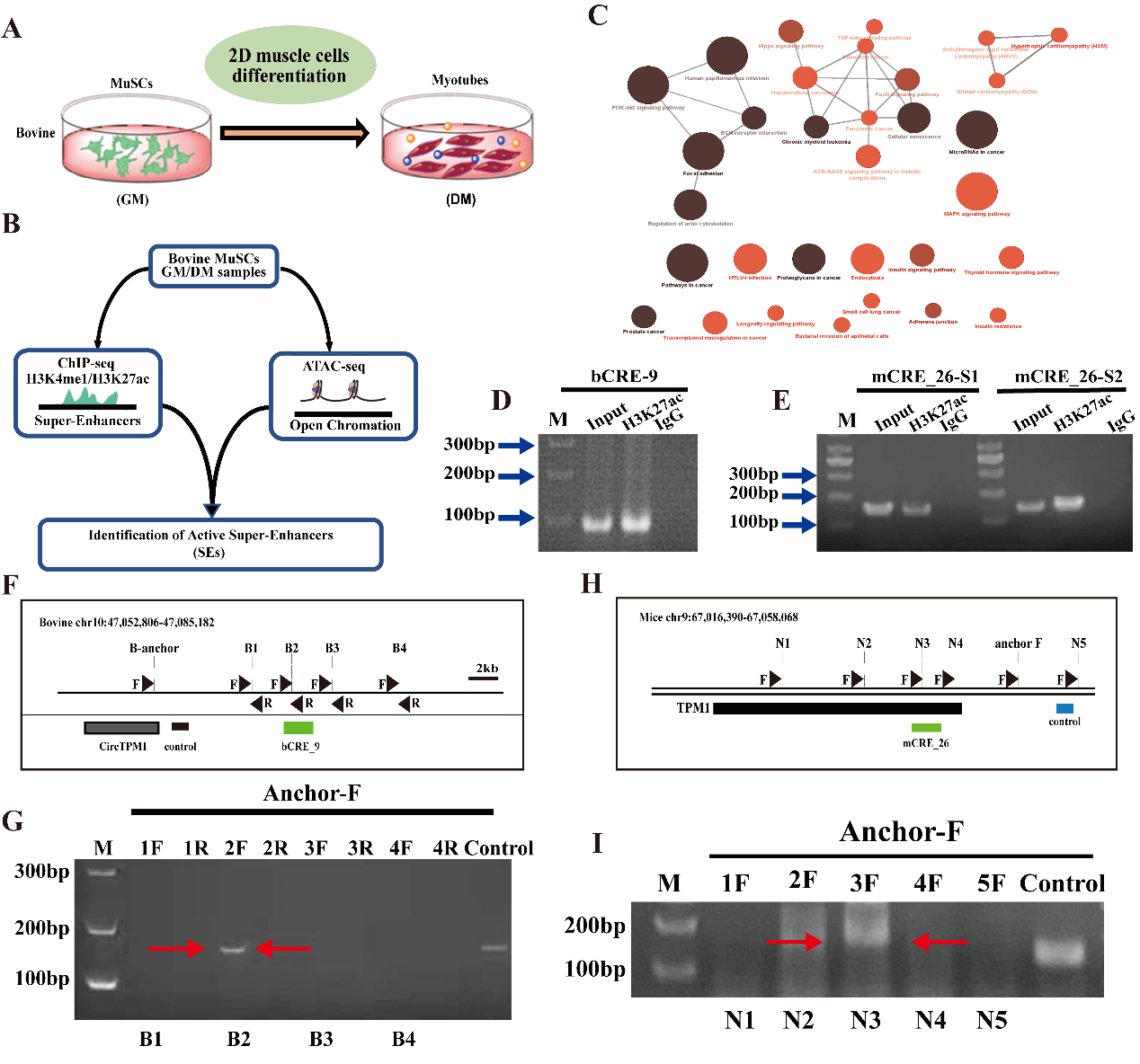


**Figure S1. Identification and functional analysis of TPM1_SE.**

**(A)** Trajectory patterns of phenotypic evolution of bovine MuSCs. **(B)** Strategies for the analysis of ChIP-seq and ATAC-seq. **(C)** KEGG pathway enrichment analysis of SE-associated genes. **(D and E)** H3K27ac activity of enhancers bCRE_9 (**D**) and mCRE_26 (**E**) were detected by ChIP-qPCR and agar gel electrophoresis. **(F-I)** Assay sites were designed to validate interactions of bCRE_9 (**F, G**) and mCRE_26 (**H, I**) with the TPM1 promoter. Interactions were detected using 3C-qPCR and agar gel electrophoresis.


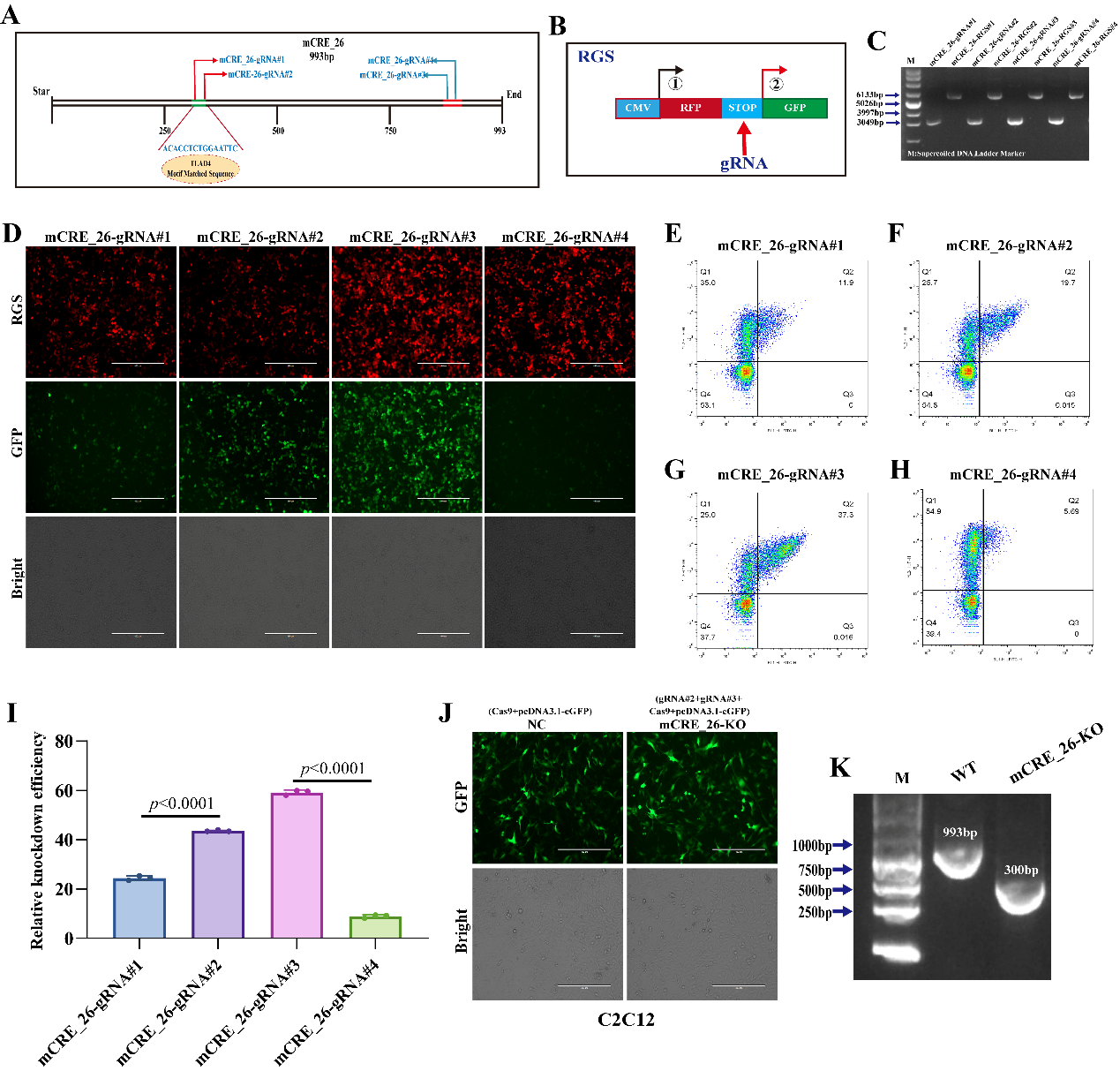


**Figure S2. Design and screening of gRNAs for efficient knockout of** **mCRE_26.**

**(A)** Design of gRNA sites in the CRISPR/Cas9 system of knockout enhancer mCRE_26 and its vector construction. GRNA site design for enhancer mCRE_26. **(B)** Initiation cleavage mode of gRNA. **(C)** Construction of a knockout combinatorial vector（gRNA + RGS）for the mCRE_26 CRISPR/Cas9 system. (**D)** Detection of different gRNA cleavage efficiencies of mCRE_26. The expression of red fluorescent protein and green fluorescent protein on 293T cells was analyzed by fluorescence microscopy. **(E-I)** Detection of different gRNA cleavage efficiencies for knockout mCRE_26. Flow cytometry was used to analyze the cleavage efficiency of mCRE_26-gRNA#1, mCRE_26-gRNA#2, mCRE_26-gRNA#3, and mCRE_26-gRNA#4. **(J and K)** Detection of mCRE_26 knockout fragments. The effects of mCRE_26-gRNA#2, mCRE_26-gRNA#3, Cas9 and pcDNA3.1-eGFP plasmids were co-electromorphized to C2C12 myoblasts by fluorescence microscopy(**J**). Enhanced mCRE_26 deletion fragments were detected by RT-PCR and agar gel electrophoresis(**K**). Bar graphs show mean ± SEM of three individuals. Scale bars, 400 µm. Data are presented as mean ± SEM (n = 3).


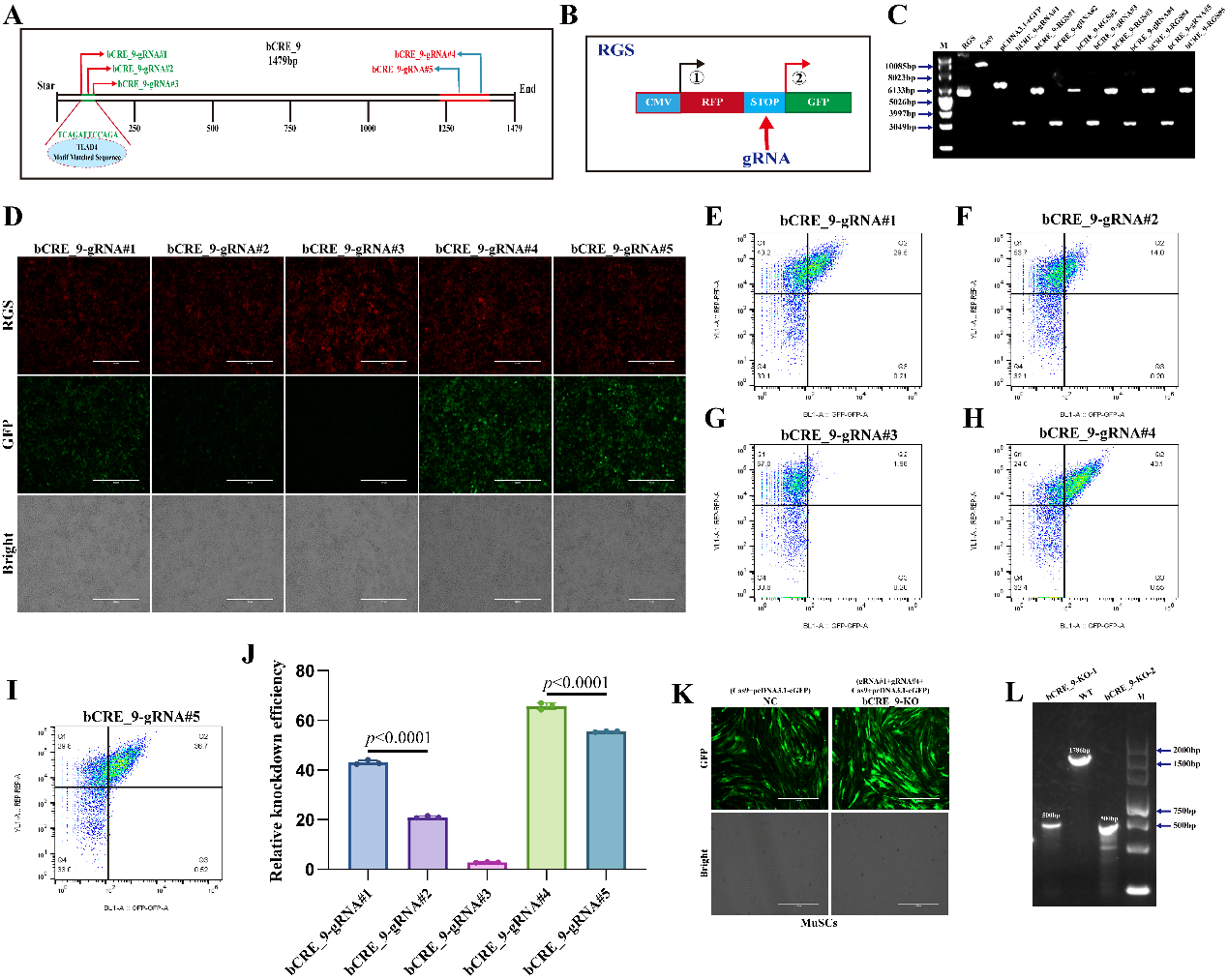


**Figure S3. Design and screening of gRNAs for efficient knockout of bCRE_9.**

**(A)** Design of gRNA sites in the CRISPR/Cas9 system of knockout enhancer bCRE_9 and its vector construction. GRNA site design for enhancer bCRE_9. **(B)** Initiation cleavage mode of gRNA. **(C)** Construction of a knockout combinatorial vector（gRNA + RGS）for the enhancer bCRE_9 CRISPR/Cas9 system. **(D)** Detection of different gRNA cleavage efficiencies of the enhancer bCRE_9. The expression of red fluorescent protein and green fluorescent protein on 293T cells was analyzed by fluorescence microscopy. **(E-J)** Detection of different gRNA cleavage efficiencies for knockout enhancer E9. Flow cytometry was used to analyze the cleavage efficiency of bCRE_9-gRNA#1, bCRE_9-gRNA#2, bCRE_9-gRNA#3, bCRE_9-gRNA#4 and bCRE_9-gRNA#5. **(K and L)** Detection of enhancer bCRE_9 knockout fragments. The effects of bCRE_9-gRNA#1, bCRE_9-gRNA#4, Cas9 and pcDNA3.1-eGFP plasmids were co-electromorphized to bovine MuSCs by fluorescence microscopy **(K)**. Enhanced bCRE_9 deletion fragments were detected by RT-PCR and agar gel electrophoresis **(L)**. Bar graphs show mean ± SEM of three individuals. Scale bars, 400 µm. Data are presented as mean ± SEM (n = 3).


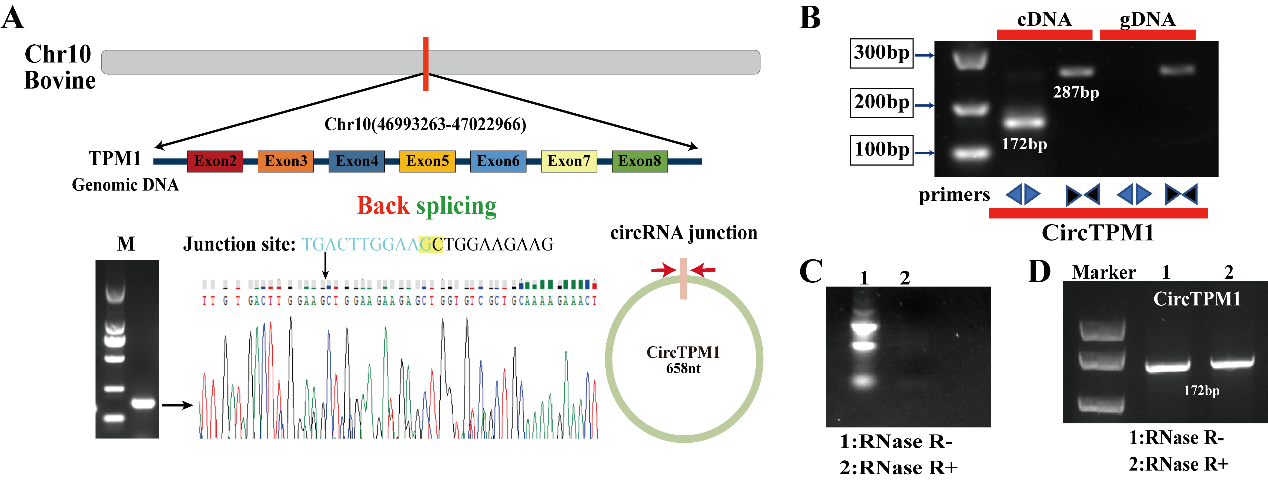


**Figure S4. Identification and functional analysis of CircTPM1.**

**(A)** CircTPM1 is formed by the cyclization of exons 2-8 of the *TPM1* locus shear, with a length of 658 nt. The red arrow indicates the splice site that follows, and the presence of the splice site was verified by Sanger sequencing. **(B)** Design of divergent primer and convergent primer to identify the loop structure of CircTPM1 by RT-PCR. **(C and D)** Total RNA from bovine MuSCs were treated with RNase R reagent **(C)**, CircTPM1 expression was detected by RT-PCR and agar gel electrophoresis **(D)**.


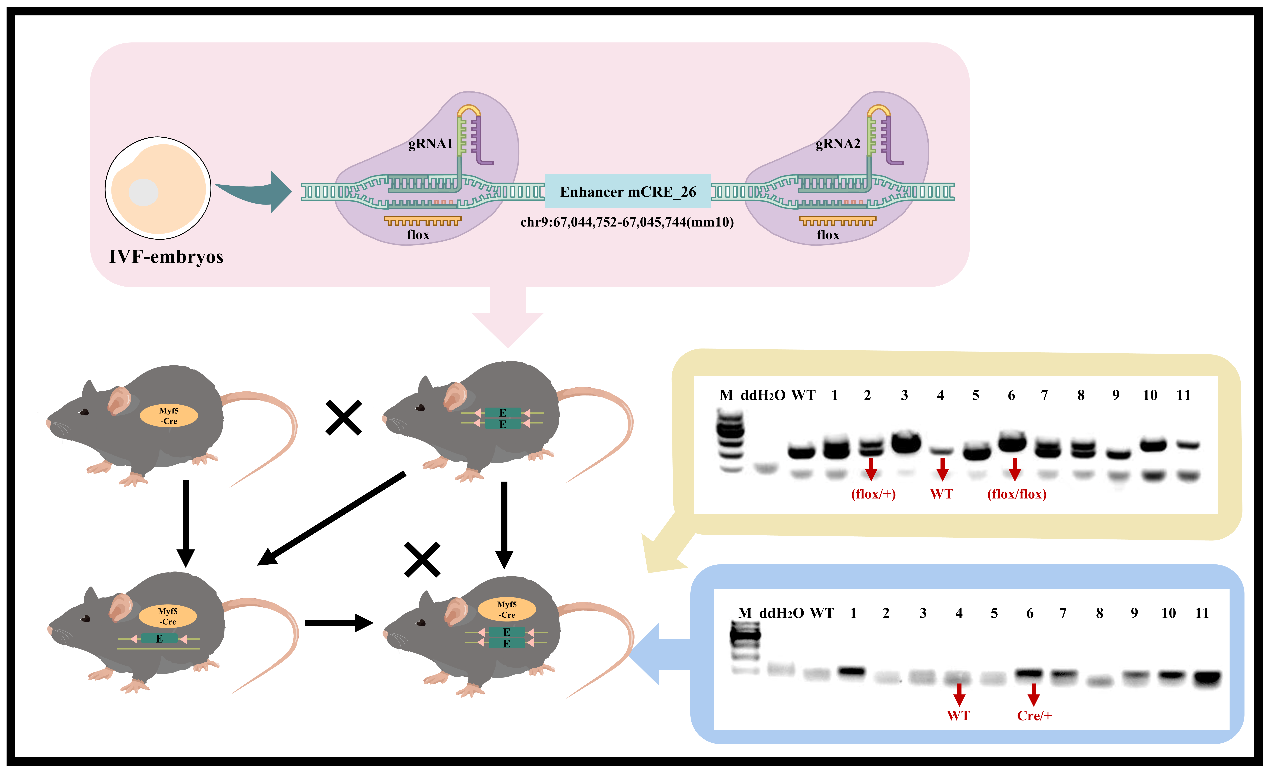


**Figure S5. Strategies for the preparation of mCRE_26 conditional knockout mice.**

To create a enhancer mCRE_26 conditional knockout mice model by CRISPR/Cas9-mediated genome engineering（named : mCRE_26^cKO^）. This research utilized IVF to cross Myf5-Cre mice with mCRE_26^flox/flox^ mice to obtain mCRE_26^cKO^ pureblooded mice (genotype: mCRE_26^flox/flox+Myf5-Cre^).

**
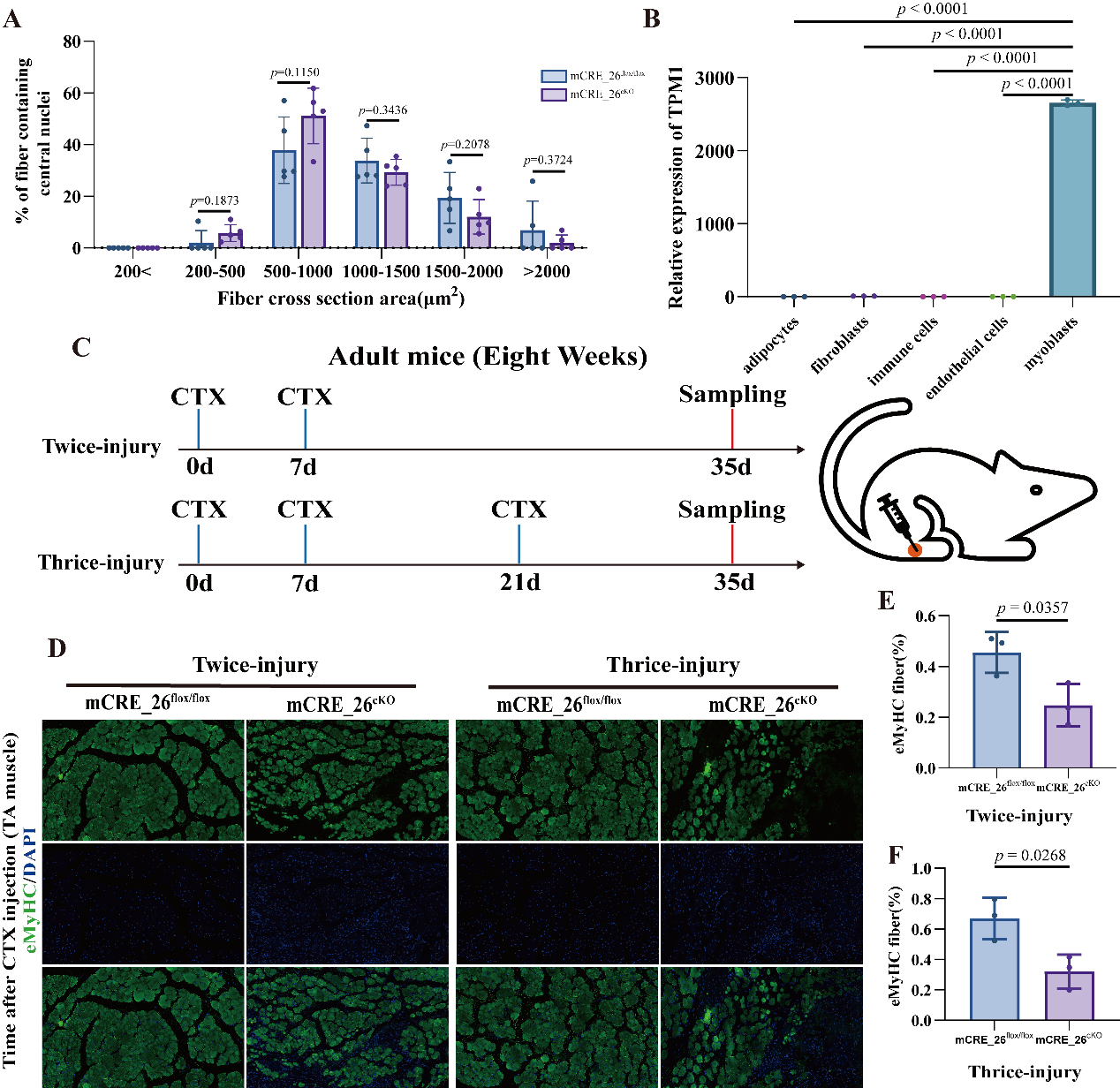
**

**Figure S6.** **Phenotypic characterization of mCRE_26^cKO^ mice and assessment of multiple muscle injury models.**

**(A)** Quantification of the percentage of regenerated muscle fibers in the indicated cross-sectional area of undamaged TA muscle by ImageJ and Image-Pro Plus for 500 nucleus-containing muscle fibers in mCRE_26^flox/flox^ and mCRE_26^cKO^ mice. (**B**) Analysis of TPM1 Expression Across Cell Types in Mice. **(C)** Multiple Injury TA Muscles Testing Routes. In the Twice-injury experiment, mice received intramuscular injections of 50 µL CTX on Day 0 and Day 7, and muscle tissues were harvested on Day 28 following the final injection for evaluation of regeneration. In the Thrice-injury experiment, CTX were administered on Day 0, Day 7, and Day 21, with samples collected on Day 14 after the last injection for assessment of muscle regrowth and histological analysis. **(D-F)** Inhibition of muscle development and significant slowing of repair after multiple muscle injuries in mCRE_26^cKO^ mice. The number of eMyHC (green, differentiated myofibers) were analyzed by immunofluorescence (DAPI, blue). Data are presented as mean ± SEM (n = 5). Scale bars, 20 µm.

**
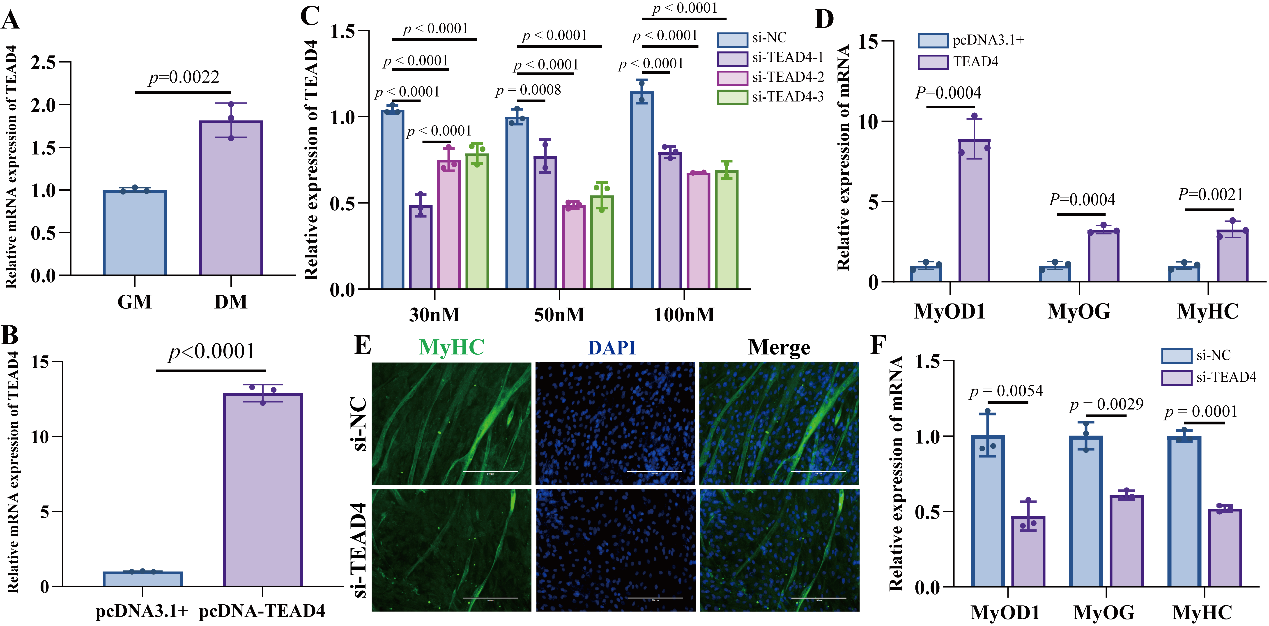
**

**Figure S7. Expression and Functional Analysis of *TEAD4*.**

**(A)** The mRNA expression level of *TEAD4* in the proliferation and myogenic differentiation of bovine MuSCs was detected by RT-qPCR. (**B)** The overexpression efficiency of *TEAD4* was detected by RT-qPCR in bovine MuSCs. **(C)** Detection of the interference efficiency of different si-TEAD4 24 h after transfection. RT-qPCR results showed that si-TEAD4-1 exhibited the highest efficiency in inhibiting TEAD4 expression, achieving approximately 50% inhibition at a concentration of 30 nM. **(D)** Overexpression of *TEAD4* promotes the expression of muscle markers in bovine MuSCs. Detection of mRNA expression levels of *MyOD1*, *MyOG*, and *MyHC* by RT-qPCR. **(E)** Immunofluorescence revealed that *TEAD4* interference inhibited myogenic differentiation, evidenced by decreased MyHC-positive myotubes (DAPI, blue). **(F)** Interfering with *TEAD4* inhibits the expression of muscle markers in bovine MuSCs. Detection of mRNA expression levels of *MyOD1*, *MyOG*, and *MyHC* by RT-qPCR. Data are presented as mean ± SEM (n = 3).

**
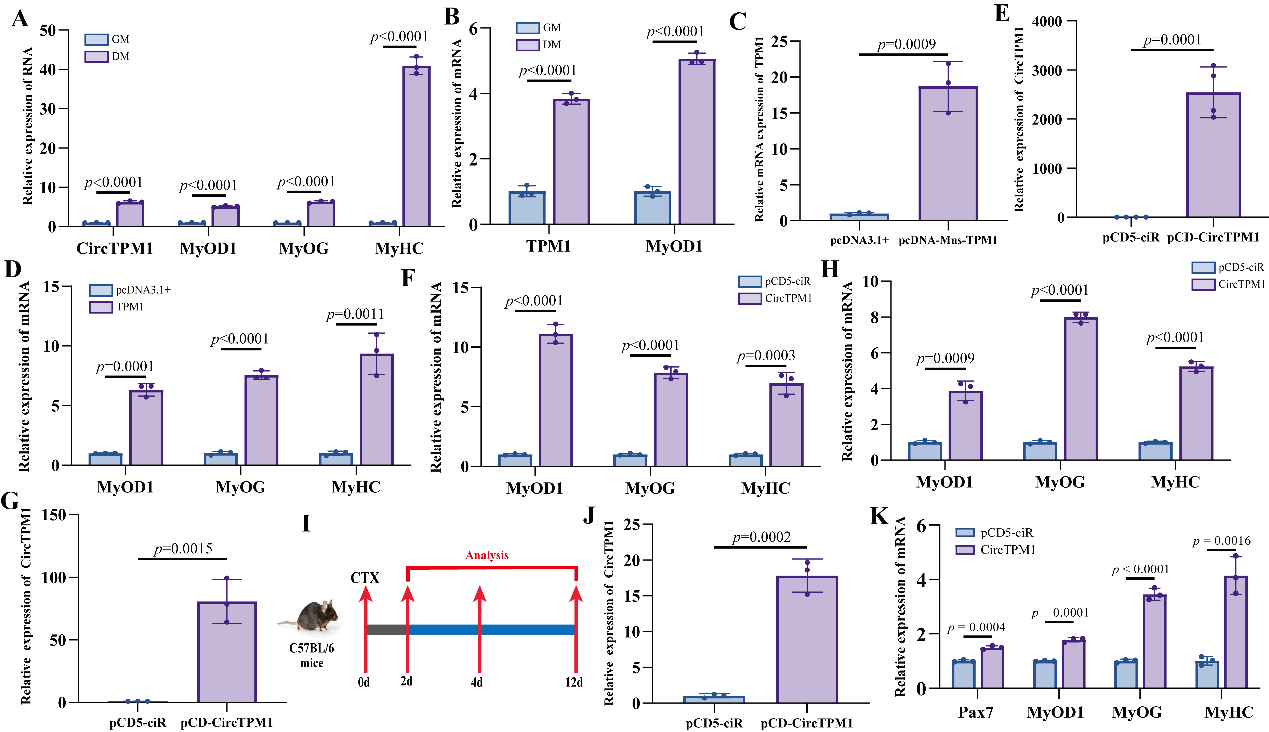
**

**Figure S8. Expression and Functional Analysis of murine *TPM1* and bovine CircTPM1.**

**(A and B)** The RNA expression levels of bovine CircTPM1 (**A**), murine *TPM1*(**B**), *MyOD1*, *MyOG*, and *MyHC* in the proliferation and myogenic differentiation of myoblasts was detected by RT-qPCR. **(C)** The overexpression efficiency of murine *TPM1* was detected by RT-qPCR. **(D)** Overexpression of murine *TPM1* promotes the expression of muscle markers in C2C12 myoblasts. Detection of mRNA expression levels of *MyOD1*, *MyOG*, and *MyHC* by RT-qPCR. **(E)** The overexpression efficiency of bovine CircTPM1 was detected by RT-qPCR. **(F)** Overexpression of CircTPM1 promotes the expression of muscle markers in bovine MuSCs. Detection of mRNA expression levels of *MyOD1*, *MyOG*, and *MyHC* by RT-qPCR. **(G)** Detection of CircTPM1 expression efficiency in C2C12 myoblasts by RT-qPCR. **(H)** Overexpression of CircTPM1 promotes the expression of muscle markers in C2C12 myoblasts. Detection of mRNA expression levels of *MyOD1*, *MyOG*, and *MyHC* by RT-qPCR. **(I)** A schematic outlines the experimental timeline: mice were administered either the control vector (pCD5-ciR) or the CircTPM1 overexpression plasmid (pCD5-ciR: CircTPM1), followed by cardiotoxin (CTX) injection. Plasmids were delivered at 2 and 4 days post-CTX, and tissues were collected 8 days after the final transfection. **(J)** The expression efficiency of CircTPM1 in TA muscle was detected by RT-qPCR. (**K**) RT-qPCR revealed that CircTPM1 significantly upregulated mRNA expression of key myogenic markers: *Pax7*, *MyOD1*, *MyOG*, and *MyHC*. Data are presented as mean ± SEM (n = 3).

**
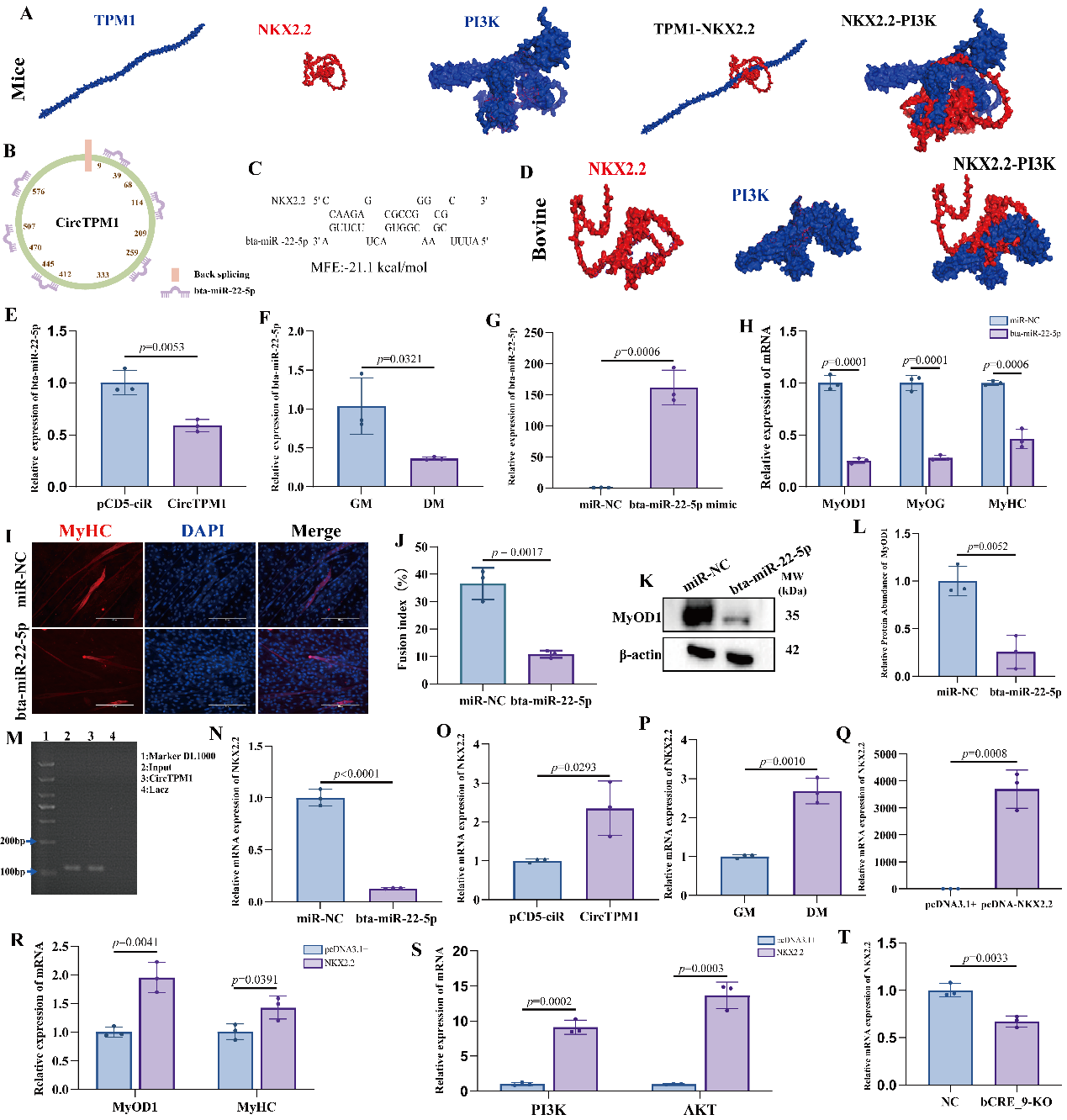
**

**Figure S9.** **Interactions among CircTPM1, bta-miR-22-5p, and NKX2.2.**

**(A)** Molecular dynamics simulations using PyMOL revealed spatial complementarity and thermodynamic stability at the predicted interaction sites between murine TPM1, NKX2.2, and PI3K proteins. **(B)** Bioinformatic analysis predicted a binding site for bta-miR-22-5p on CircTPM1. **(C)** NKX2.2 was also predicted to bind bta-miR-22-5p. **(D)** Molecular dynamics simulations further confirmed spatial and thermodynamic compatibility between bovine NKX2.2 and PI3K. **(E)** The expression level of bta-miR-22-5p after bovine MuSCs overexpressed CircTPM1 was analyzed by RT-qPCR. **(F)** The expression level of bta-miR-22-5p in the proliferation and myogenic differentiation of bovine MuSCs was analyzed by RT-qPCR. **(G)** In bovine MuSCs, the overexpression efficiency of bta-miR-22-5p was analyzed by RT-qPCR. **(H)** Overexpression of bta-miR-22-5p inhibits the expression of muscle markers. The mRNA levels of *MyOD1*, *MyOG*, and *MyHC* were detected by RT-qPCR. **(I)** The number of MyHC-positive myotubes was analyzed by immunofluorescence (DAPI, blue). **(J)** Fusion index. **(K)** Protein expression analysis of MyOD1 by WB. **(L)** Quantification of WB as shown in **K**. **(M)** The enrichment capacity of CircTPM1 to bta-miR-22-5p was analyzed by ChIRP-qPCR and agar gel electrophoresis. **(N)** The mRNA level of *NKX2.2* after bovine MuSCs overexpressed bta-miR-22-5p was analyzed by RT-qPCR. **(O)** In bovine MuSCs, the mRNA level of *NKX2.2* after overexpressed CircTPM1 was analyzed by RT-qPCR. **(P)** The mRNA level of *NKX2.2* in the proliferation and myogenic differentiation of bovine MuSCs was analyzed by RT-qPCR. **(Q)** Construction of *NKX2.2* overexpression vector and analysis of its expression efficiency in bovine MuSCs. Recombinant plasmid pcDNA-NKX2.2. And detection of the expression efficiency of NKX2.2 by RT-qPCR. **(R)** Overexpression of *NKX2.2* promotes the expression of muscle markers. The mRNA levels of *MyOD1* and *MyHC* were detected by RT-qPCR. **(S)** Overexpression of *NKX2.2* activates the PI3K/AKT signaling pathway. The mRNA levels of *PI3K* and *AKT* were detected by RT-qPCR. **(T)** Knockout of bCRE_9 inhibits the mRNA levels of *NKX2.2* in bovine MuSCs. Data are presented as mean ± SEM (n = 3).


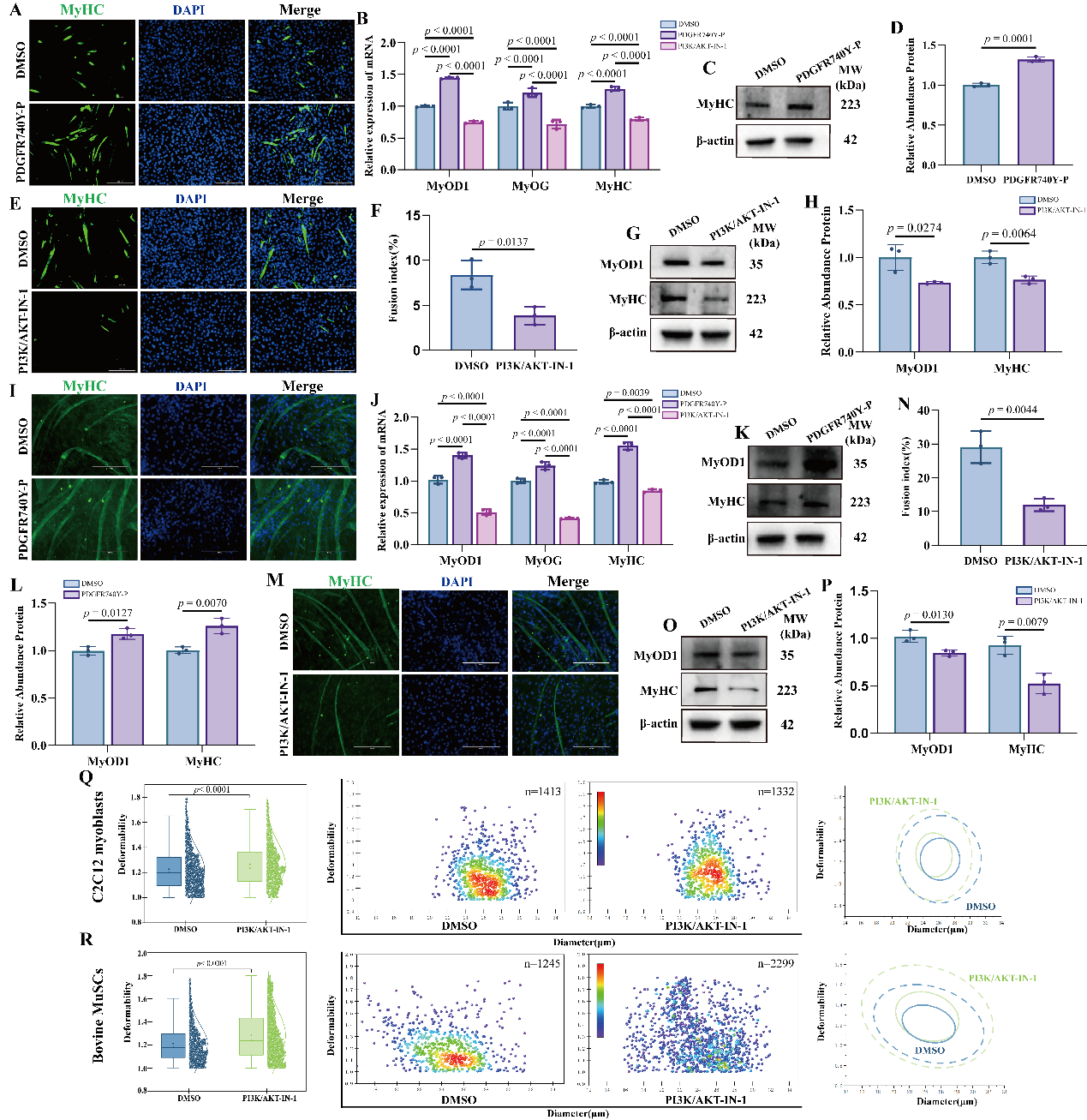


**Figure S10.** **PI3K/AKT Signaling pathway in Myogenic Differentiation and mechanotransduction.**

**(A)**Immunofluorescence revealed that activation of the PI3K/AKT signaling pathway enhanced myogenic differentiation in C2C12 myoblasts, evidenced by an increased formation of MyHC-positive myotubes (DAPI, blue). **(B)** The mRNA levels of *MyOD1*, *MyOG*, and *MyHC* were detected by RT-qPCR. (**C**) The protein levels of MyOD1 was detected by WB. (**D**) Quantification of the WB data in panel **C** validated this upregulation. **(E)** Inhibition of the PI3K/AKT signaling pathway impaired myogenic differentiation in C2C12 myoblasts, evidenced by decreased MyHC-positive myotubes (DAPI, blue). **(F)** Fusion index. (**G**) The protein levels of MyOD1 and MyHC were detected by WB. (**H**) Quantification of the WB data in panel **G** supports these observations. **(I)**Immunofluorescence revealed that activation of the PI3K/AKT signaling pathway enhanced myogenic differentiation in bovine MuSCs, evidenced by an increased formation of MyHC-positive myotubes (DAPI, blue). **(J)** The mRNA levels of *MyOD1*, *MyOG*, and *MyHC* were detected by RT-qPCR. (**K**) The protein levels of MyOD1 and MyHC were detected by WB. (**L**) Quantification of the WB data in panel **K** validated this upregulation. **(M)** Inhibition of the PI3K/AKT signaling pathway impaired myogenic differentiation in bovine MuSCs, evidenced by decreased MyHC-positive myotubes (DAPI, blue). **(N)** Fusion index. (**O**) The protein levels of MyOD1 and MyHC were detected by WB. (**P**) Quantification of the WB data in panel **O** supports these observations. (**Q** and **R**) Cell deformability in C2C12 myoblasts (**Q**) and bovine MuSCs (**R**) were measured upon inhibition of the PI3K/AKT pathway. The left panel presents a box chart illustrating the deformation values across these groups. The middle panel shows a scatter plot depicting the relationship between deformability and cell diameter. In the right panel, density contour plots are displayed, with solid and dashed lines representing the 50% and 95% density contours, respectively, for comparisons between different treatment groups. Data are from n ≥ 1000 cells per subgroup. Data are presented as mean ± SEM (n = 3).

**
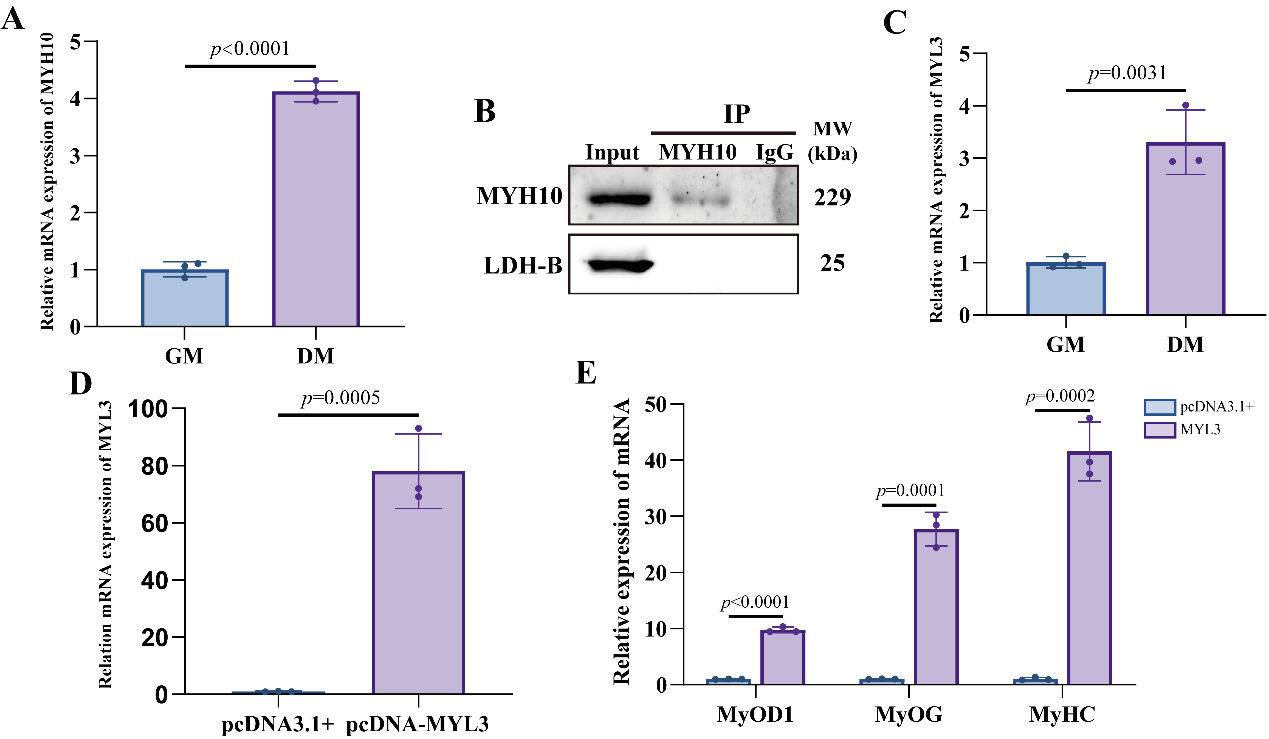
**

**Figure S11. Interactions among CircTPM1, MYH10 and MYL3.**

**(A)** The mRNA level of *MYH10* in the proliferation and myogenic differentiation of bovine MuSCs was analyzed by RT-qPCR. **(B)** Analysis of the interaction between MYH10 and LDH-B by Co-IP/WB. **(C)** The mRNA level of *MYL3* in the proliferation and myogenic differentiation of bovine MuSCs was analyzed by RT-qPCR. **(D)** Construction of *MYL3* overexpression vector and analysis of its expression efficiency in bovine MuSCs. Recombinant plasmid pcDNA-MYL3. And detection of the expression efficiency of *MYL3* by RT-qPCR. **(E)** Overexpression of *MYL3* promotes the expression of muscle markers. The mRNA levels of *MyOD1*, *MyOG*, and *MyHC* were detected by RT-qPCR. Data are presented as mean ± SEM (n = 3).

# Uncropped Western blots

**Figure 2C**


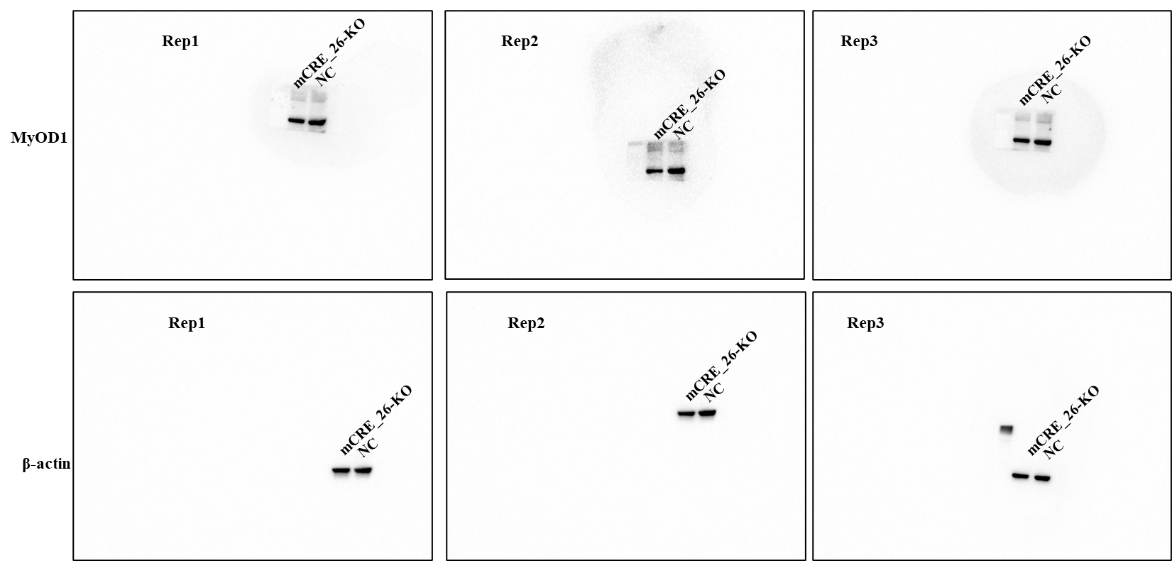


**Figure 2G**


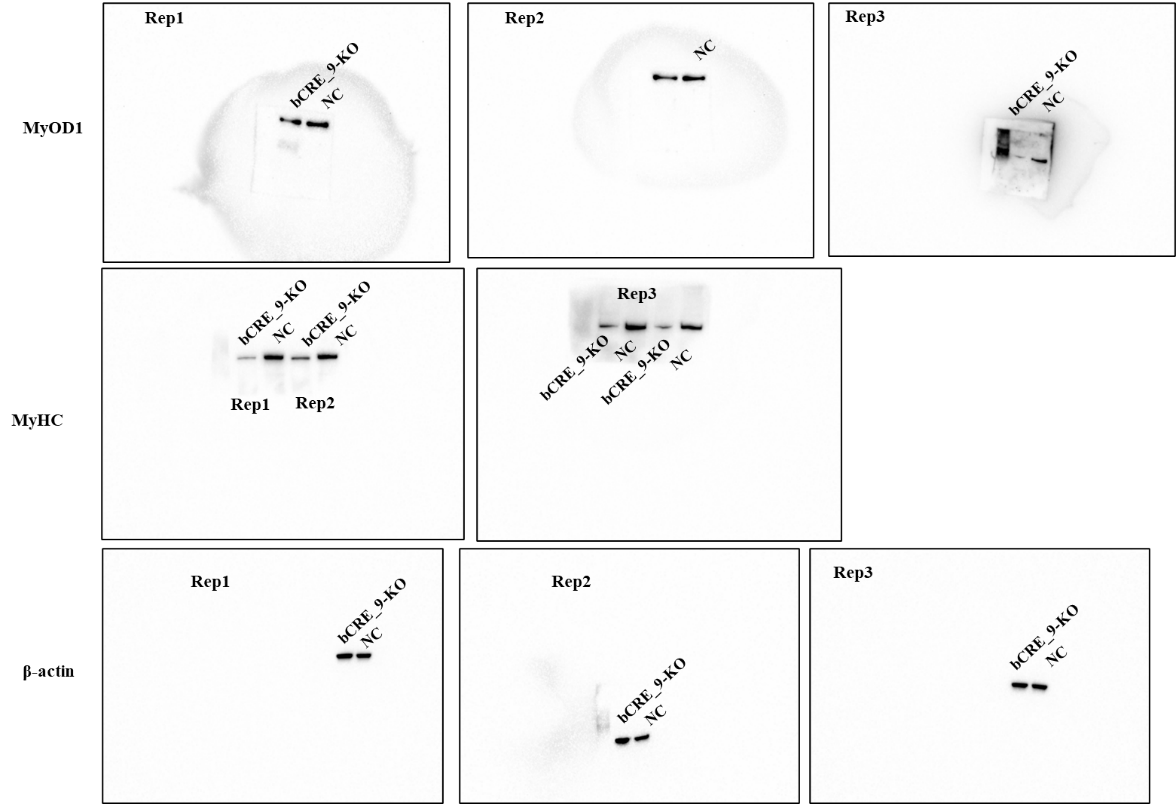


**Figure 4K**


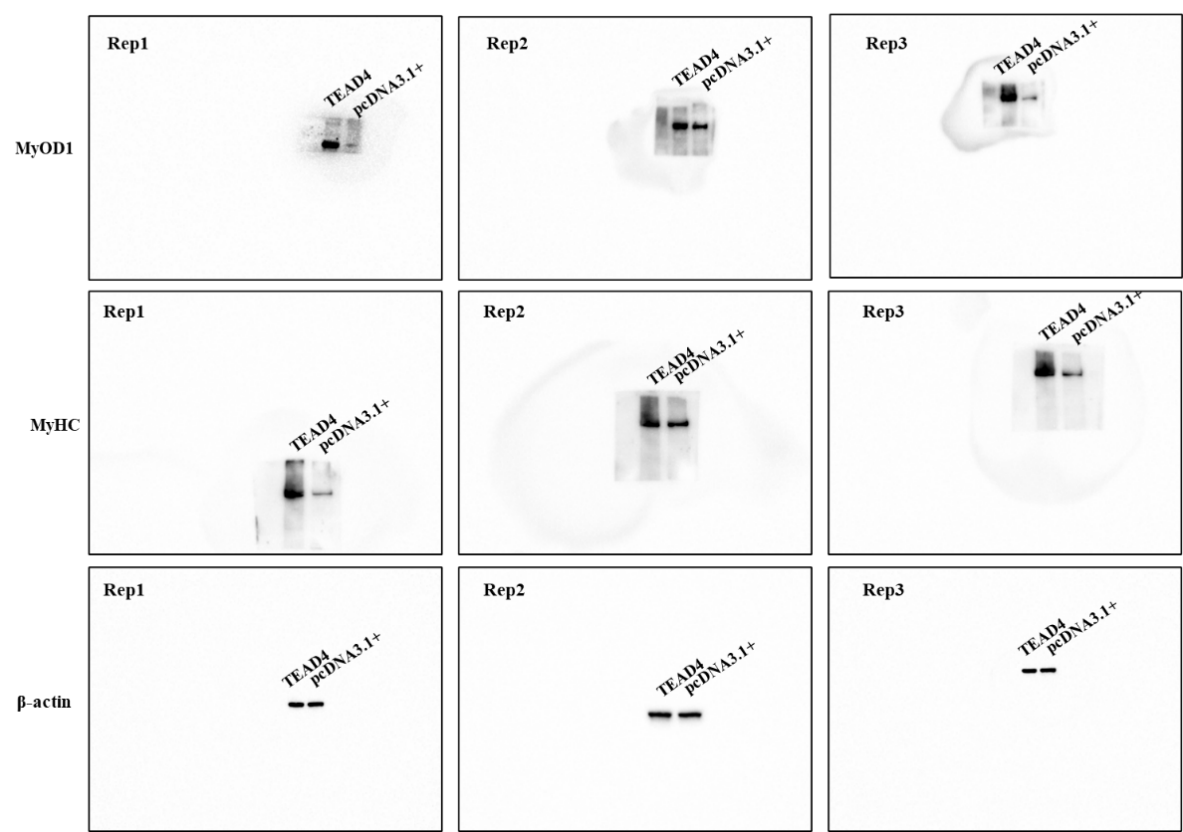

**Figure 4N**


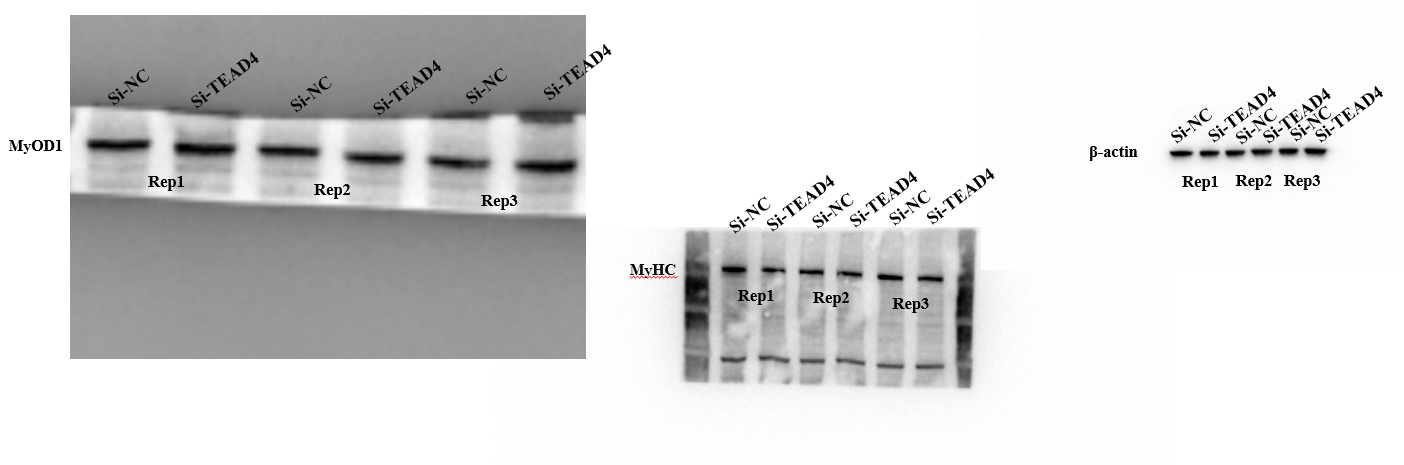


**Figure 5C**


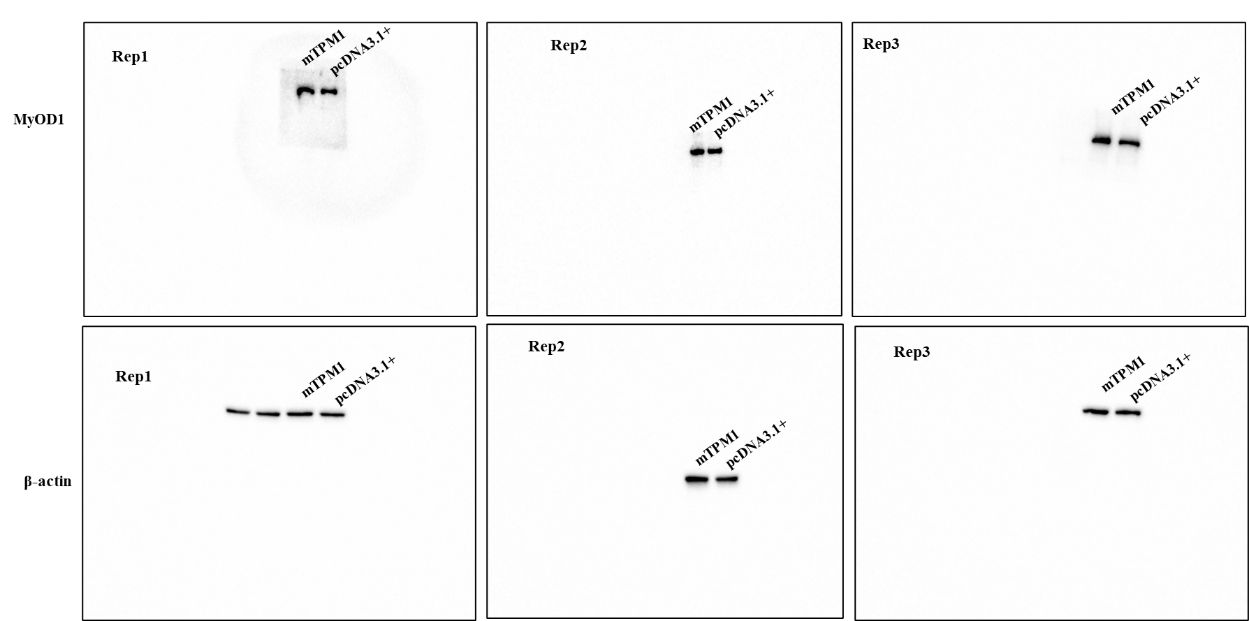


**Figure 5G**


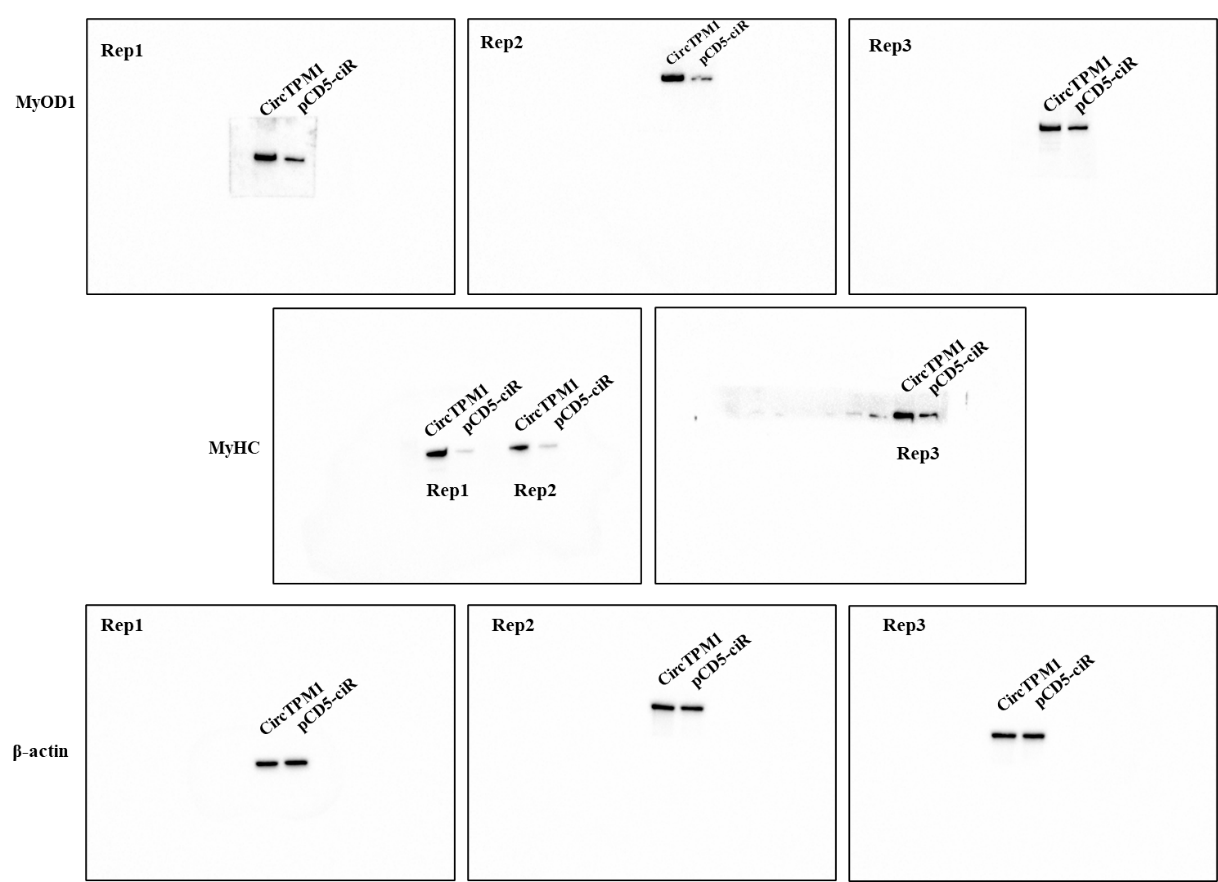


**Figure 5K**


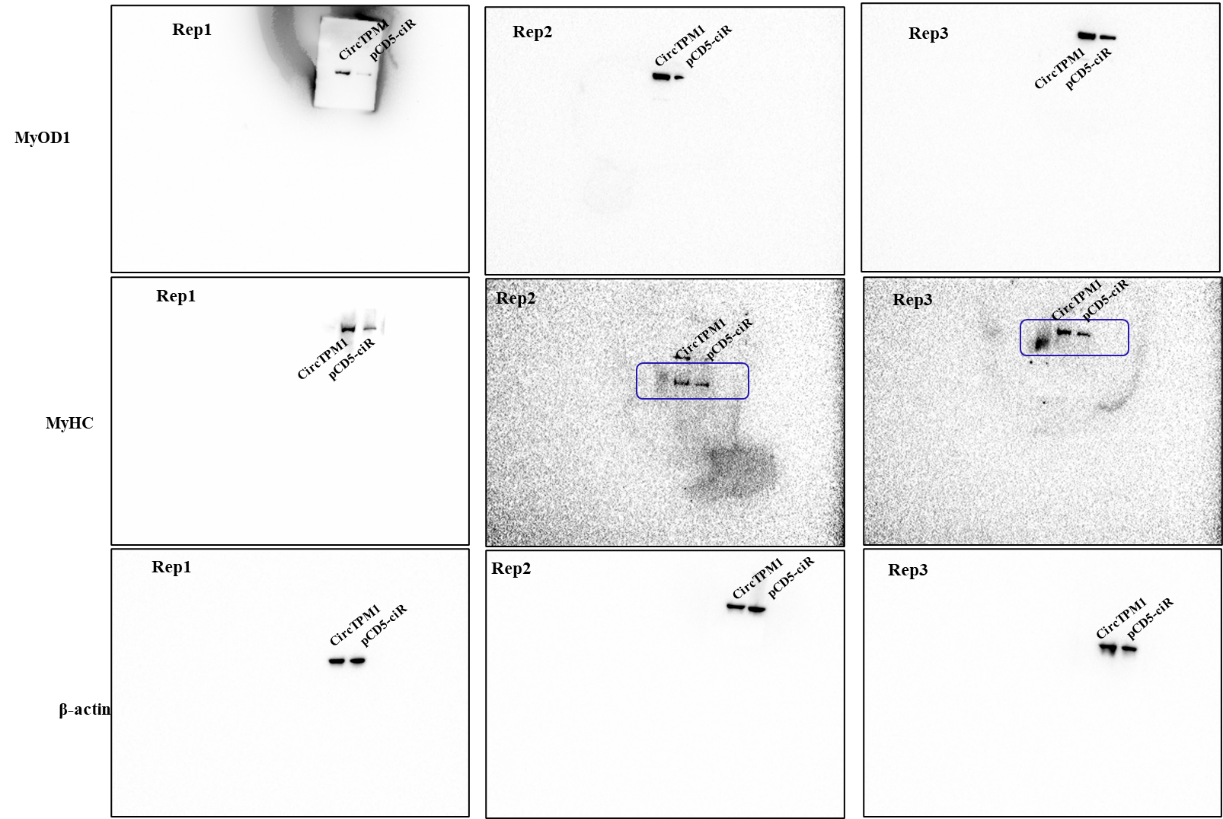


**Figure 6B**


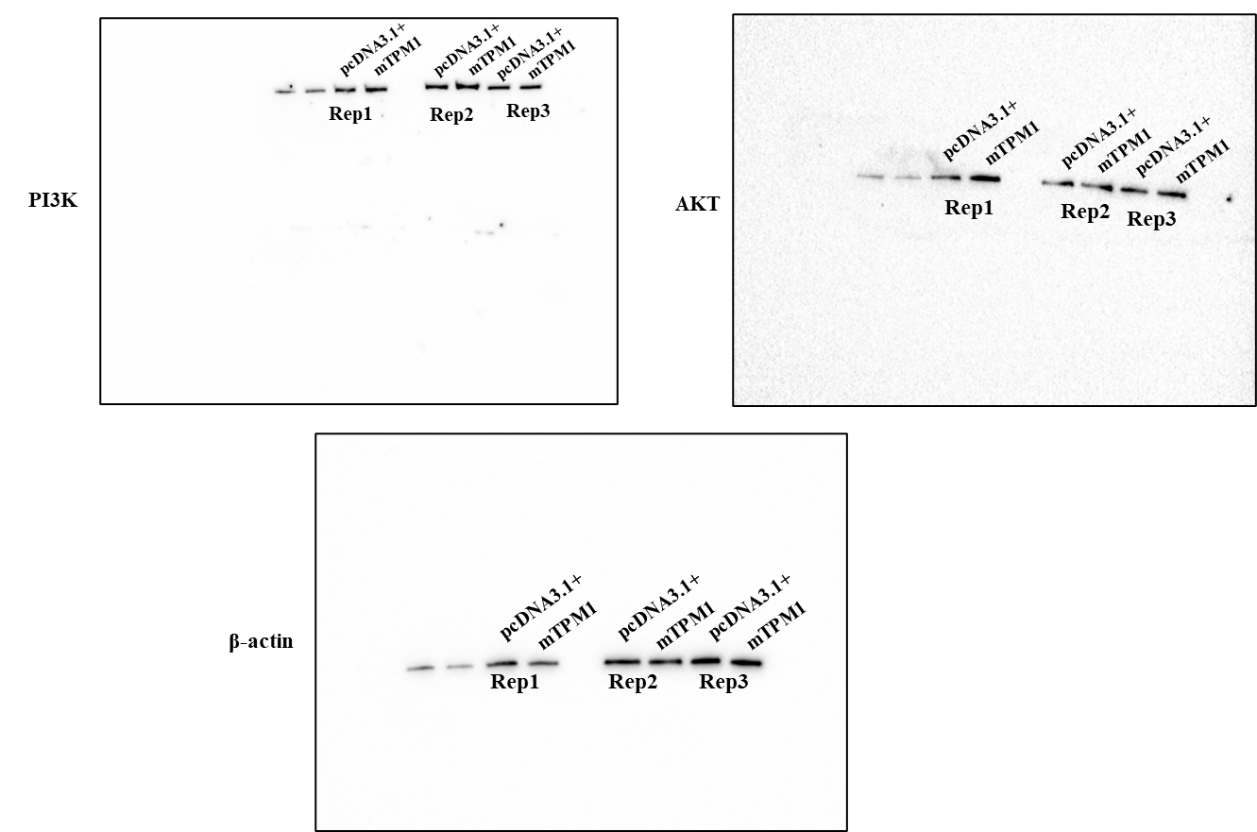


**Figure 6D**


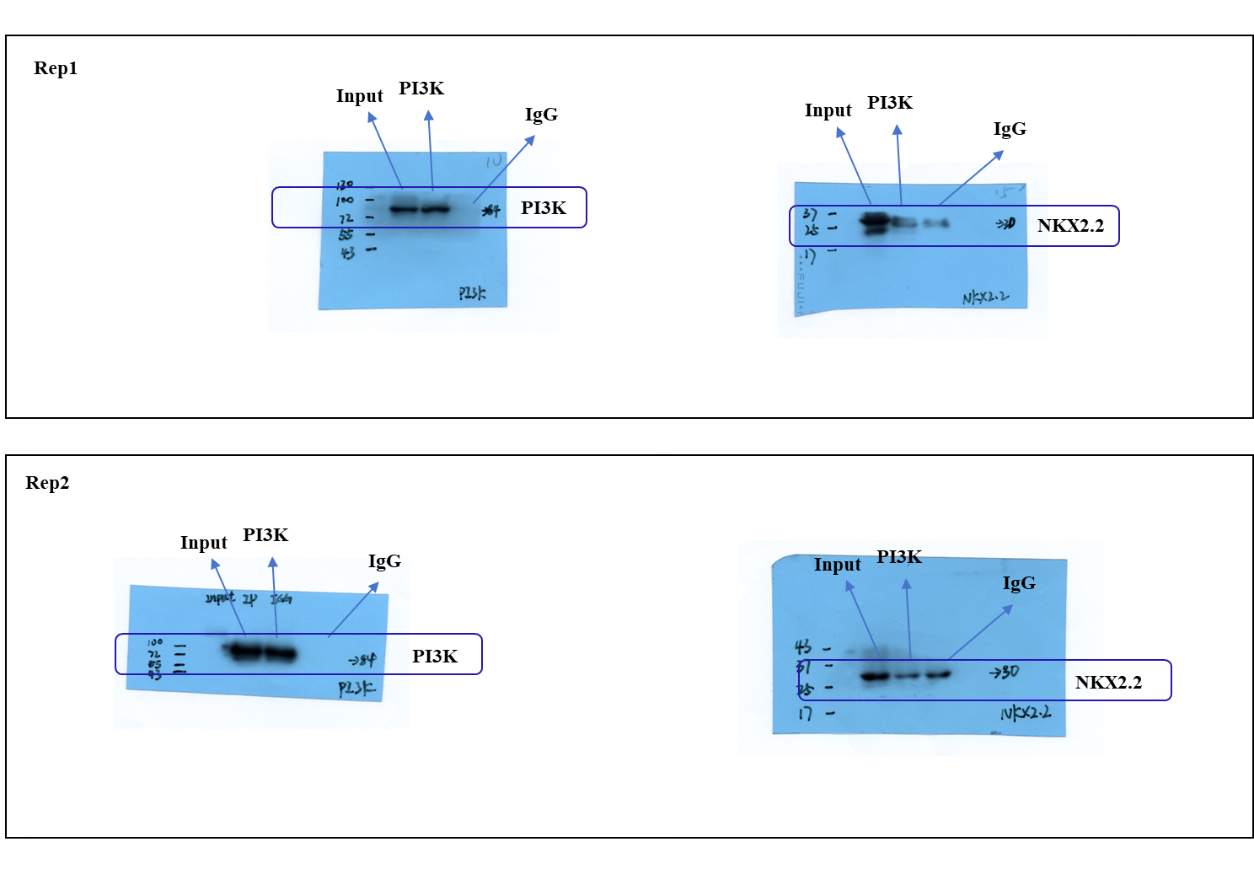


**Figure 6E**


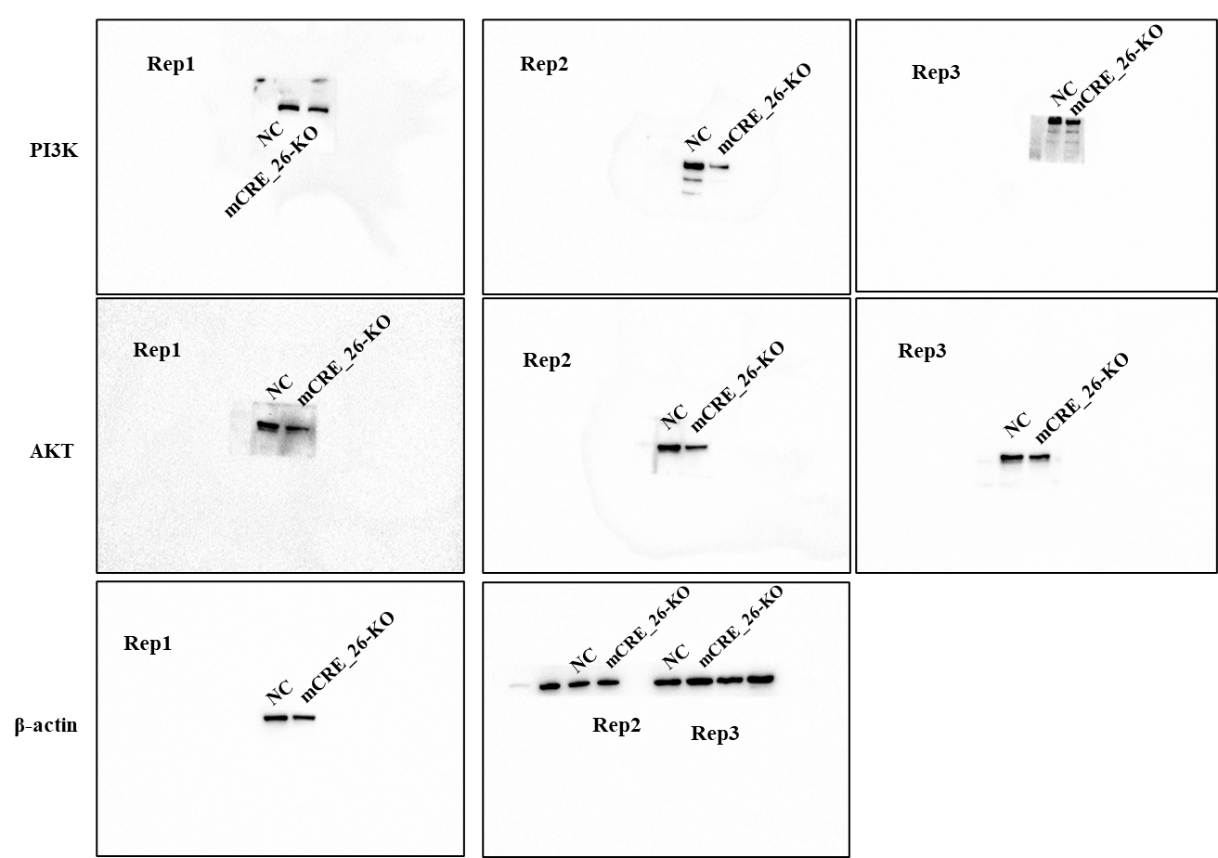


**Figure 6G**


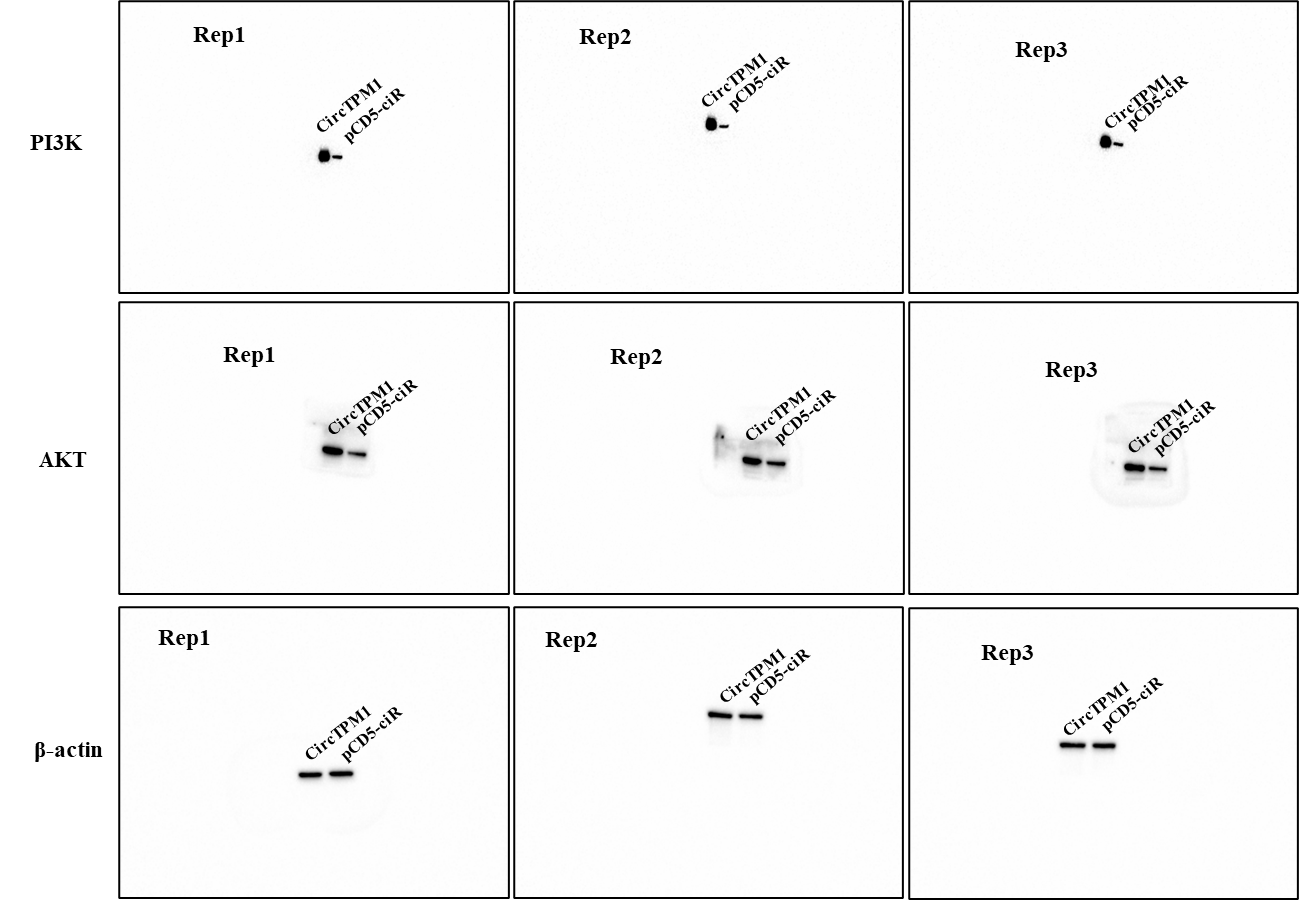


**Figure 6O**


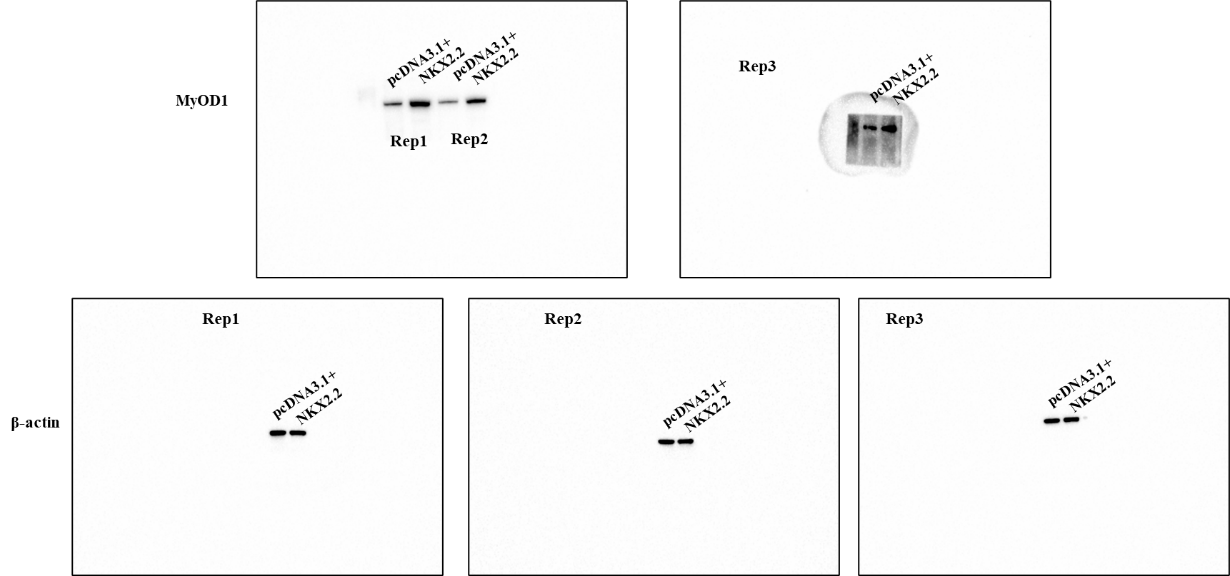


**Figure 6Q**


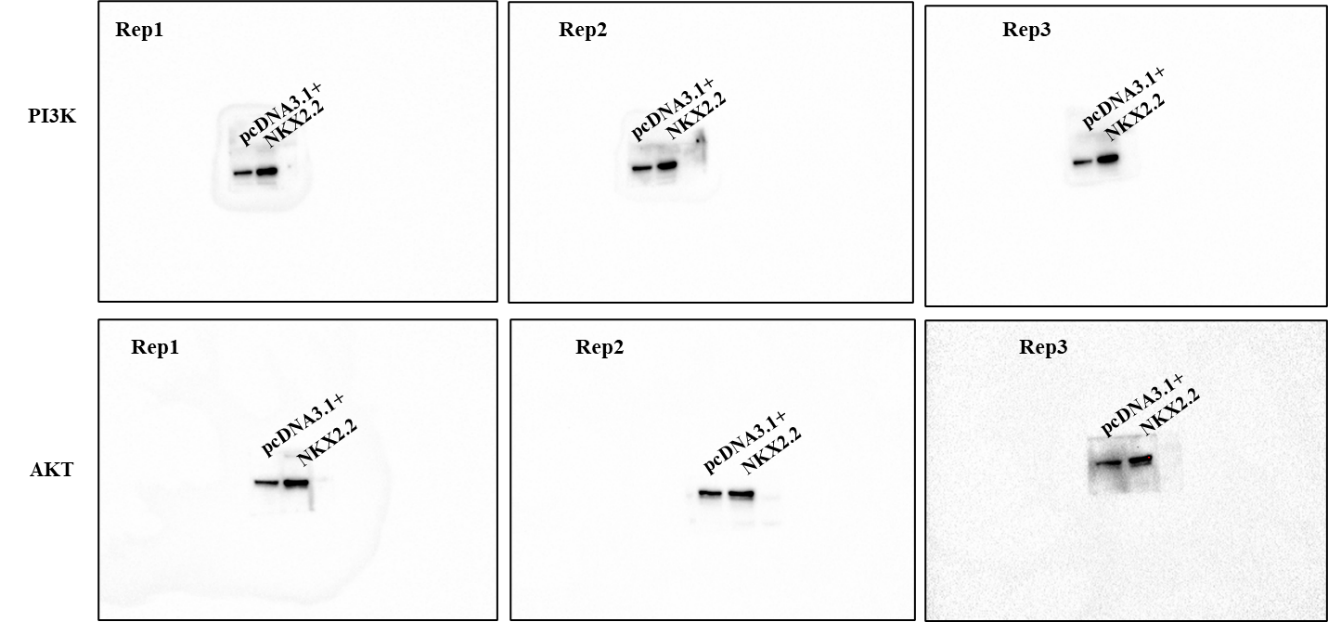


**Figure 6S**


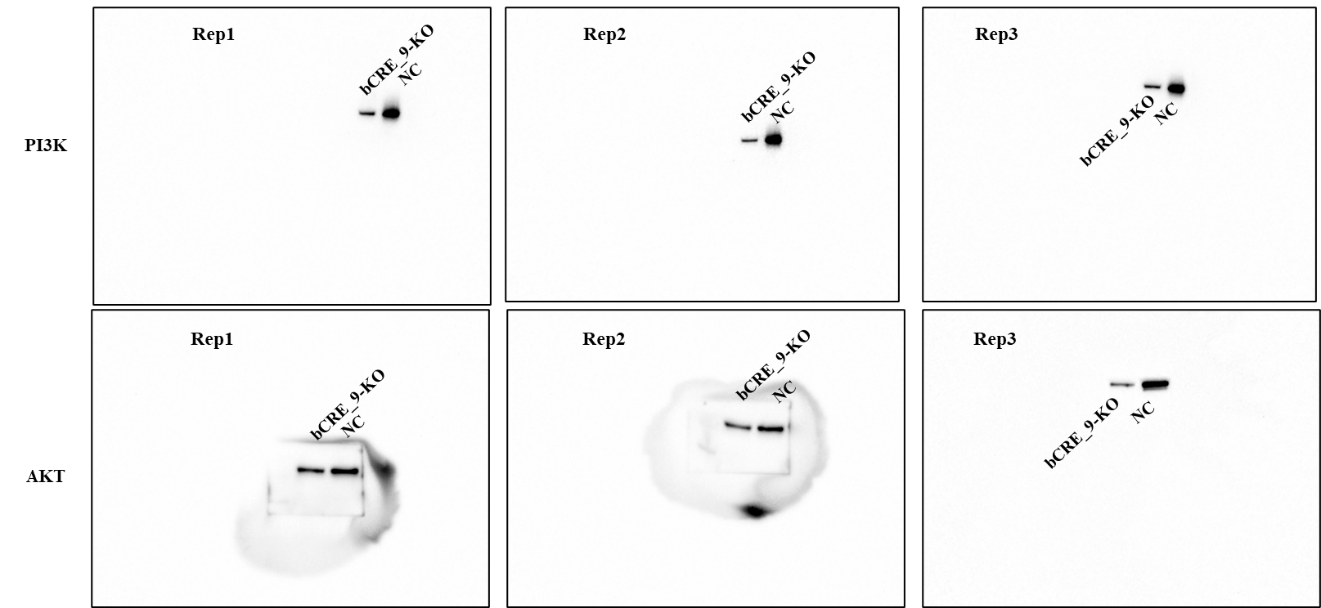


**Figure 7E**


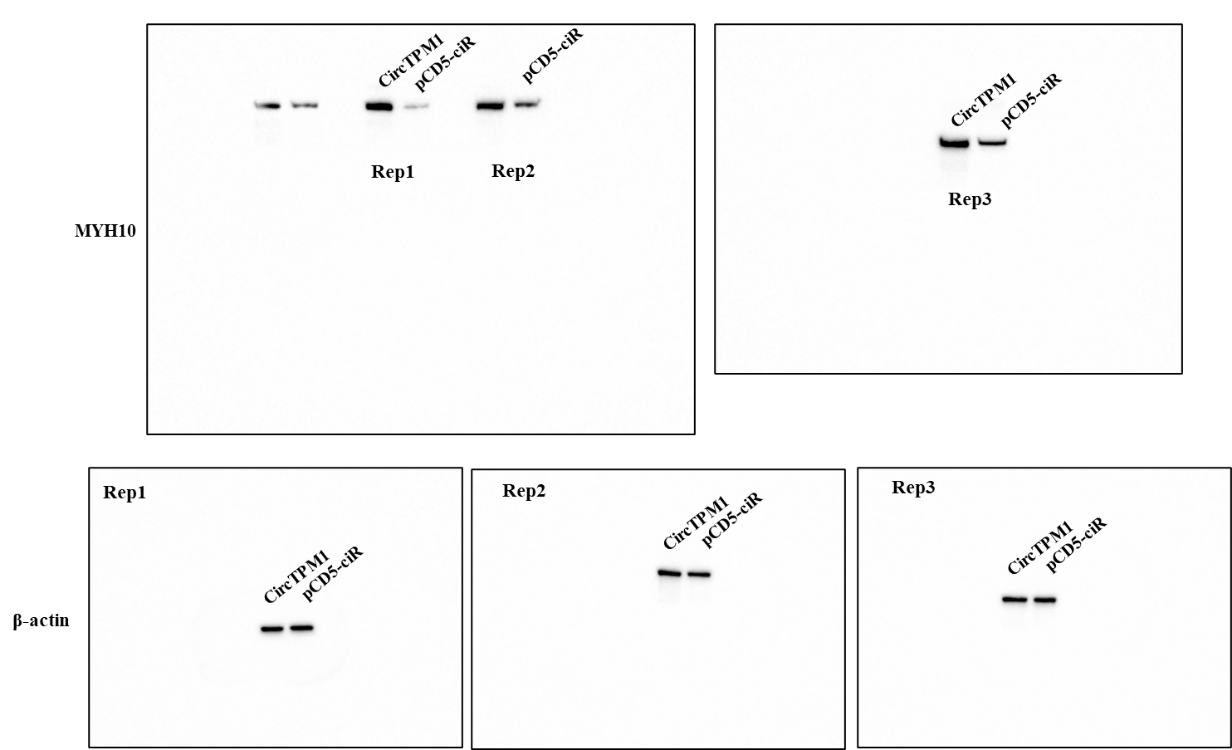


**Figure 7H**


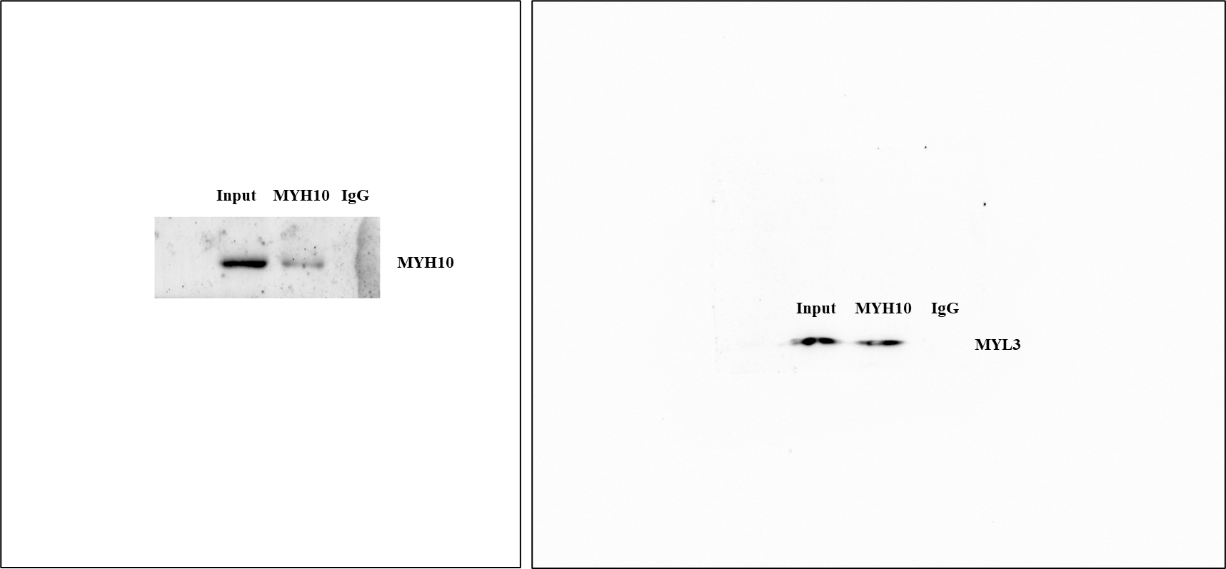


**Figure 7I**


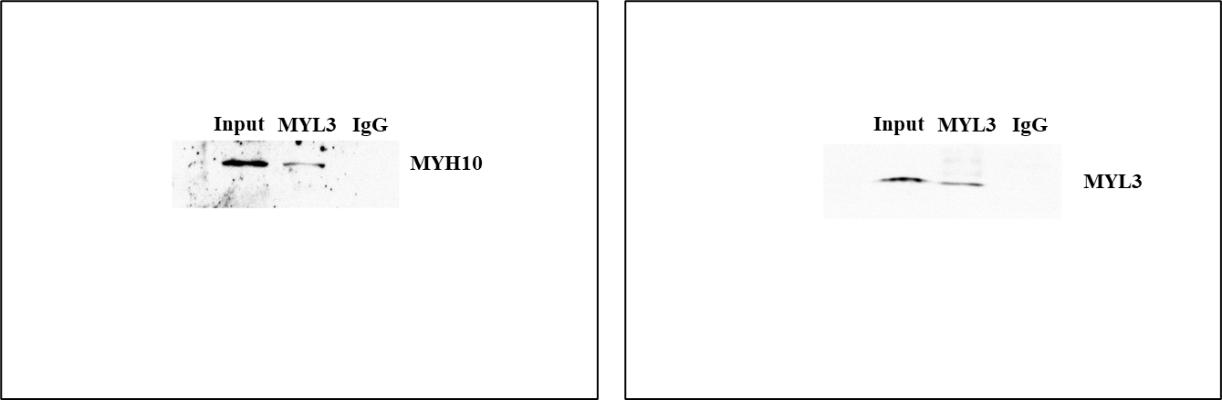


**Figure 7K**


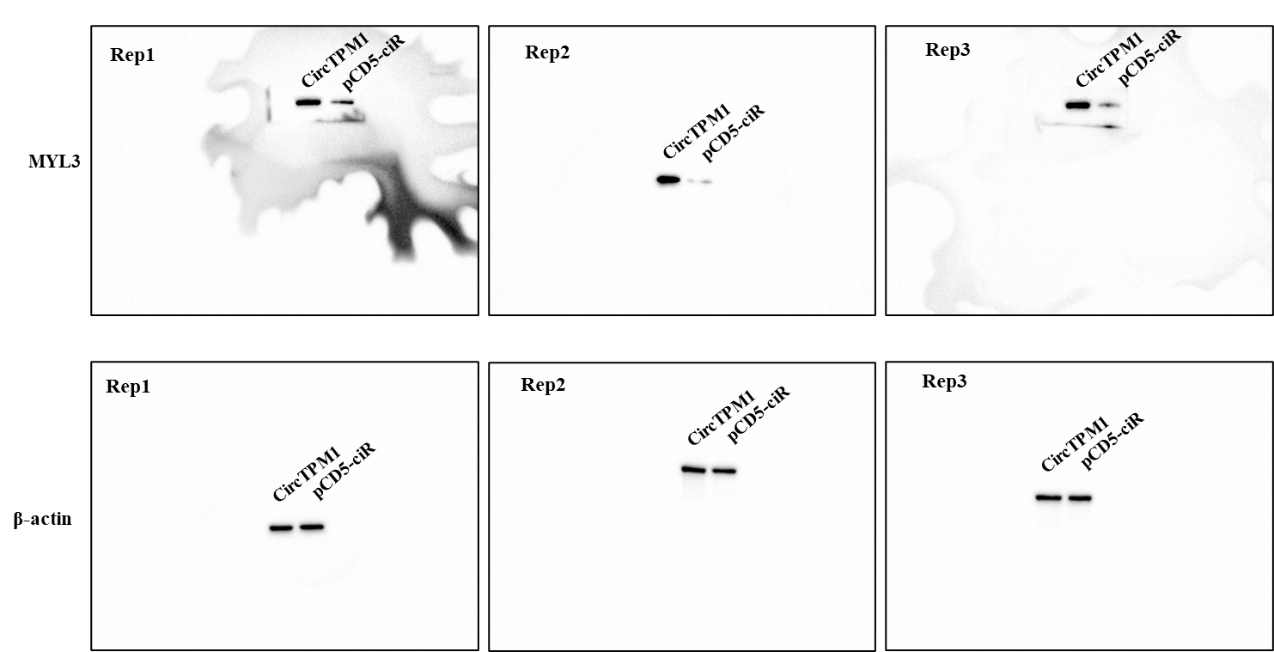


**Figure 7O**


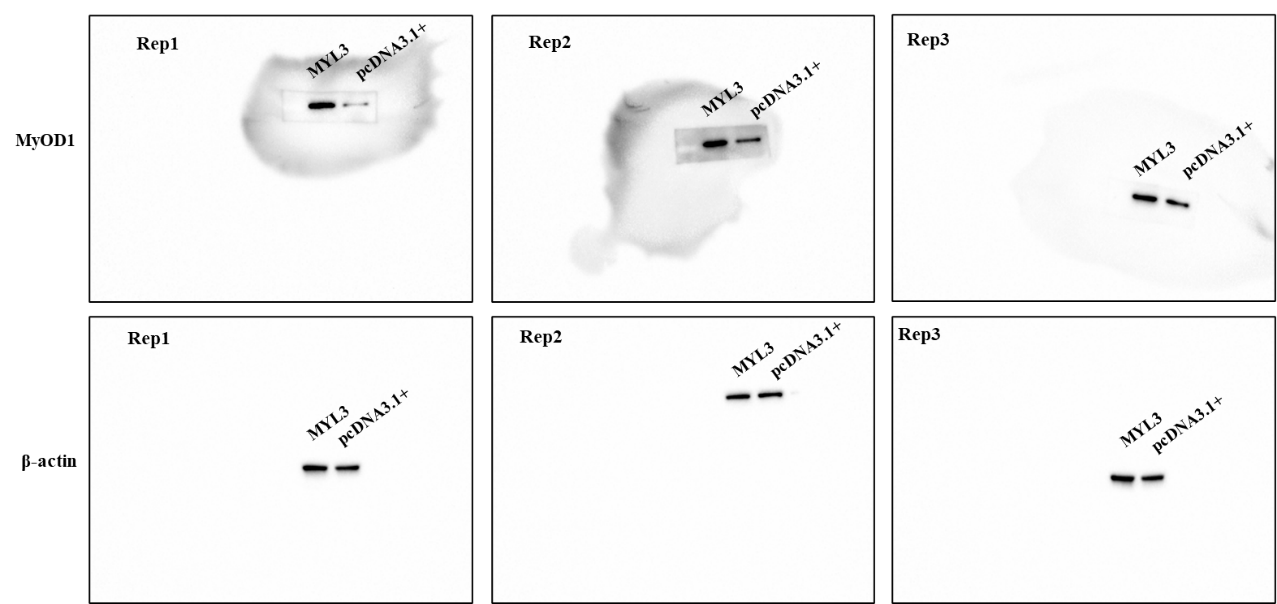


**Figure 7Q**


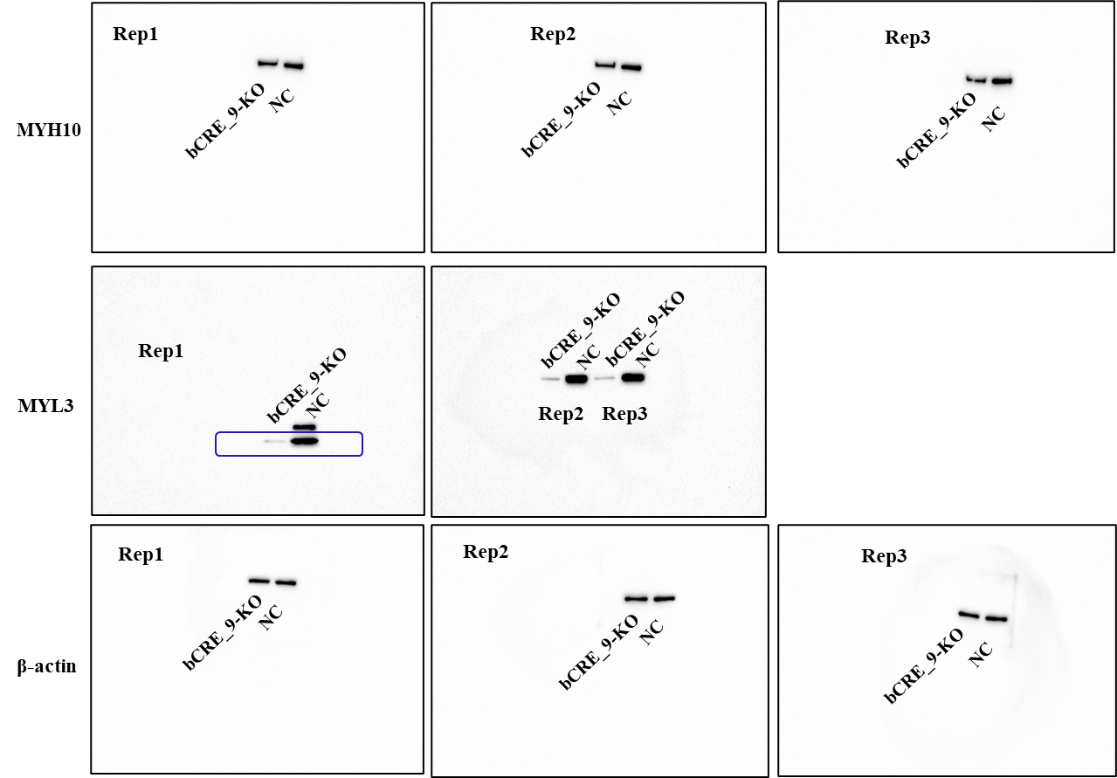


**Figure S9K**


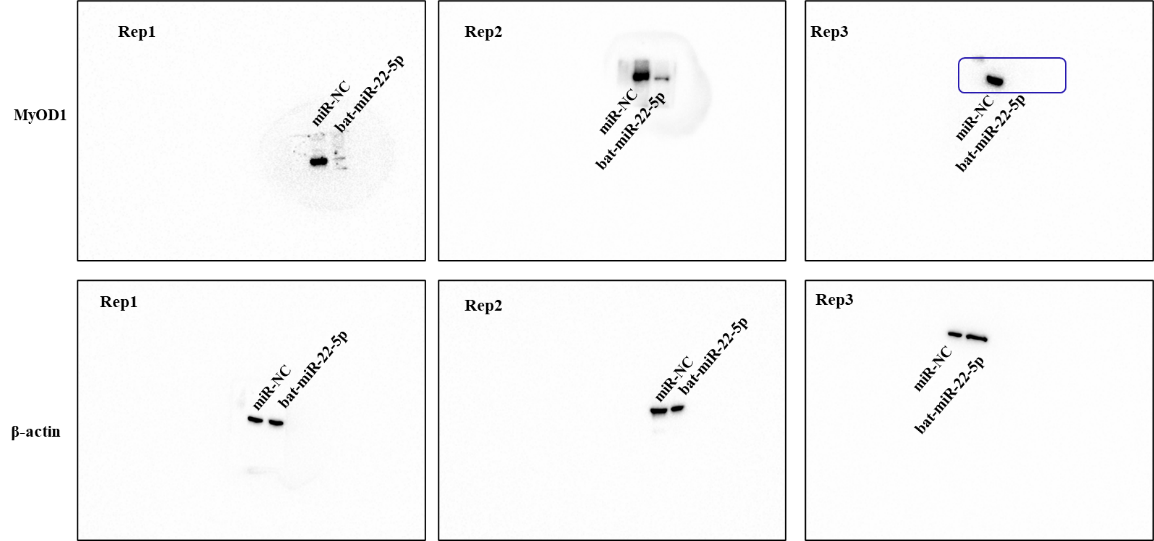


**Figure S10D**


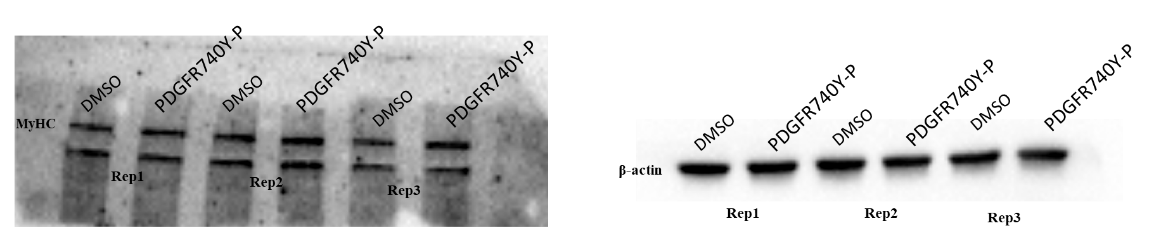


**Figure S10G**


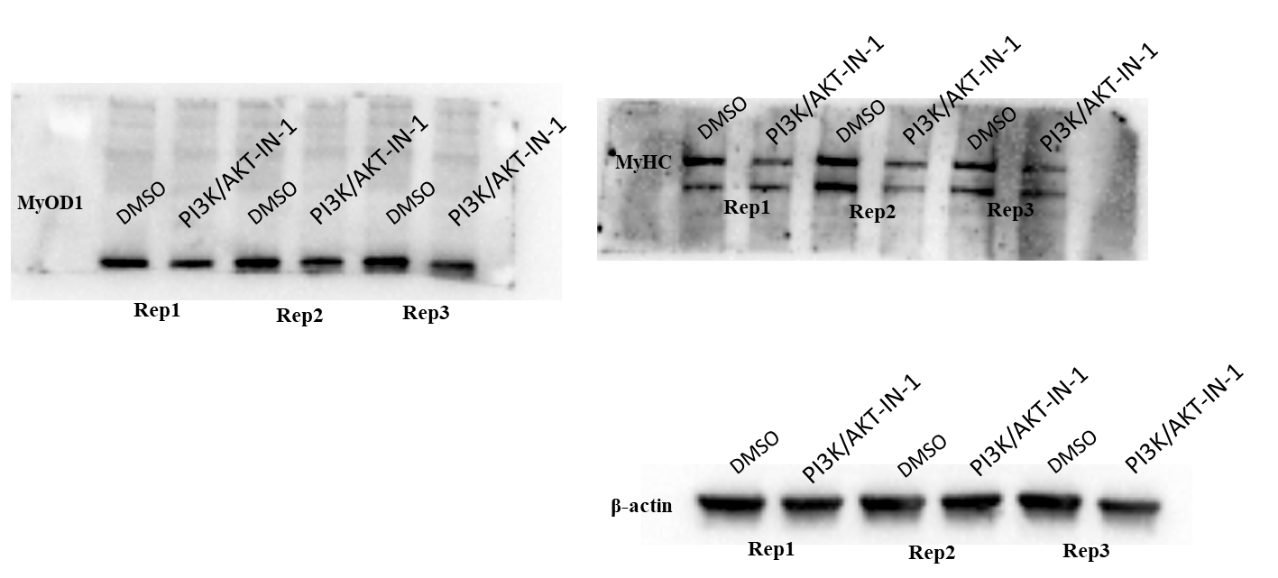


**Figure S10L**


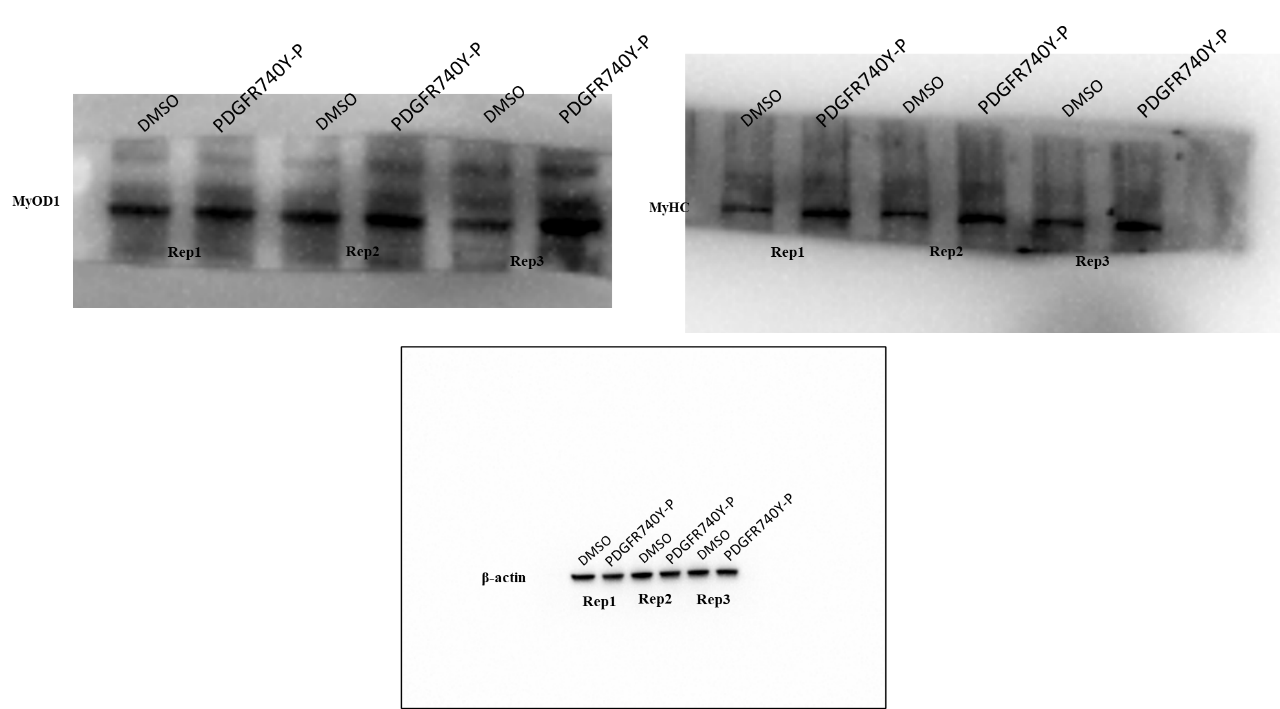


**Figure S10O**


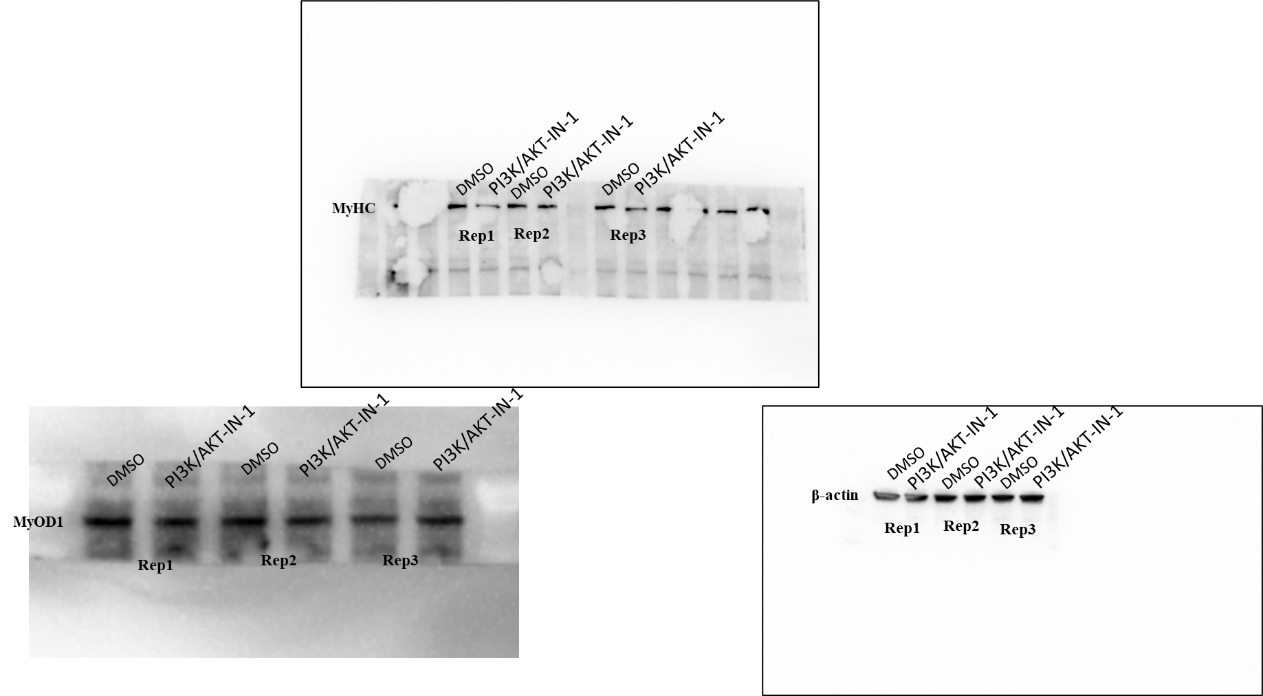


**Figure S11B**


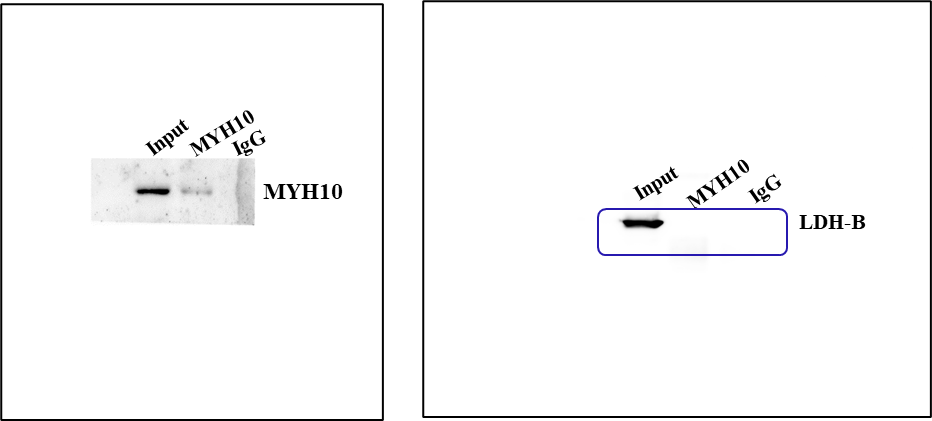


# Supplementary table caption

Supplementary Table 1(Table S1)

Description: distribution and number of bovine MuSCs-related SEs on the genome.

Supplementary Table 2(Table S2)

Description: distribution and number of human skeletal muscle myoblasts related SEs on the genome.

Supplementary Table 3(Table S3)

Description: distribution and number of mice C2C12 myoblasts related SEs on the genome.

Supplementary Table 4(Table S4)

Description: SEs of bovine, human, and murine associated genes

Supplementary Table 5(Table S5)

Description: KEGG pathway enrichment analysis of SE regulated genes

Supplementary Table 6(Table S6)

Description: body weight growth in mice.

Supplementary Table 7(Table S7)

Description: bovine MuSCs associated differentially expressed circRNAs.

Supplementary Table 8(Table S8)

Description: analysis of the binding site of TPM1_SE to the transcription factor TEAD4.

Supplementary Table 9(Table S9)

Description: analysis of miRNA binding sites

Supplementary Table 10(Table S10)

Description: mass spectrometric analysis of CircTPM1 pull-down precipitates

Supplementary Table 11(Table S11)

Description: mass spectrometric analysis of MYH10 pull-down precipitates (CoIP-MS)

Supplementary Table 12(Table S12)

Description: list of oligonucleotides used in the study

Key resources table

Description: antibodies, chemicals, peptides, recombinant proteins, plasmids, and others used in this study.
